# Single-cell transcriptomic landscape of nucleated cells in umbilical cord blood

**DOI:** 10.1093/gigascience/giz047

**Published:** 2019-05-02

**Authors:** Yi Zhao, Xiao Li, Weihua Zhao, Jingwan Wang, Jiawei Yu, Ziyun Wan, Kai Gao, Gang Yi, Xie Wang, Bingbing Fan, Qinkai Wu, Bangwei Chen, Feng Xie, Jinghua Wu, Wei Zhang, Fang Chen, Huanming Yang, Jian Wang, Xun Xu, Bin Li, Shiping Liu, Yong Hou, Xiao Liu

**Affiliations:** 1 School of Biology and Biological Engineering, South China University of Technology, Guangzhou 510006, China; 2 BGI-Shenzhen, Shenzhen 518083, China; 3 Shenzhen Second People's Hospital, First Affiliated Hospital of Shenzhen University, Shenzhen 518035, Guangdong Province, China; 4 Shanghai Institute of Immunology, Shanghai JiaoTong University School of Medicine, Shanghai 200025, China; 5 James D. Watson Institute of Genome Sciences, Hangzhou 310058, China; 6 Department of Immunology and Microbiology, Shanghai JiaoTong University School of Medicine, Shanghai 200025, China

**Keywords:** umbilical cord blood, single-cell RNA sequencing, transcriptomics, nucleated red blood cell, natural killer T cell

## Abstract

**Background:**

For both pediatric and adult patients, umbilical cord blood (UCB) transplant is a therapeutic option for a variety of hematologic diseases, such as blood cancers, myeloproliferative disorders, genetic diseases, and metabolic disorders. However, the level of cellular heterogeneity and diversity of nucleated cells in UCB has not yet been assessed in an unbiased and systemic fashion. In the present study, nucleated cells from UCB were subjected to single-cell RNA sequencing to simultaneously profile the gene expression signatures of thousands of cells, generating a rich resource for further functional studies. Here, we report the transcriptomes of 17,637 UCB cells, covering 12 major cell types, many of which can be further divided into distinct subpopulations.

**Results:**

Pseudotemporal ordering of nucleated red blood cells identifies wave-like activation and suppression of transcription regulators, leading to a polarized cellular state, which may reflect nucleated red blood cell maturation. Progenitor cells in UCB also comprise 2 subpopulations with activation of divergent transcription programs, leading to specific cell fate commitment. Detailed profiling of cytotoxic cell populations unveiled granzymes B and K signatures in natural killer and natural killer T-cell types in UCB.

**Conclusions:**

Taken together, our data form a comprehensive single-cell transcriptomic landscape that reveals previously unrecognized cell types, pathways, and mechanisms of gene expression regulation. These data may contribute to the efficacy and outcome of UCB transplant, broadening the scope of research and clinical innovations.

## Introduction

Human umbilical cord blood (UCB) is an excellent source of hematopoietic progenitor cells. It has been widely used for bone marrow reconstitution since the 1980s [1, 2]. The progenitor cells contained in UCB can regenerate the entire lympho-hematopoietic compartment in the host. The most notable advantage of UCB transplant is the low risk of developing graft-versus-host disease, even when donor and recipient are partially mismatched [3]. The immune cells in UCB are virtually free from external stimulant and infection and thus are relatively more naïve. Such immunological immaturity is the key to alleviating the severity of graft-versus-host disease by decreasing the alloreactive potential of lymphocytes [2, 4]. These advantages expand the clinical potential of UCB transplant in many cases, including some fatal diseases. The major limitation of UCB transplant, however, is the limited and inconsistent cell dose. It has been shown that the success rate of engraftment was critically dependent on the number of nucleated cells in the donor UCB [4–6].

Although UCB is now widely used for important clinical applications, we know surprisingly little about its cellular and molecular characteristics. Specifically, the composition of progenitor, lymphocyte, and other nucleated cells that affect the reconstitution potency after UCB engraftment is poorly understood. Recent advances in single-cell transcriptomics technology enable the exploration of cellular heterogeneity and deduction of functional relevance [7, 8]. Single-cell RNA sequencing (RNA-seq) studies of human peripheral blood (PB) cells have revealed new insights into immune cell composition and disease-related functional abnormalities [9–11]. Previous studies in mouse and human have focused on hematopoietic stem cell (HSC), erythroblast, and certain T-cell subtypes, unveiling novel biological properties at the single-cell level [12–17]. However, single-cell RNA-seq studies have not thoroughly characterized the major types of nucleated cells in UCB, especially erythrocytes and cytotoxic innate immune cells, despite their profound clinical significance. Thus, the present study aimed to investigate the nucleated cells present in UCB to depict a landscape view of the cellular composition and their transcriptomes. Such key information will undoubtedly facilitate clinical innovation to develop more efficient and cost-effective UCB transplantation.

## Results

### A single-cell transcription atlas of nucleated cells in umbilical cord blood

To acquire a transcriptomic map of UCB cells at single-cell resolution, we collected samples of UCB from 2 healthy donors and isolated nucleated cells for single-cell RNA-seq using the 10 × Chromium platform. After stringent quality control and filtering by multiple criteria (see Methods), transcriptomes of 7,852 and 9,785 single cells from the 2 UCB samples (UCB1 and UCB2) were acquired, detecting a mean of 1,270 and 1,460 genes per cell, respectively. To determine the unique cell subpopulations and the specific state of gene expression in UCB, we used the public single-cell transcriptomics dataset of PB cells for comparison. This dataset includes 2 independently generated libraries (PB1 and PB2), containing a total of 11,948 single-cell profiles of peripheral blood mononuclear cells (PBMCs) measuring 1,069 genes per cell on average. These are at a comparable level with those of the UCB data.

All 4 single-cell datasets were merged to enable a systematic comparison between UCB and PB cells. To identify cell populations on the basis of their expression signatures, we analyzed the merged data using a typical pipeline in the Seurat software, including dimensionality reduction and subsequent unsupervised cell clustering [18]. However, when the data were visualized in a 2D space by t-distributed stochastic neighborhood embedding (tSNE), we initially observed a strong segregation of UCB cells from PB cells regardless of cell type—a typical manifestation of batch effect. We also noticed that a group of UCB cells (3.92% of all UCB cells) that express massive amounts of hemoglobin genes, such as *HBG1* and *HBM* (Supplementary Fig. S1A and B), tend to significantly interfere with the merging of UCB cells with PB cells and cell clustering, generating highly sample-segregated cell embeddings in the tSNE space (data not shown). Thus, prior to merging with the PB data, we excluded these cell clusters, which were later identified as nucleated red blood cells (NRBCs) and were further analyzed.

To isolate biological variance from the interfering technical variances in the remaining data, we employed 3 independent computational methods, canonical correlation analysis (CCA) [19], surrogate variable analysis (SVA) [20], and mutual nearest neighbors (MNN) [21], to systemically correct the potential technical variance (Supplementary Fig. S2A–D). We then quantitatively evaluated the corrected data using an alignment score−based method [19]. Results indicated that the MNN algorithm most successfully eliminated the batch effect in the current dataset (Supplementary Fig. S2E and F). Thus, we proceeded to use MNN-corrected expression matrices for the Seurat pipeline and all subsequent analysis.

**Figure 1:**
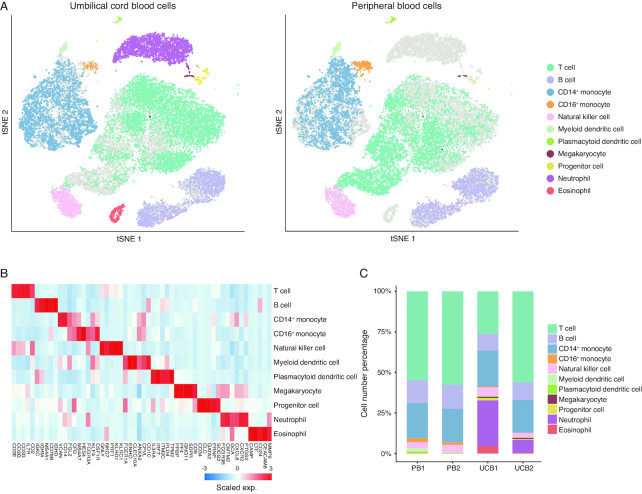
Cell types identified in the UCB. **(A)** Global tSNE plots of merged UCB and PB cells. Cell clusters are colored to indicate cell types by expressed known markers. UCB cells are colorized in the left panel, and PB cells are colorized in the right panel. Cell types and their respective colors are labeled on the right. **(B)** Heat map of scaled mean gene expression (exp.) of the major canonical markers (columns) detected in different cell types in merged cells of UCB and PB (rows). **(C)** Distribution of the abundance of each cell in each cell type in the PB and UCB datasets.

A global view was generated to illustrate the cell composition landscape of UCB. Aside from the NRBCs, 11 distinct cell populations were clustered—based on their gene expression profiles—in both UCB samples. A merged PB dataset was clustered in parallel with UCB cells in the same tSNE space (Fig. 1A). All of the clusters identified were shared by the 2 UCB samples, demonstrating the robustness of our biological replicate (Supplementary Fig. S2D). Clusters of cells expressing known markers of major immune cell types were assigned with their respective identities (Fig. 1B, Supplementary Fig. S3A). The expression patterns of a few representative marker genes are shown as examples (Supplementary Fig. S3B). To further validate the cell type annotations, we calculated transcriptome-wide correlations between cluster mean expression and previously characterized bulk RNA-seq profiles of sorted immune cell types, as reported in previous studies [22], which was in accordance with the annotation yielded by canonical marker genes (Supplementary Fig. S4A). Nine major immune cell types and hematopoietic lineages found in PB were identified in UCB, while neutrophils, eosinophils, and the bioinformatically excluded NRBCs were only present in the UCB data. The neutrophil and eosinophil discrepancy was expected because of different cell enrichment approaches used (see Methods) (Fig. 1C, Supplementary Fig. S4B). We focused the scope of the present study on a few cell types with profound clinical applications. However, the cellulome landscape of UCB data constitutes a rich resource that can be used as a reference to complement transcriptomics analysis performed in bulk or single-cell settings, as well as a guide to future functional studies.

### Polarity of cord nucleated red blood cells

**Figure 2:**
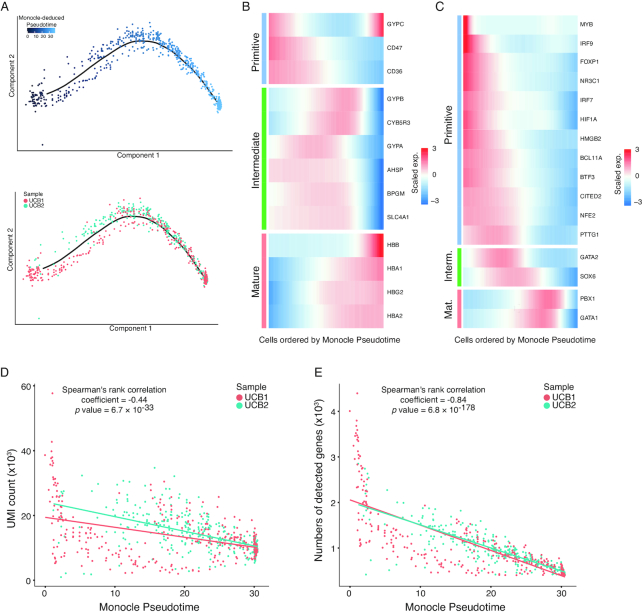
Polarity of nucleated red blood cells in the UCB samples. **(A)** The order of NRBCs along pseudotime in a 2D space determined by Monocle2. Each dot represents a single NRBC. Color gradient represents the pseudotemporal order in the upper panel. Cells from the 2 UCB samples are labeled in the same topology in the bottom panel. **(B)** Heat map of gene expression (exp.) in NRBCs ordered by pseudotime (x-axis). Three clusters of pseudotime-dependent genes are grouped into primitive stage (top), intermediate stage (middle), and mature stage (bottom). **(C)** Heat map of key transcription factor (TF) expression, similar to (B). **(D)** Numbers of detected unique molecular indices (UMIs) in each NRBC ordered by pseudotime. Each dot represents an NRBC, and the color represents the corresponding UCB sample of each cell. Y-axis represents number of detected UMIs (thousands). Overall Spearman's correlation coefficient and corresponding *P* values are shown at the top. **(E)** Numbers of detected genes in each NRBC, ordered by pseudotime. Each dot represents an NRBC, and the color represents the corresponding UCB sample of each cell. Y-axis represents the number of detected genes (thousands). Overall Spearman's correlation coefficient and corresponding *P* values are shown at the top.

In mammalian hematopoiesis, NRBCs, or erythroblasts, undergo several developmental stages in the bone marrow, progressively decreasing cellular volume and RNA content, while accumulating specific functional proteins such as hemoglobin [23, 24]. It has long been known that erythroblasts exist in relatively large numbers in UCB [25–27]. However, little is known about whether such developmental processes exist in the UCB, or whether the erythroblast population is homogenous. In our dataset, we found that NRBCs constitute a significant proportion of the total nucleated cells in UCB (Supplementary Fig. S4B). Interestingly, NRBCs in the UCB samples displayed pronounced polarity defined by the divergent expression of a gene repertoire. We used Monocle2 software to identify differential genes among NRBCs and deduced a pseudotemporal ordering of the cells that suggested a gradual change of cellular state [28] (see Methods). Evidently, the NRBCs from both UCB samples formed a linear trajectory along the pseudotime axis, with no significant branching, indicating that the cell polarity resulted from continuous changes of gene expression (Fig. 2A). To further validate the dual polarity of NRBCs in UCB, we employed an independent approach to construct a diffusion pseudotime map based on the transitions between cells using diffusion-like random walks [29] (Supplementary Fig. S5A). Cell ordering along the trajectories deduced by the 2 algorithms showed remarkable concordance (Supplementary Fig. S5B).

Next, we modeled gene expression along the Monocle2-inferred trajectory to identify genes characterized by a wave-like pattern. The most prominent were those genes encoding surface markers and proteins that are critical to the function of red blood cells, such as CD47, CD36, hemoglobin, and glycophorins [30] (Fig. 2B). The CD47 molecule has long been considered to be a cell surface marker of primitive erythrocytes [31]. Hemoglobin genes, in contrast, are highly expressed in the relatively mature form of NRBCs. Thus, the polarity observed here most likely reflected the maturity state of the NRBCs. An intermediate cell state that bridges the naïve state (CD47 high) and the mature state (hemoglobin high) was also observed. This intermediate stage was characterized by the elevated expression of a set of genes including those encoding glycophorins (*GYPA* and *GYPB*), suggesting that the cells in this stage exerted a specific function, rather than being just transient intermediates. Strikingly, several key transcriptional regulators of erythrocyte homeostasis, including GATA1/2 and BCL11A [32–34], also clearly exhibited divergent patterns along the pseudotime axis (Fig. 2C). GATA1 is a well-characterized transcription factor (TF) responsible for the activation of multiple hemoglobin-encoding genes in erythroid ontogeny [35], while BCL11A is a TF that silences hemoglobin-encoding genes [34]. Other examples were CITED2 and SOX6, TFs that have recently been characterized as signature molecules specifically expressed in mouse primitive and definitive erythroblasts, respectively. These showed similar specificity in the naïve and intermediate cellular states, as defined by the pseudotime axis [36]. To provide further evidence of this model, a gradual decrease in the numbers of RNA molecules (represented by unique molecular indices [UMIs]) (Fig. 2D) and expressed genes (Fig. 2E) across the pseudotime axis was observed, and permutation analyses demonstrated significant correlation between the gradual decrease and the pseudotemporal ordering of the cells (Supplementary Fig. S5C). This potentially reflects diminishing global gene expression activity caused by NRBC enucleation. These lines of evidence further corroborated the polarity identified in the NRBC population in UCB projected maturation progress and strongly indicated that the differential activation of transcriptional programs was one of the underlining mechanisms.

### Molecular signatures of UCB progenitor cells

**Figure 3:**
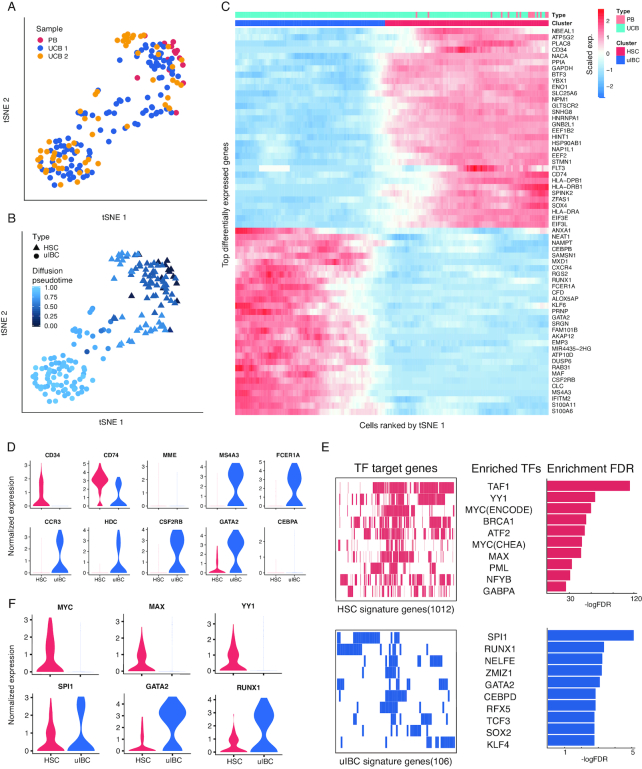
Heterogeneous molecular signatures of progenitor cells in UCB. **(A)** The re-clustered tSNE projection of progenitor cells from UCB and PB samples. The samples are labeled with different colors for each cell. **(B)** The 2 cell clusters, HSCs and uIBCs, are represented by triangles and dots, respectively. The color gradient represents the pseudotemporal order. **(C)** Heat map of differentially expressed signature genes in the progenitors. Cells along the x-axis were ordered in the same way as in tSNE 1 axis in (A). The color bar on top denotes the HSC and uIBC clusters as well as the corresponding samples. **(D)** Violin plots of exemplary feature gene expressions of HSC (red) and uIBC (blue) cells. **(E)** Transcription factor enrichment analysis of HSC and uIBC cells using HSC signature genes (1,012 genes, top left) and uIBC signature genes (106 genes, bottom left) revealed enriched transcription factors (TFs) in HSC (top middle) and uIBC (bottom middle). Bar graphs of corresponding enrichment scores (−log false discovery rate [FDR]) are shown on the right. **(F)** Violin plots of exemplary enriched TF expression in HSC (red) and uIBC (blue) cells.

A distinct progenitor population was found in UCB, which shared a similar transcriptome profile with the HSCs in the PB dataset (Fig. 1A, Supplementary Fig. S4A). However, when tSNE clustering was performed with the progenitor population at a finer resolution, a secondary subpopulation emerged, demonstrating the heterogeneity of the progenitor population in the UCB (Fig. 3A). One subpopulation of UCB progenitor cells overlapped with HSCs in PB and specifically expressed canonical HSC marker genes such as *CD34, SOX4*, and *FLT3* (*CD135*) (Fig. 3B, triangles), suggesting their identity as UCB HSCs. Interestingly, the other subpopulation comprised cells only from the UCB (Fig. 3B, dots) and did not express the HSC canonical markers (Fig. 3C and D) despite the similarity in the overall spectrum of gene expression, which drove the clustered embeddings of these cells in the tSNE space. Surprisingly, this CD34^–^ UCB-specific progenitor population highly expressed the myeloid lineage−specific gene *MS4A3* (Fig. 3D), a known signature of granulocytic-monocytic progenitors (GMPs) [37]. GMPs give rise to mast cell progenitors (MCP) and basophil progenitors, which are found in the bone marrow, spleen, and gastrointestinal mucosa [38]. Furthermore, *FCER1A*, the gene encoding the Fc fragment of the IgE receptor, which is also a surface marker frequently used in cell sorting for mast cells [39], was highly expressed in the CD34^–^ cell population, while *CCR3*, a sorting marker for basophils [40, 41], was co-expressed at a comparable level. Similarly, many genes with regulatory roles in mast cell and basophil differentiation, exemplified by *HDC* and *CSF2RB*, respectively [16, 38, 42], were also co-expressed at a high level (Fig. 3D). The concerted activation of gene repertoires critical in GMP-MCP and GMP−basophil progenitor ontogeny axes strongly suggested that these cells were bi-potent progenitors or intermediate cells, similar to the basophil/mast cell progenitor (BMCP) first verified in spleens of adult mice [43]. A high level of GATA2 and a low level of CEBPA TFs was also consistent with the signatures of mouse BMCP [43–45] (Fig. 3D). Such expression signatures are also reminiscent of recently identified basophil/eosinophil/mast cell progenitors (Ba/Eo/Ma) in human UCB and bone marrow [16, 46]. A critical difference between the UCB subpopulation and the mouse BMCP or human Ba/Eo/Ma is that *CD34* expression is turned off, suggesting limited stemness and differentiation commitment in these cells. We thus hypothesized that these cells represent intermediates before bifurcation during basophil and mast cell differentiation; we termed them umbilical intermediate bi-potent cells (uIBCs). To further explore this hypothesis, we sought to use diffusion maps [29, 47] to characterize the trajectory of the speculated transition from HSC to u

IBC. While a gradual shifting of identities from HSC to uIBC was observed on the first diffusion component, the uIBC side of the trajectory did not show a conclusive bifurcation towards mast cell and basophil lineages; this is likely because of the limited cell number (Supplementary Fig. S5D).

Next, we asked whether the switch of cell identities resulted from the alteration of transcriptional programming governing the differentiation process. TF enrichment analysis utilizing the Encode [48] and ChEA [49] databases was performed to detect overrepresented combinations of conserved TF binding sites in a given set of genes. The analysis revealed that TAF, YY1, and MYC were mostly enriched for activating highly expressed genes found in the HSCs compared with uIBC (Fig. 3E). These TFs are well known for their roles in proliferation and cell cycle control [50–53]. Conversely, RUNX1, SPI1, and GATA2 were ranked as the top-enriched TFs for activating highly expressed genes in the uIBCs (Fig. 3E). These TFs are conventionally considered to be master regulators of differentiation of the myeloid lineage [44, 54, 55]. Such functional correlation was further corroborated by the mutually exclusive expression pattern of the top-enriched factors. For example, high expression levels of *MYC, MAX*, and *YY1*, enriched for activating HSC feature genes, were detected in the HSCs; and—vice versa—high expression levels of *SP1, GATA2*, and *RUNX1* were detected in the u

IBC (Fig. 3F). These lines of evidence supported the conclusion that the 2 subtypes of cells we found in the progenitor population in UCB were divergent on the hematopoietic axis and may have UCB-specific functions.

### Heterogeneity of cytotoxic innate immune cells

**Figure 4:**
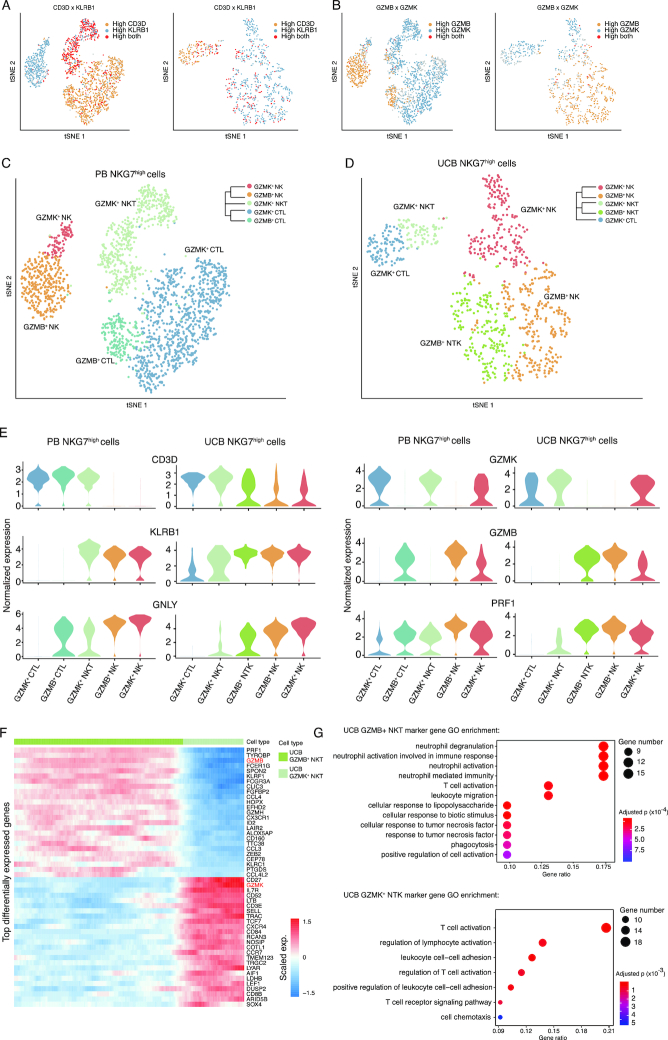
Heterogeneity of cytotoxic cells in PB and UCB. **(A)** t-distributed stochastic neighbor embedding (tSNE) plots of re-clustered cytotoxic cells from PB (left) and UCB (right) datasets. Each dot represents a single cytotoxic cell. Yellow color demonstrates high expression of CD3D; blue indicates KLRB1 highly expressed cell; red indicates cells highly expressing both CD3D and KLRB1; gray indicates cells expressing neither gene. **(B)** The tSNE plots with the same topology as in (A), with the gradient colors demonstrating the expression of GZMB and GZMK. **(C)** tSNE plots of cytotoxic cells from the PB datasets. Cell subtypes (GZMK^+^ CTL, granzyme B-positive [GZMB^+^] CTL, granzyme K-positive [GZMK^+^] NKT, GZMB^+^ NK, GZMK^+^ NK) are labeled with different colors. **(D)** tSNE plots of cytotoxic cells from the UCB datasets. Cell subtypes (GZMK^+^ CTL, GZMK^+^ NKT, GZMB^+^ NKT, GZMB^+^ NK, GZMK^+^ NK) are labeled with different colors. **(E)** Violin plots of signature gene expression of the subtypes in UCB (right) and PB (left). Color labeling of cell subtypes is consistent with that in (C). **(F)** Heat map of exemplary differentially expressed signature genes in the GZMB^+^ NKT and GZMK^+^ NKT subtypes. The color bar on top denotes the GZMB^+^ NKT and GZMK^+^ NKT subtypes. **(G)** Gene ontology (GO) analysis of differentially expressed signature genes specific to GZMB^+^ NKT (upper panel), and to GZMK^+^ NKT (bottom panel) subtypes in UCB. The most enriched GO terms are ordered on the y-axis. X-axis represents the gene percentage in enriched GO terms. Sizes of the dots represent the number of genes included in each GO term. The color gradient of dots represents the adjusted *P*-values of each enriched GO term.

Effective immune response to infection, allergy, and cancer generally requires coordinated activation of the innate and adaptive immune systems. Recent studies have shown that natural killer (NK) T cells emerge as a bridge between innate and adaptive immunity to mediate immune responses [56]. In the overall tSNE projection, NK cells were clustered as a contiguous “peninsula” extending from the T-cell population (Fig. 1A). Interestingly, *KLRB1*, a lineage marker of NK cells, was expressed in a gradient pattern across the 2 cell types, with no distinct boundary (Supplementary Fig. S6A). Remarkably, the expression of *CD3D/E* was in a reversed gradient with that of *KLRB1* (Supplementary Fig. S6B), as well as those of the cytotoxic genes *NKG7, PRF1*, and *GNLY* (Supplementary Fig. S6C). This pattern of expression indicated the existence of a group of cells with a bridging identity across the interface, most likely NKT cells. Unlike NK or T cells, NKT cells exhibit distinct tissue specificity under homeostatic conditions, suggesting compartmentalized functions [57–60]. To selectively investigate these cells, we used high-resolution clustering results generated by Seurat (see Methods), producing more detailed clusters of T and NK cells (Supplementary Fig. S6D), 2 of which corresponded to NK cells, the adjacent T cells, and the bridging NKT cells that displayed gradient expression of *CD3D/E* and *KLRB1* (Supplementary Fig. S6E). The T cells in this cluster expressed CD8 but not CD4 and thus were considered to be cytotoxic T cells (Supplementary Fig. S7A). We next carried out subclustering with these cells to further reveal heterogeneity. By relative expression levels of the lineage markers, and the fact that all of these cells express a spectrum of cytotoxic marker genes, such as *NKG7, PRF1*, and *GNLY*, at high levels (Supplementary Fig. S6A-C), we assigned the cell identity as cytotoxic T lymphocytes (CTLs) (CD3^+^CD4^−^CD8^+^KLRB1^–^), NK (CD3^–^KLRB1^+^), and NKT (CD3^+^KLRB1^+^) cells [58, 61, 62] (Fig. 4A).

Although CTL, NK, and NKT cells were all present in the PB and UCB samples, the cell compositions were rather different. Apparent heterogeneity was observed in all 3 cell lineages and—remarkably—was represented by the mutually exclusive expression of 2 granzyme genes, *GZMB* and *GZMK* (Fig. 4B). For example, the NK and CTL cells in PB were each divided into 2 subgroups, specifically expressing *GZMB* and *GZMK* (Fig. 4C). Similarly, NK and NKT cells in UCB were also subgrouped into granzyme K-positive (GZMK^+^) and granzyme B-positive (GZMB^+^) populations (Fig. 4D). Thus, based on the expression of lineage markers and the 2 granzyme genes used for this classification scheme (Fig. 4E), a total of 6 distinct cell subtypes were defined. All subtypes found in UCB were consistent between donors (Supplementary Fig. S7B); however, both UCB donors lacked GZMB^+^ CTL cells that were present in PB, possibly because of the lack of specific antigen stimulation. It was noteworthy that GZMB^+^ NKT cells were abundantly detected in UCB but were missing in PB, begging the question as to whether this particular subtype possessed specific functions. Collectively, the cell distribution of NKT cells and CTLs indicated that UCB has stronger innate immunity and less adaptive immunity compared with PB. NKT cells were previously reported to have tissue-specific gene expression programs that lead to diverse functions and were termed NKT1, NKT2, and NKT17, predominantly localized in the liver, lung, and peripheral lymph nodes, respectively [58, 63–66]. In our data, the expression profile of GZMB^+^ NKT cells was mostly similar to that of the NKT1 type, highlighted by signature expression of *CD44, KLRB1, ZBTB16, IL2RB*, and *TBX21* (Supplementary Fig. S7C). However, neither GZMB^+^ nor GZMK^+^ cells expressed *GATA3*, a crucial TF found in NKT2 and NKT17 cells [67, 68]. Together with the lack of *KLRB1* expression, the GZMK^+^ NKT-cell subtype is distinct from the known NKT2 or NKT17 subtypes [67, 68]. The enriched GZMB^+^ NKT cells in UCB express a spectrum of chemokines and genes in cytotoxic pathways, which may mediate recruitment with other immune cell types to coordinate an innate immune response (Fig. 4F). Gene ontology (GO) analysis further corroborated that the highly expressed genes of the GZMB^+^ cells were enriched in innate cytotoxic immunity, such as neutrophil-mediated immunity, cellular response to infectious antigens, and necrosis factors, while GZMK^+^ cells were enriched in lymphocyte activation, lymphocyte cell–cell adhesion, and chemotaxis pathways (Fig. 4G). Thus, we concluded that the cell composition of NKT and other cytotoxic cells varies between PB and UCB.

**Figure 5:**
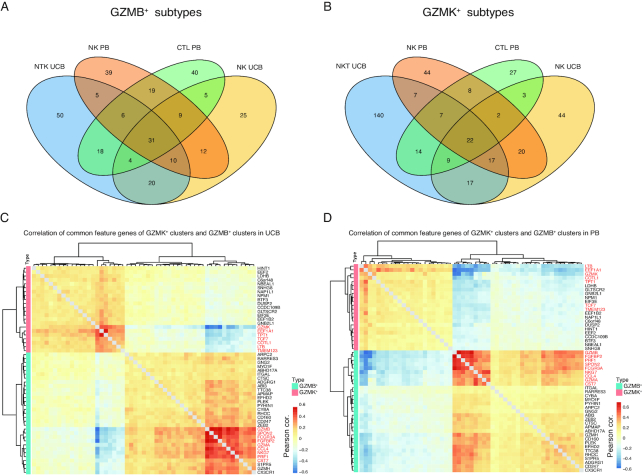
Enrichment of feature genes of granzyme B and K subtypes. **(A)** Four-way Venn diagrams reveal the enrichment of feature genes among granzyme B (GZMB)-positive cell types. **(B)** Four-way Venn diagrams reveal the enrichment of feature genes among granzyme K (GZMK)-positive cell types. **(C)** Pearson's correlation (cor.) of expression of the 4-way−overlapped genes in (A) and (B) from UCB datasets. **(D)** Pearson's correlation of expression of the 4-way−overlapped genes in (A) and (B) in PB datasets.

Unlike NKT, GZMK^+^ and GZMB^+^ NK subtypes were both present in PB and UCB (Fig. 4C and D). They may function differently because of their respective granzyme gene activation [69]. Recent studies have shown that the orchestrated expression of granzymes is part of the functional program that enables cytotoxic cells to exert specific functions [70, 71]. As exemplified by the NK subtypes, *GZMB* and *GZMK* expression represents such functional diversity and highlights their respective cytotoxic gene expression programs. To reveal the elements of these 2 programs, we systemically compared the GZMB^+^ subtypes of NK, NKT, and CTL cells found in PB or UCB by testing the co-occurrence of signature genes specific to each subtype (see Methods). Among the 4 sets of signature genes—ranging from 116 to 144 in number—31 signature genes were found to be shared by all 4 subtypes (Fig. 5A). Similarly, 22 signature genes were found to be common in the corresponding GZMK^+^ subtypes (Fig. 5B). Permutation tests were performed to estimate the significance of the 4-way intersection in both cases, and the resulting *P*-values were both <3 × 10^−16^. These 2 sets of signature genes (31 and 22) that we found were defined as GZMB and GZMK co-expressed genes, respectively, which were likely to contribute to the elimination of specific antigens. To corroborate these findings, we calculated the Pearson's correlation of cell-averaged expression of all 53 genes in GZMB^+^ and GZMK^+^ subtypes of NK and NKT cells in UCB, and CTL and NK cells in PB. As expected, unsupervised clustering revealed 2 major modules, corresponding to the GZMB and GZMK programs (Fig. 5C and D). Interestingly, within each program a smaller core module was discovered, highlighted by *EEF1A1, TPT1, COTL1*, and *LTB* in the GZMK program and *FGFBP2, PRF1, GZMA, FCGR3A*, and *CCL4* in the GZMB program (Fig. 5C, red labeled genes). Similar analysis was performed in the PB cells. Here, the core modules were largely consistent with those of UCB, although the GZMK core module was less prominent (Fig. 5D, red labeled genes). The enriched genes identified in the 2 programs represent common features of the GZMB^+^ and GZMK^+^ subtypes of cytotoxic cells. They may serve as specific selection markers and targets for perturbation in further functional studies.

## Discussion

Here, we present for the first time a single-cell−level transcriptomic landscape of nucleated cells in UCB. By analyzing the expression patterns of known marker genes, we identified UCB cells belonging to almost all of the major hematopoietic lineages in PB, covering lymphoid, myeloid, and hematopoietic progenitor cells. We also observed that certain cell populations were highly enriched in UCB cells, such as NRBCs, uIBCs, and GZMB^+^ NKT cells. The features of these cells that we discovered were consistent in both UCB donors. However, it is important to keep in mind that the UCB donors’ shared factors, such as genetic background, may contribute to the enrichment of these UCB-specific cell subtypes. A related technical challenge that we encountered in the present study was the severe batch effect among sample types and donors. To minimize any technical variance that could lead to misinterpretation of the data, we rigorously tested 3 widely used algorithms for batch effect correction, namely, CCA, SVA, and MNN. Based on a quantitative evaluation of cell segregation in the tSNE space, MNN and CCA appeared comparable and effective for our datasets, although MNN scored marginally higher.

In adults, red blood cells are mainly generated in the bone marrow from nucleated cells, which are identified as erythroid precursors. These cells undergo morphological changes throughout cell divisions, gradually decreasing in cell size and RNA species and increasing in chromatin condensation and hemoglobin protein accumulation. Such changes have been associated with the early stages of maturation of red blood cells. In our dataset, we also observed such a dynamic cellular state in a linear polarity. While it is possible that the erythroid precursors at different stages in UCB may have migrated from the bone marrow, our findings also suggested that erythroid precursors might undergo a similar maturation process in the UCB.

Progenitor cell populations in UCB also appeared to be a mixture of ≥2 distinct subpopulations. It is conceivable that the HSC subpopulation (CD34^+^) that we identified is a mixture of HSCs and various early multipotent progenitors committed to differentiation; these were termed “primed progenitors” and were extensively discussed in a recent profiling study of UCB HSCs at the single-cell level [16]. The lack of CD34 enrichment means that the UCB data in the present study have too few HSCs to recapitulate the heterogeneity reported in this study. The uIBC, a unique UCB subpopulation not seen in PB, was identified, with characteristics of both basophil and mast cell signatures. A similar bi-potent population (BMCP) exists in mouse spleen and is capable of divergent development [43]. Signature gene expression, including TFs and surface markers, was remarkably similar between BMCP and uIBC, except that u

IBCs lack expression of the conventional progenitor marker CD34. Although u

IBCs and HSCs in UCB were globally similar in their transcriptomic profiles, the lack of CD34 made it difficult to conclude whether or not these u

IBCs were indeed progenitors, or transient intermediates captured during UCB hematopoiesis. The functional implication of their existence points to the developmental process downstream of the Ba/Eo/Ma primed branch detected in the previous study [16]—specifically, when the Ba/Eo/Ma primed cells lose stemness markers (e.g., CD34) and further express lineage genes. Functional validations are necessary to determine the potential abilities of self-renewal and lineage regeneration of these cells and to substantiate the similarity with mouse BMCP or Ba/Eo/Ma primed cells at the functional level.

Next, we interrogated the UCB single-cell data at a finer scale and discovered unreported heterogeneity among CTL, NK, and NKT cells in UCB, which appeared in different compositions and granzyme expression patterns to those in PB. It is noteworthy that the mutually exclusive pattern between the GZMA/B/perforin program versus the GZMK program was a common feature in cytotoxic cell lineages in UCB and PB. This finding is consistent with previous studies performed in PB [69], demonstrating that human granzymes are differentially expressed in distinct subpopulations that may function outside of orchestrating cytotoxicity. Multiple recent studies utilizing single-cell technologies have found that diversified expression of granzyme genes is indicative to T-cell states under disease conditions, such as liver cancer, colorectal cancer, non−small cell lung cancer, and HIV-1 infection. The consensus is that *GZMB*-expressing T cells tend to recapitulate the transcriptome of effective memory T cells, and *GMZK*-expressing T cells seem to be a transitional intermediate between the effective and exhausted states [11, 71–73]. It is clear that such a pattern is not specific to disease conditions because we now have shown that similar granzyme programs exist in UCB CTL/NKT/NK cells as well. However, we did not find significant expression of exhausted marker genes in UCB GMZK^+^ CTLs, possibly because of the lack of constant antigen stimulus. The specification of GZMK^+^ and GZMB^+^ cells is likely to reflect different consecutive activation states, which might be interchangeable upon changes of the tissue microenvironment. Interestingly, a previously unknown NKT population, which may be unique to UCB, was identified as GZMB^+^ NKT cells that do not express *GZMK* but highly express *GZMA, GZMH*, and *PRF1* genes instead, suggesting the activation of specific cytotoxicity mediated by granzyme and perforin pathways. NKT cells play an essential role in bridging innate and adaptive immunity against infectious diseases and tumorigenesis; thus, they possess significant therapeutic value. UCB transplants have demonstrated remarkable effectiveness in treating many types of blood cancers. Adoptive transfer of NKT cells has been tested in animal models [74, 75], and several clinical trials are underway to test the safety and efficiency of NKT-cell transfer to harness solid tumors in humans [76–79]. Enhanced understanding of NKT-cell heterogeneity in UCB would benefit our selection of appropriate sources, and activation of the cytotoxicity of NKT cells to target cancer and other diseases. Therefore, we speculated that a targeted enrichment, modulation, or engineering of the existing NKT populations in UCB could lead to considerable improvement in the efficacy of enhancing protective immune responses.

### Potential implications

Taken together, our data provide the first single-cell transcriptomic references for UCB, which could be used as a standard dataset for comparative analysis. We expect that this dataset will prove useful in uncovering the novel molecular signatures that define the cellular heterogeneity of UCB and will provide markers for targeted enrichment of certain cell types of interest to researchers in multiple fields. Our dataset is a rich resource to formulate hypotheses of signaling pathway activation, transcription control, and other mechanistic studies in the field of functional immunology at the single-cell level.

## Methods

### Sample collection

The project was reviewed and approved by the BGI institutional review board and the ethics committee of Shenzhen Second People's Hospital (No. 18,120). Two UCB samples were collected from healthy donors immediately after Cesarean delivery with informed consent. Samples were stored in ethylenediaminetetraacetic acid (EDTA) anticoagulant tubes and transported to the laboratory within 1 hour. CD45^+^ and CD45^–^ cells were isolated from 1 mL UCB by positive and negative selection, respectively, using Whole Blood CD45 MicroBeads (Miltenyi,130–090-872, USA) and a Whole Blood Column Kit (Miltenyi, 130–093-545, USA). Next, a hemocytometer was used to count CD45^+^ and CD45^–^ cells, and these were mixed in a ratio of 4:1. The cells were gently pipetted into a single-cell suspension and diluted to a concentration of 700 cells/µL. Public single-cell gene expression datasets of PBMCs (PB1 and PB2) were generated from a sample from a single donor. In the present study, PB1 and PB2 correspond to Cell Ranger 2.0.1-processed “8k PBMCs from a healthy donor” and “4k PBMCs from a healthy donor,” respectively [80].

### UCB library construction and sequencing

Single-cell suspensions of UCB samples were loaded to chips from the Single Cell 3′ Chip Kit (10x Genomics, CA, USA) and subjected to the GemCode Single Cell Instrument (10x Genomics) to generate single-cell gel beads in emulsion, as per the manufacturer's instructions. Next, gel beads in emulsion were subjected to library construction using Chromium™ Single Cell 3′ Reagent Kits v2 (10x Genomics), the steps of which included incubation at room temperature, complementary DNA amplification, fragmentation, end repair, A-tailing, adaptor ligation, and sample index polymerase chain reaction. Because this library was designed to be sequenced by the Illumina sequencing platform, we converted the libraries to be compatible with the BGISEQ-500 sequencer. To do so, we performed a 12-cycle polymerase chain reaction on the libraries using BGISEQ adaptor primers, with subsequent DNA circularization and rolling-cycle amplification to generate DNA nanoballs. Purified DNA nanoballs were sequenced using the BGISEQ-500 sequencer, generating reads containing 16 base pairs of 10x™ barcodes, 10 base pairs of UMIs, and 100 base pairs of 3′ complementary DNA sequences. Each library was sequenced in 3 lanes, yielding ∼1.9 billion reads in total [81–83].

### Alignment and initial processing of sequencing data

The CellRanger toolkit (10x Genomics, USA, version 2.0.0) was used to align complementary DNA reads to the Genome Reference Consortium human build patch 38 (GRCh38) transcriptome. Filtered UMI expression matrices of both samples were generated using default parameters, and an additional “–force-cells = 4000” parameter [84]. The expression matrices of all samples were first normalized using the “cellranger aggr” function in the CellRanger toolkit, with the parameter “–normalize = mapped.” As a result, raw expression data were generated for ∼32,000 single cells of the UCB sample.

### Quality filtration of cells

In accordance with published pipelines and quality control standards [18], abnormal cells in all datasets were uniformly filtered out on the basis of their gene expression distribution. A cell was considered to be abnormal if any of the following criteria were met: (i) detected gene number <400; (ii) detected gene number >2,000, >2,000, >3,500, and >3,000 for PB1, PB2, UCB1, and UCB2 datasets, respectively; and (iii) >8%, >8%, >6%, and >7% of detected genes are mitochondria genes in PB1, PB2, UCB1, and UCB2 datasets, respectively. A “detected gene” is defined as any gene expressed in ≥30 individual cells at a level of UMI ≥1 in any given dataset. After filtering the PB1, PB2, UCB1, and UCB2 datasets, 8,380, 3,977, 8,981, and 9,638 cells remained, respectively.

### Cell clustering in individual UCB samples

Next, the filtered expression matrices of UCB1 and UCB2 were used for unsupervised cell clustering using Seurat (version 2.3.4), adopting the typical pipeline recommended by the authors (Seurat, RRID:SCR_016341) [18]. A total of 3,113 (UCB1) and 2,409 (UCB2) variable genes were used for the “RunPCA” function. Subsequently, the top 10 principal components (PCs) were subjected to the “FindClusters” and “RunTSNE” functions, with high-resolution setting at 2.0 (Supplementary Fig. S1A). In the dimensionally reduced tSNE space, clusters of NRBCs were identified on the basis of the concerted expression of hemoglobin genes, such as *HBG1* and *HBM* (Supplementary Fig. S1B). Then, we bioinformatically isolated 672 NRBCs from UCB1 and UCB2 as a subdataset for further analyses. NRBC-excluded data were then subjected to merging and batch effect removal. NRBCs were excluded prior to data merging because we noticed that the massively expressed hemoglobin genes significantly interfered with the merging of UCB cells with PB cells and cell clustering, yielding highly sample-segregated cell embeddings in the tSNE space, regardless of batch removal methods or parameters used.

### Correction of batch effects

Strong technical bias introduced by sample preparation, library construction, and/or sequencing was observed in the merged data (Supplementary Fig. S2A). To evaluate the available strategy for batch correction, we independently tested SVA, CCA, and MNN analysis and compared their outcomes. For the SVA method, we first log-transformed the expression values [as in log(exp + 1)], then used the ComBat function in the SVA package to minimize batch effects, with default parameters [20]. CCA was performed in Seurat to correct batch effects. Having tested different parameters, we observed the best performance when we used 15 canonical vectors and 1,500 shared, high-variable genes.

For MNN, we first created a SingleCellExperiment object to store the counts and metadata for each sample, using SingleCellExperiment (version 1.3.10). These cells were pre-clustered using the quickCluster function. Size factors were computed for endogenous genes using the deconvolution method by computeSumFactors [85]. We then acquired normalized log-expression values and distinguished highly variable genes using the trendVar function, and decomposed the gene-specific variance into biological and technical components using the decomposeVar function. To obtain a single set of features for batch correction, we computed the average biological component across all 4 batches. All genes with positive biological components were retained to ensure that biological variance was preserved. All batches were rescaled to account for differences in sequencing depth using the multiBatchNorm function. Last, the fastMNN function was applied to each of the 4 samples, using retained genes with the parameters k = 50, d = 50, approximate = TRUE, and auto.order = TRUE. Finally, corrected expression values for 3,570 highly variable genes were generated using the tcrossprod function, and these expression values were used for downstream cell clustering and pseudotime analysis.

### Evaluation of batch correction

Alignment scores of the aforementioned methods were calculated based on tSNE plots, according to the strategy used in a previous study [19]. First, neutrophils and eosinophils that were only present in UCB datasets were masked from the datasets. Then, we randomly sampled cells from the 4 datasets with the same number of cells, and constructed a nearest-neighbor graph based on their relative positions in tSNE space. For each sampled cell, we calculated the cell numbers from the dataset sample in the *k* nearest neighbors and average with total cells to obtain ***x***. The alignment score was then calculated as alignment score = 1− [(***x*** − *k*/*N*)/(*k* − *k*/*N*)]. Alignment scores were normalized by dataset size and scaled to range from 0 to 1. For Supplementary Figure S2E, the parameters used were *k* = 800, *N* = 4. As shown, the MNN alignment score was marginally higher than that of CCA. To rule out potential bias from the arbitrary selection of *k*, we tested different values of *k* from 100 to 1,000 and observed that the high scores generated by MNN were independent of *k* selection (Supplementary Fig. S2F).

### Cell type annotation

After batch correction by MNN, the merged expression matrix was further filtered following the typical Seurat pipeline. Specifically, ribosomal genes and cells with a mitochondrial gene UMI percentage >10% were removed, as well as cells with a total UMI count of >11,000. Then, the expression matrix was normalized using the NormalizeData function. The corrected expression matrix was used for dimensionality reduction following the typical Seurat pipeline. Next, 3,556 variable genes in the batch-corrected expression matrix were used for RunPCA, ProjectPCA, FindClusters, and RunTSNE functions with default parameters, except dims.use = 1:13 and resolution = 2.

Subsequently, the feature genes for each cluster were identified using normalized data with the Seurat FindAllMarkers function, with parameters min.pct = 0.25, and thresh.use = 0.25. Four minor clusters with ∼5% (same as estimated by 10x Genomics) total cells, which were suspected to be doublets because they shared feature genes from 2 adjacent large clusters, were removed from the datasets. A total of 8,043, 3,905, 7,852, and 9,785 cells remained for annotation in the PB1, PB2, UCB1, and UCB2 datasets, respectively (Supplementary Fig. S4B). The identity of each cell cluster was manually annotated by the specific expression of commonly known markers. Unsupervised annotation, by comparing averaged single-cell expression levels with bulk RNA-seq data of sorted immune cells, was also performed to validate the results, as previously described [86]. Pearson's correlation was used to calculate the distance between the cell-averaged feature gene expression and the corresponding levels in bulk RNA-seq data (Supplementary Fig. S4A).

### Pseudotime analysis of NRBCs

A total of 672 NRBCs, identified from the individually clustered UCB datasets, were directly merged for the following analysis. After removing 5 abnormal cells on account of their substantially deviated mitochondrial gene expression level (>2.5%), 667 NRBCs were used to infer the developmental polarity of NRBCs. NRBCs were ordered according to the pseudotime deduced by 1,859 ordering genes excluding ribosomal protein transcripts, which were differentially expressed (FDR < 0.05), using the “clusterCells” function in Monocle2 (version 2.6.4). The genes that changed as a function of pseudotime were further identified and clustered to allow visualization of modules of genes co-varying across pseudotime, according to the typical pipeline provided in the Monocle2 manual. The cluster-representing red blood cell effector, and known development-relevant genes for the heat map plots, were further manually selected based on the literature. In parallel, a pseudotemporal trajectory was deduced using the diffusion map application programming interface (API) in Scanpy (Python 3.6.6, Scanpy 1.3.2), using default parameters (n_neighbors = 20 and n_pcs = 5 for the pre-processing.neighbors function, and n_comps = 15 for the tools.diffmap function).

Diffusion map algorithm-generated pseudotemporal ordering of cells was compared with that of Monocle2 using Spearman's rank-order correlation (Supplementary Fig. S5B). Permutation analysis was performed by randomly shuffling the pseudotemporal ordering of cells 1,000 times and generating a distribution of Spearman's rank-order correlation coefficient. Student's t-test was then applied to estimate the statistical significance of deviation of the observed coefficient produced by Monocle pseudotime from the permutation distribution.

### Clustering and pseudotime analysis of UCB progenitor cells

UCB progenitor cells were re-clustered using Seurat, as described for global clustering above. To visualize the potential transition of cell identities from HSCs to uIBCs, we used the diffusion map API in Scanpy to calculate the diffusion pseudotime trajectory; as in NRBC analysis, default parameters were used, with the exception of n_pcs = 6. Then, we used the FindAllMarkers function in Seurat, with the parameter min.pct = 0.3, to find feature genes within the 2 clusters. To identify the divergent TF programs in the 2 groups of cells, the web-based tool “Enrichr” [87] was used to analyze the enrichment of TF binding on the signature genes set of each progenitor cell group [88].

### Cytotoxic cell clustering and profiling

Cytotoxic cells of interest were selected by unsupervised clustering at resolution = 2 using the FindClusters function in Seurat (Supplementary Fig. S6D). The 2 clusters (highlighted in Supplementary Fig. S6E) covering the gradient expression of multiple cytotoxic genes in Supplementary Fig. S6C were selected to create 2 new subdatasets, according to their respective sample type. Then, the 2 sets of UMI matrices (2,271 cells in PB and 879 cells in UCB) were subjected to a typical Seurat pipeline. The Seurat functions NormalizeData, RunPCA, ProjectPCA, FindClusters, and RunTSNEfunctions, with the parameters dims.use = 1:3 and resolution = 1.5 for UCB, and dims.use = 1:8 and resolution = 1.5 for PB, were sequentially applied. Subsequently, the cluster-specific genes used to annotate cell subtypes were identified using normalized data and the Seurat FindAllMarkers function, with the parameters min.pct = 0.25 and thresh.use = 0.25.

### Signature gene selection in GZMK^+^ and GZMB^+^ subtypes

To identify common features of GZMK and GZMB (4-way−overlapped genes) programs in the cytotoxic cells (Fig. 5), GZMB/GZMK-expressing NK, NKT-cell, and CTL subtypes were used to create a new Seurat object using the SubsetData function. The function FindAllMarkers was used to identify corresponding feature genes of each cluster, with the parameters min.pct = 0.25 and thresh.use = 0.25.

The 4-way Venn diagrams of feature genes shown in Fig. 5A and B were generated using the R package VennDiagram. To verify the statistical significance of the enrichment of the 4-way−overlapped genes (GZMB/GZMK program genes), a 1-sample t-test was carried out by testing the mean number of overlapping genes from randomly sampled pools of genes. The sizes of these genes were kept the same as in the original feature genes in the 4 subtypes. The co-expression modules shown in Fig. 5C and D were identified by unsupervised clustering of Pearson's correlation of cell-averaged expression values.

### Gene ontology analysis of UCB GZMB^+^ NKT cells

To deduce the potential functions of the signature genes in UCB GZMB^+^ NKT cells, GO enrichment analysis was performed using clusterProfiler (version 3.8.1, RRID:SCR_016884) [89], with the top 100 feature genes of GZMK^+^ NKT cells in UCB identified by Seurat. We then simplified the output from enrichGO by removing redundancy of enriched GO terms with the “simplify” function.

## Availability of supporting data and materials

The raw data reported in this study are deposited in the NCBI Sequence Read Archive under bioproject No. PRJNA524398, and in the CNGB Nucleotide Sequence Archive (CNSA) (CNSA: https://db.cngb.org/cnsa/) with accession No. CNP0000090. Aligned sequences in CRAM format, and the gene expression matrix for every single cell, have also been uploaded to the *GigaScience* GigaDB repository [90].

## Additional files

**Supplementary Figure 1:** Pre-clustering of UCB samples and exclusion of NRBCs. **(A)** Pre-clustering of cells in UCB dataset 1 (UCB1; left) and UCB2 (right). Each dot represents a single cell, and cells are color-labeled by cluster in tSNE space. **(B)** tSNE plots of the normalized expression of hemoglobin genes *HBG1* (left) and *HBM* (right) in UCB1 (top) and UCB2 (bottom). The color gradient represents the relative expression level.

**Supplementary Figure 2:** Sample distribution and evaluation of batch correction methods. **(A)** Sample distribution in tSNE space before batch removal processing. Cells are color-labeled by sample. **(B–D)** Sample distribution in tSNE space after CCA (B), Combat (C), and MNN (D) processes. Cells are color-labeled by sample in the same way as in (A). **(E)** Bar plot of alignment scores produced by different methods as shown in (A–D). **(F)** Comparison of alignment scores between CCA and MNN, with different parameters (from k = 100 to k = 1,000).

**Supplementary Figure 3:** Signature gene expression of each cell type. **(A)** Heat map of the scaled average gene expression of signature genes (column) detected in different cell types in UCB and PB (rows). **(B)** tSNE plots of the normalized expression of marker genes in the same global topology as in Fig. 1A. Each dot represents a single cell, and the color gradient represents the normalized gene expression.

**Supplementary Figure 4:** Cell type annotation composition. **(A)** Pearson's correlation between cell-averaged feature gene expression, with the corresponding levels in bulk RNA-seq data generated in sorted cells. **(B)** Table of cell numbers and percentages of different cell types in each sample.

**Supplementary Figure 5:** Pseudotime analysis in NRBCs and progenitor cells. **(A)** The order of NRBCs along pseudotime in a 2D space, as determined by diffusion map. Each dot represents a single NRBC. Color gradient represents the pseudotemporal order in the left panel. Cells from the 2 UCB samples are labeled with the same topology as in the right panel. **(B)** Correlation between the pseudotemporal ordering of cells using Monocle and diffusion map. The correlation coefficient was calculated by Spearman's rank testing. **(C)** Permutation analysis of Spearman's correlation coefficient, as generated by Monocle pseudotemporal ordering (observed value) versus a distribution of coefficient generated by 1,000-time randomly shuffled pseudotemporal orderings. Red vertical lines represent the observed values. *P*-values were calculated by Student's t-test. **(D)** The order of progenitor cells along pseudotime in a 2D space, as determined by diffusion map. Each dot represents a single cell, and the color gradient represents the order of pseudotime (left). The sample distribution along the pseudotime, and the color, represents the corresponding sample (right).

**Supplementary Figure 6:** Cytotoxic signature gene expression in NK and NKT-cell populations. **(A–C)** Zoomed-in tSNE plots of the normalized expression of cytotoxicity and related genes of the cytotoxic cell. Each dot represents a single cell, and the color gradient represents normalized gene expression. **(D)** Unsupervised high-resolution clustering of merged PB and UCB cells, in the same tSNE topology as in Fig. 1A. Clusters are labeled by different colors. **(E)** As for (D), cells with cytotoxic features that are further analyzed are highlighted in blue color.

**Supplementary Figure 7:** Differential gene expression in NK and NKT-cell subpopulations. **(A)** Global expression patterns of T-cell subtype markers CD4, CD8A, and CD8B. Lower right panel illustrates the T-cell subtype distribution determined by the markers. **(B)** Cells are color-labeled by samples in the same tSNE space as in Fig. 4C and D. Each dot represents a single cell in PB (left) and UCB (right). **(C)** Violin plots show the scaled expression of indicated differential genes between granzyme B-positive (GZMB^+^) NKT-cell and granzyme K-positive (GZMK^+^) NKT-cell subsets in UCB.

GIGA-D-18-00470_Original_Submission.pdf

GIGA-D-18-00470_Revision_1.pdf

GIGA-D-18-00470_Revision_2.pdf

Response_to_Reviewer_Comments_Original_Submission.pdf

Response_to_Reviewer_Comments_Revision_1.pdf

Reviewer_1_Report_Original_Submission -- Tapio LÃ¶nnberg, Ph.D. 12/10/2018 Reviewed

Reviewer_2_Report_Original_Submission -- Antoine-Emmanuel Saliba 12/17/2018 Reviewed

Supplemental File

## Abbreviations

API: application programming interface; Ba/Eo/Ma: basophil/eosinophil/mast cell progenitors; BMCP: basophil/mast cell progenitor; CCA: canonical correlation analysis; CTL: cytotoxic T lymphocyte; FDR: false discovery rate; GMP: granulocytic-monocytic progenitor; GO: gene ontology; GZMB^+^: granzyme B-positive; GZMK^+^: granzyme K-positive; HSC: hematopoietic stem cell; MCP: mast cell progenitor; MNN: mutual nearest neighbors; NK: natural killer; NRBC: nucleated red blood cell; PB: peripheral blood; PBMC: peripheral blood mononuclear cell; SVA: surrogate variable analysis; TF: transcription factor; tSNE: t-distributed stochastic neighborhood embedding; UCB: umbilical cord blood; u

IBC: umbilical intermediate bi-potent cell; UMI: unique molecular indices.

## Competing interests

Some of the authors are employed by BGI, including Y.Z., X. Li, Jingwan Wang, J.Y., Z.W., K.G., X.W., Q.W., B.C., J. Wu, W. Zhang, H.Y., F.C., Jian Wang, X.X., B.L., X. Liu, Y.H., and S.L. The other authors declare no competing interests.

## Funding

This work was supported by the Shenzhen Municipal Government of China (grant Nos. JCYJ20170817145404433 and JCYJ20170817145428361).

## Authors’ contributions

X. Liu, Y.H., and S.L. jointly supervised the research. Y.Z., B.L., and G.Y. designed the experiments. X.W., K.G., Y.Z., and X.Z. performed the experiments. Y.Z. and Jingwan Wang pre-processed the sequencing data. Y.Z., X. Li, Jian Wang, Z.W., and Jingwan Wang analyzed the data. W. Zhao and B.F. collected the cord blood. X. Li, Y.Z., and X. Liu wrote the manuscript. X. Liu, Q.W., B.C., H.Y., F.C., Jian Wang, W. Zhang, X.X., and F.X. revised the manuscript. All authors reviewed and approved the final manuscript.
