# Supplementary material for: Single-cell transcriptomic landscape of nucleated cells in umbilical cord blood
Source: Gigascience. 2019 May 2;8(5):giz047. doi: 10.1093/gigascience/giz047 (PMC6497034; doi:10.1093/gigascience/giz047)
Supplement: GIGA-D-18-00470_Original_Submission.pdf [file giz047_giga-d-18-00470_original_submission.pdf]

GigaScience

Single-cell Transcriptomic Landscape of Nucleated Cells in Umbilical Cord Blood

--Manuscript Draft--

**Manuscript Number:**	GIGA-D-18-00470
**Full Title:**	Single-cell Transcriptomic Landscape of Nucleated Cells in Umbilical Cord Blood
**Article Type:**	Research
**Funding Information:**	
**Abstract:**	Umbilical cord blood (UCB) transplant is a therapeutic option for both pediatric and adult patients with a variety of hematologic diseases such as several types of blood cancers, myeloproliferative disorders, genetic diseases, and metabolic disorders. However, the level of cellular heterogeneity and diversity of nucleated cells in the UCB has not yet been assessed in an unbiased and systemic fashion. In the current study, nucleated cells from UCB were subjected to single-cell RNA sequencing, a technology enabled simultaneous profiling of the gene expression signatures of thousands of cells, generating rich resources for further functional studies. Here, we report the transcriptome of 17,637 UCB cells, covering twelve major cell types. Many of these cell types are comprised of distinct subpopulations. Pseudotemporal ordering of nucleated red blood cells (NRBC) identifies wave-like activation and suppression of transcription regulators, leading to a polarized cellular state, which may reflect NRBC maturation. Progenitor cells in UCB also consist two subpopulations with divergent transcription programs activated, leading to specific cell-fate commitment. Detailed profiling of cytotoxic cell populations unveiled granzymes B and K signatures in NK and NKT cell types in UCB. Collectively, we provide this comprehensive single-cell transcriptomic landscape and show that it can uncover previously unrecognized cell types, pathways and gene expression regulations that may contribute to the efficacy and outcome of UCB transplant, broadening the scope of research and clinical innovations.
**Corresponding Author:**	Xiao Liu<br><br>CHINA
**Corresponding Author Secondary Information:**	
**Corresponding Author's Institution:**	
**Corresponding Author's Secondary Institution:**	
**First Author:**	Xiao Liu
**First Author Secondary Information:**	
**Order of Authors:**	Xiao Liu
	Yi Zhao
	Xiao Li
	Weihua Zhao
	Jingwan Wang
	Jiawei Yu
	Ziyun Wan
	Kai Gao
	Gang Yi
	Xie Wang
	Qinkai Wu
	Bangwei Chen

Powered by Editorial Manager® and ProduXion Manager® from Aries Systems Corporation

	Jinghua Wu
	Wei Zhang
	Fang Chen
	Huanming Yang
	Jian Wang
	Xun Xu
	Bin Li
	Shiping Liu
	Yong Hou
	Bingbing Fan
**Order of Authors Secondary Information:**	
**Additional Information:**	
**Question**	**Response**
Are you submitting this manuscript to a special series or article collection?	No
**Experimental design and statistics**<br><br>Full details of the experimental design and statistical methods used should be given in the Methods section, as detailed in our Minimum Standards Reporting Checklist. Information essential to interpreting the data presented should be made available in the figure legends.<br><br>Have you included all the information requested in your manuscript?	Yes
**Resources**<br><br>A description of all resources used, including antibodies, cell lines, animals and software tools, with enough information to allow them to be uniquely identified, should be included in the Methods section. Authors are strongly encouraged to cite Research Resource Identifiers (RRIDs) for antibodies, model organisms and tools, where possible.<br><br>Have you included the information requested as detailed in our Minimum Standards Reporting Checklist?	Yes

**Availability of data and materials**<br><br>All datasets and code on which the conclusions of the paper rely must be either included in your submission or deposited in publicly available repositories (where available and ethically appropriate), referencing such data using a unique identifier in the references and in the “Availability of Data and Materials” section of your manuscript.<br><br>Have you have met the above requirement as detailed in our Minimum Standards Reporting Checklist?	Yes

Powered by Editorial Manager® and ProduXion Manager® from Aries Systems Corporation

Manuscript Click here to download Manuscript UCB ms 181119.docx

Single-cell Transcriptomic Landscape of Nucleated Cells in Umbilical Cord Blood

Yi Zhao[1,2,†], Xiao Li[2,†], Weihua Zhao[3,†], Jingwan Wang[2], Jiawei Yu[2], Ziyun Wan[2], Kai Gao[2], Gang Yi[4], Xie Wang[2], Bingbing Fan[2], Qinkai Wu[2], Bangwei Chen[2], Feng Xie[4], Jinghua Wu[2], Wei Zhang[2], Fang Chen[2], Huanming Yang[2,5], Jian Wang[2,5], Xun Xu[2], Bin Li[2,4], Shiping Liu[2], Yong Hou[2,*] and Xiao Liu[2,*]

[1]School of Biology and Biological Engineering, South China University of Technology, Guangzhou, 510006, China.

[2]BGI-Shenzhen, Shenzhen 518083, China

[3]Shenzhen Second People's Hospital, First Affiliated Hospital of Shenzhen University, Shenzhen 518035, Guangdong Province, China.

[4]Shanghai Institute of Immunology, Shanghai JiaoTong University School of Medicine, Shanghai 200025, China; Department of Immunology and Microbiology, Shanghai JiaoTong University School of Medicine, Shanghai 200025, China

[5]James D. Watson Institute of Genome Sciences, Hangzhou 310058, China

[†]Yi Zhao, Xiao Li and Weihua Zhao contributed equally

*co-corresponding authors

ABSTRACT

Umbilical cord blood (UCB) transplant is a therapeutic option for both pediatric and adult patients with a variety of hematologic diseases such as several types of blood cancers, myeloproliferative disorders, genetic diseases, and metabolic disorders. However, the level of cellular heterogeneity and diversity of nucleated cells in the UCB has not yet been assessed in an unbiased and systemic fashion. In the current study, nucleated cells from UCB were subjected to single-cell RNA sequencing, a technology

enabled simultaneous profiling of the gene expression signatures of thousands of cells, generating rich resources for further functional studies. Here, we report the transcriptome of 17,637 UCB cells, covering twelve major cell types. Many of these cell types are comprised of distinct subpopulations. Pseudotemporal ordering of nucleated red blood cells (NRBC) identifies wave-like activation and suppression of transcription regulators, leading to a polarized cellular state, which may reflect NRBC maturation. Progenitor cells in UCB also consist two subpopulations with divergent transcription programs activated, leading to specific cell-fate commitment. Detailed profiling of cytotoxic cell populations unveiled granzymes B and K signatures in NK and NKT cell types in UCB. Collectively, we provide this comprehensive single-cell transcriptomic landscape and show that it can uncover previously unrecognized cell types, pathways and gene expression regulations that may contribute to the efficacy and outcome of UCB transplant, broadening the scope of research and clinical innovations.

KEY WORDS

Umbilical cord blood, Single-cell RNA-seq, Transcriptomics, Nucleated red blood cell, Natural Killer T cell

INTRODUCTION

Human umbilical cord blood (UCB) is an excellent source of hematopoietic progenitor cells. It has been widely used for bone marrow reconstitution for decades [1, 2]. The progenitor cells contained in UCB are capable of regenerating the entire lympho-hematopoietic compartment in the host. The most notable advantage of UCB transplant is the low risk of developing graft-versus-host disease (GVHD), even when donor and recipient are partially mismatched [3]. The immune cells in cord blood are virtually free from external stimulant and infection and thus are in a relatively more naïve stage. Such immunological immaturity is the key to alleviate the severity of GVHD by decreasing the alloreactive potential of lymphocytes [2, 4]. These advantages expand the clinical potential of UCB transplant in many cases including some fatal diseases. The major limitation of UCB transplant, however, is the limited and inconsistent cell dose. It has been shown that the success rate of engraftment was critically dependent on the number of nucleated cells in the donor UCB [4-6].

Although UCB is now widely used for important clinical applications, we know surprisingly little about its cellular and molecular characteristics. Especially, the composition of progenitor, lymphocyte and other nucleated cells that affect the reconstitution potency after UCB engraftment is poorly understood. Recent advances in single-cell transcriptomics technology enable the exploration of cellular heterogeneity and deduction of functional relevance [7, 8]. Single-cell RNA-seq (scRNA-seq) studies of human peripheral blood (PB) cells have revealed new insights into immune cell composition and disease-related functional abnormalities [9-11].

Previous studies conducted in mouse and human have focused on hemopoietic stem cell, erythroblast and certain T cell subtypes and unveiled novel biological properties at single-cell level[12-17]. However, scRNA-seq studies have not thoroughly characterized the major types of nucleated cells in UCB, especially erythrocyte and cytotoxic innate immune cells, despite their profound clinical significance. Thus, the purpose of the current study is to investigate the nucleated cells present in UCB and depict a landscape view of the cellular composition and their transcriptomes. Such key information will undoubtedly facilitate the clinical innovation to develop more efficient and cost-effective UCB transplant treatments.

RESULT

A single-cell transcription atlas of nucleated cells in umbilical cord blood

To acquire a transcriptomic map of UCB cells at single-cell resolution, we collected UCB from two healthy donors and isolated nucleated cells for single-cell RNA-sequencing using 10× Chromium platform. After stringent quality control and filtering by multiple criteria (see Methods), transcriptomes of 7,852 and 9,785 single cells from the two UCB samples (UCB1 and UCB2) were acquired, detecting on average 1,270 and 1,460 genes per cell, respectively. To determine the unique cell subpopulations and the specific state of gene expression in UCB, we utilized the public single-cell transcriptomics dataset of peripheral blood (PB) cells for comparison. This dataset includes two independently generated libraries (PB1 and PB2), containing total of 11,948 single-cell profiles of peripheral blood mononuclear cells (PBMC) measuring

1,069 genes per cell on average, which are at comparable level with those of the UCB data.

All four single-cell datasets were merged to enable a systematic comparison between UCB and PB cells. To identify cell populations based on their expression signatures, we analyzed the merged data using a typical pipeline in the Seurat software, including dimensionality reduction and subsequent unsupervised cell clustering [18]. However, when the data were visualized in a two-dimensional space by t-distributed stochastic neighborhood embedding (tSNE), we initially observed a strong segregation of UCB cells from PB cells regardless of cell types, a typical manifestation of batch effect. We also noticed that a group of UCB cells (3.92% of all UCB cells) that express massive amount of hemoglobin genes, such as *HBG1* and *HBM* (Supplementary Fig. 1A and B), tend to significantly interfere the merging of UCB cells with PB cells and cell clustering, generating highly sample-segregated cell embeddings in the tSNE space (data not shown). Thus, prior to the merging with PB data we excluded these cells clusters, which were later identified as nucleated red blood cells (NRBCs) and were further analyzed. To isolate the biological variance from the interfering technical variances in the remaining data, we employed three independent computational methods, Canonical Correlation Analysis (CCA) [19], Surrogate Variable Analysis (SVA) [20] and Mutual Nearest Neighbors (MNN) [21] to systemically correct the potential technical variance (Supplementary Fig. 2A-D). We then quantitatively evaluated the corrected data by an alignment score-based method [19]. The results indicated that the MNN algorithm performed most successfully on eliminating batch

effect in the current dataset (Supplementary Fig. 2E and F). Thus, we proceeded with the MNN-corrected expression matrices for the Seurat pipeline and all subsequent analysis.

A global view was generated to illustrate the landscape of cell composition in UCB. Aside from the NRBC, eleven distinct cell populations were clustered based on their gene expression profiles in both UCB samples. Merged PB dataset were clustered in parallel with UCB cells in the same tSNE space (Fig. 1A). All of the clusters identified were shared by the two UCB samples, demonstrating the robustness of our biological replicate (Supplementary Fig. 2D). Clusters of cells that express known markers of major immune cell types were assigned with their respective identities (Fig. 1B, Supplementary Fig. 3A). The expression pattern of a few representative marker genes was shown as examples (Supplementary Fig. 3B). To further validate the annotations of cell types, we calculated transcriptome-wide correlations between cluster mean expression and previously characterized bulk RNA-seq profiles of sorted immune cell types reported in previous studies [22], which was in concordance with the annotation by canonical markers genes (Supplementary Fig. 4A). Nine major immune cell types and hematopoietic lineages found in PB were identified in UCB, while neutrophil, eosinophil and the bioinformatically excluded NRBC, were only present in the UCB data. The discrepancy of neutrophil and eosinophil is expected due to different cell enrichment approaches used (Methods). The abundance of the common cell types also varied in PB versus that of UCB, suggesting a specific immunological capacity of UCB (Fig. 1C, Supplementary Fig. 4B). We focus the scope of current study

in a few cell types that have profound clinical applications. However, the cellulome landscape of UCB data constitute a rich resource that can be used as a reference to complement transcriptomics analysis performed in bulk or single-cell settings, as well as a guide to future functional studies.

Polarity of cord nucleated red blood cell

In mammal hematopoiesis, NRBCs, or erythroblast, undergo several developmental stages in the bone marrow and progressively decrease cellular volume and RNA content, while accumulating specific functional proteins such as hemoglobin [23, 24]. It has been known for decades that erythroblast exist in relative large numbers in cord blood [25-27]. However, little was known about whether such development processes exist in the cord blood or whether the erythroblast population was homogenous. In our dataset, we found that NRBCs constitute a significant proportion of the total UCB nucleated cells (Supplementary Fig. 4B). Interestingly, the NRBCs in the UCB samples displayed pronounced polarity defined by the divergent expression of a gene repertoire. By ordering NRBCs with differential genes identified within the clusters, we employed Monocle2 software to deduce a pseudotime axis that suggested a gradual change of cellular state [28] (Methods). Evidently, the NRBCs from both UCB samples formed a linear trajectory along the pseudotime axis with no significant branching, indicating that the cell polarity resulted from a continuous changes of gene expression (Fig. 2A). To further validate the dual-polarity of the NRBCs in UCB, we have employed an independently approach to constructed a diffusion pseudotime map

based on the transitions between cells using diffusion-like random walks [29] (Supplementary Fig. 5A). The cell ordering along the trajectories deduced by the two algorithms showed remarkable concordance (Supplementary Fig. 5B).

Next, we modeled gene expression along the Monocle2-inferred trajectory to identify genes characterized by a wave-like pattern. The most prominent of these were the genes encoding surface markers and proteins critical to the function of red blood cells, such as CD47, CD36, hemoglobin and glycophorins [30] (Fig. 2B). The CD47 molecule has long been considered as one of the cell surface markers of primitive erythrocytes [31]. Hemoglobin genes, in contrast, are highly expressed in the relatively mature form of the NRBCs. Thus, the polarity observed most likely reflected the maturity state of the NRBCs. An intermediate cell state that bridges the naïve state (CD47 high) and the mature state (hemoglobin high) was also observed. This intermediate stage was characterized by the elevated expression of a set of genes including those encoding glycophorins (*GYPA* and *GYPB*), suggesting that the cells in this stage exerted a specific function, rather than just transient intermediates. Strikingly, several key transcriptional regulators of erythrocyte homeostasis, including GATA1/2 and BCL11A [32-34], also clearly exhibited divergent patterns along the pseudotime axis (Fig. 2C). GATA1 is a well-characterized transcription factor responsible for the activation of multiple hemoglobin encoding genes in erythroid ontogeny [35], while BCL11A is a transcription factor silencing hemoglobin encoding genes [34]. Another example was CITED2 and SOX6, transcription factors recently characterized as signature molecules specifically expressed in mouse primitive and definitive

erythroblasts, respectively, showed similar specificity in the naïve and intermediate cellular states as defined by the pseudotime axis [36]. In addition, a gradual decrease in the numbers of RNA molecules (represented by UMI) (Fig. 2D) and expressed genes (Fig. 2E) across the pseudotime axis was observed, reflecting the decrease of global gene expression activity due to the NRBC enucleation, supporting the correlation between linear polarity and the cord blood NRBCs maturation. These lines of evidence further corroborated the polarity identified in the NRBC population in UCB, and strongly indicated that the differential activation of transcriptional programs was one of the underlining mechanisms.

Molecular signatures of UCB progenitor cell

A distinct progenitor population was found in the UCB that shared a similar transcriptome profile with the hematopoietic stem cells (HSCs) in the PB dataset (Fig. 1A). However, when the tSNE clustering was performed with the progenitor population in a finer resolution, a secondary subpopulation emerged, demonstrating the heterogeneity of progenitor population in the UCB (Fig. 3A). One subpopulation of UCB progenitor cells overlapped with HSCs in PB and specifically expressed the canonical HSC marker genes such as *CD34*, *SOX4* and *FLT3* (*CD135*) (Fig. 3B, triangles), suggesting their identity as cord blood HSCs. Interestingly, the other subpopulation consists cells only from the UCB (Fig. 3A, dots) and did not express the HSC canonical markers (Fig. 3C, 3D) despite the similarity in overall spectrum of gene expression, which drove the clustered embeddings of these cells in the tSNE space.

Surprisingly, this CD34^{-} UCB specific progenitor population highly expressed the myeloid lineage-specific gene *MS4A3* (Fig. 3D), a known signature of granulocytic-monocytic progenitors (GMPs) [37]. GMPs give rise to mast cell progenitors (MCP) and basophil progenitors (BPC), which are found in the bone marrow, spleen and gastrointestinal mucosa [38]. Furthermore, *FCER1A*, the gene encoding the Fc fragment of the IgE receptor, which is a surface marker frequently used in cell sorting for mast cells [39], was highly expressed in the CD34^{-} cell population; while *CCR3*, a sorting marker for basophils [40, 41], was co-expressed at a comparable level. Similarly, many genes that play regulatory roles in mast cell and basophil differentiation, exemplified by *HDC* and *CSF2RB*, respectively [16, 38, 42], were co-expressed at high level as well (Fig. 3D). The concerted activation of gene repertoires critical in GMP-MCP and GMP-BPC ontogeny axes strongly suggested that these cells were bi-potent progenitors or intermediate cells, similar to the basophil/mast cell progenitor (BMCP) first verified in spleens of adult mice [43]. High level of GATA2 and low level of CEBPA transcription factors were also consistent with the signatures of mouse BMCP [43-45] (Fig. 3D). Such expression signatures is also reminiscent to that of recently identified Basophil/Eosinophil/Mast cell progenitors (Ba/Eo/Ma) in human cord blood and bone marrow [16, 46]. A critical difference between the UCB subpopulation and the mouse BMCP or human Ba/Eo/Ma was that *CD34* expression was turned off, suggesting limited stemness and differentiation commitment in these cells. We thus hypothesized that these cells represent the intermediates before the bifurcation during basophil and mast cell differentiation and termed them umbilical intermediate bi-potent

cells (uIBC). To further explore this hypothesis, we sought to use diffusion maps [29, 47] to characterize the trajectory of the speculated transition from HSC to uIBC. While a gradual identity shifting from HSC to uIBC was observed on the first diffusion component, the trajectory did not show a conclusive bifurcation of uIBC towards the differentiated polarity, likely due to the limited cell abundance (Supplementary Fig. 5C).

Next, we asked whether the switch of cell identities resulted from the alteration of transcriptional programing that governed the differentiation process. Transcription factor enrichment analysis utilizing the Encode [48] and ChEA [49] databases was performed to detect the over-represented combinations of conserved transcription factor binding sites in a given set of genes. The analysis revealed that TAF, YY1 and MYC were the mostly enriched for activating highly expressed genes found in the HSCs as compared to uIBC (Fig. 3E). These transcription factors are well known for their roles in proliferation and cell cycle control [50-53]. Conversely, RUNX1, SPI1 and GATA2 were ranked as the top enriched transcription factors for activating the highly expressed genes in the uIBCs (Fig. 3E). These transcription factors are conventionally considered as master regulators of differentiation of the myeloid lineage [44, 54, 55]. Such functional correlation was further corroborated by the mutually exclusive expression pattern of the top enriched factors. For example, high expression levels of *MYC*, *MAX* and *YY1*, enriched for activating HSC feature genes, were detected in the HSCs; and *vice versa*, high expression levels of *SP1*, *GATA2* and *RUNX1*, were detected in the uIBC (Fig. 3F). These lines of evidence supported that the two subtypes

of cells we found in the progenitor population in UCB were divergent on the hematopoietic axis and may have cord blood-specific functions.

Heterogeneity of cytotoxic innate immune cells

Effective immune response against infection, allergy and cancer generally requires coordinated activation of innate and adaptive immune systems. Recent studies have shown that natural killer T (NKT) cells emerge as a bridge between innate and adaptive immunity to mediate immune responses [56]. In the overall tSNE projection, NK cells were clustered as a contiguous "peninsula" extending from the T cell population (Fig. 1A). Interestingly, *KLRB1*, a lineage marker of NK cells, was expressed in a gradient pattern across the two cell types with no distinct boundary (Supplementary Fig. 6A). Remarkably, the expression of *CD3D/E* was in a reversed gradient with that of *KLRB1* (Supplementary Fig. 6B), as well as those of cytotoxic genes *NKG7*, *PRF1* and *GNLY* (Supplementary Fig. 6C). Such pattern of expression indicated the existence of a group of cells with a bridging identity across the interface, most likely NKT cells. Unlike NK or T cells, NKT cells exhibit distinct tissue specificity under homeostatic conditions, suggesting compartmentalized functions [57-60]. To selectively investigate these cells, we utilized the high-resolution clustering results by Seurat (see Methods), producing more detailed clusters of T and NK cells (Supplementary Fig. 6D), two of the which corresponded to NK cells, the adjacent T cells and the bridging NKT cells that display gradient expression of *CD3D/E* and *KLRB1* (Supplementary Fig. 6E). We next carried out sub-clustering with these cells to

further reveal heterogeneity. By relative expression levels of the lineage markers and the fact that all these cells express a spectrum of cytotoxic marker genes, such as NKG7,PRF1 and GNLY, at high levels (Supplementary Fig. 6A-C), we assigned the cell identity as Cytotoxic T lymphocytes (CTL) ($CD3^{+}KLRB1^{-}$), NK ($CD3^{-}KLRB1^{+}$) and NKT ($CD3^{+}KLRB1^{+}$) [58, 61, 62] (Fig. 4A).

Although CTL, NK and NKT cells were all present in the PB and UCB samples, cell composition was rather different. Apparent heterogeneity was observed in all three cell lineages, and remarkably, represented by the mutually exclusive expression of two granzyme genes, *GZMB* and *GZMK* (Fig. 4B). For example, the NK and CTL cells in PB were each divided into two subgroups, specifically expressing *GZMB* and *GZMK* (Fig. 4C). Similarly, NK and NKT cells in UCB were also sub-grouped into $GZMK^{+}$ and $GZMB^{+}$ populations (Fig. 4D). Thus, based on expression of lineage markers and the two granzyme genes used for this classification scheme (Fig. 4E), total of 6 distinct cell subtypes were defined. All subtypes found in UCB were consistent between donors (Supplementary Fig. 7A), however, both UCB donors lacked $GZMB^{+}$ CTL cells that were present in PB, possibly due to the lack of specific antigen stimulation. It was noteworthy that $GZMB^{+}$ NKT cells were abundantly detected in UCB but missing in PB, begging the question whether this particular subtype possessed specific functions. Collectively, the cell distribution of NKT and CTL indicated that UCB have stronger innate immunity and less adaptive immunity compared to PB. NKT cells were previously reported to have tissue-specific gene expression programs that lead to diverse functions and were termed NKT1, NKT2 and NKT17, predominantly localized

in liver, lung and peripheral lymph node, respectively [58, 63-66]. In our data, the expression profile of the GZMB+ NKT cells was mostly similar to that of the NKT1 type, highlighted by signature expression of *CD44*, *KLRB1*, *ZBTB16*, *IL2RB* and *TBX21* (Supplementary Fig. 7B). But neither GZMB+ or GZMK+ cells expressed *GATA3*, an crucial transcription factor found in NKT2 and NKT17 [67, 68]. Together with the lack of *KLRB1* expression, the GZMK+ NKT cell subtype is distinct from the known NKT2 or NKT17 subtypes [67, 68]. The enriched GZMB+ NKT cells in UCB express a spectrum of chemokines and genes in cytotoxic pathways that may mediate recruitment with other immune cell types to coordinate innate immune response (Fig. 4F). Gene ontology analysis further corroborated that the highly expressed genes of the GZMB+ cells were enriched in innate cytotoxic immunity, such as neutrophil mediated immunity, cellular response to infectious antigens and necrosis factors, while GZMK+ cells in lymphocyte activation, lymphocyte cell-cell adhesion and chemotaxis pathways (Fig. 4G). Thus, we conclude that the cell composition of NKT and other cytotoxic cells varied between PB and UCB.

Unlike NKT, GZMK+ and GZMB+ NK subtypes were both present in PB and UCB (Fig. 4C and D). They may function differently due to their respective granzyme gene activation [69]. Recent studies have shown that orchestrated granzymes expression is part of the functional program that enable cytotoxic cells to exert specific functions [70, 71]. As exemplified by the NK subtypes, *GZMB* and *GZMK* expression represents such functional diversity and highlighted their respective cytotoxic gene expression programs. To reveal the elements of these two programs, we systemically

compared the GZMB$^+$ subtypes of NK, NKT and CTL cells found in PB or UCB by testing the co-occurrence of signature genes that were specific to each subtype (see Methods). As a result, amongst the four sets of signature genes ranging from 116 to 144 in number, 31 signature genes were shared by all four subtypes (Fig. 5A). Similarly, 22 signature genes were found common in the corresponding GZMK$^+$ subtypes (Fig. 5B). Permutation tests were performed to estimate the significance of the four-way intersection in both cases and the resulted p values were both $< 3x10^{-16}$. These two sets of signature genes (31 and 22) that we found were defined as GZMB and GZMK co-expressed genes, respectively, that were likely to contribute to the elimination of specific antigens. To corroborate the findings, we calculated the Pearson's correlation of cell-averaged expression of all 53 genes in GZMB$^+$ and GZMK$^+$ subtypes of NK and NKT cells in UCB and CTL and NK cells in PB. As expected, unsupervised clustering revealed two major modules, corresponding to the GZMB and GZMK programs (Fig. 5C and D). Interestingly, within each program a smaller core module was discovered, highlighted by *EEF1A1*, *TPT1*, *COTL1* and *LTB* in the GZMK program; and *FGFBP2*, *PRF1*, *GZMA*, *FCGR3A* and *CCL4* in the GZMB program (Fig. 5C, red labeled genes). Similar analysis was performed in the PB cells, and we found the core modules largely consistent with that in UCB, though the GZMK core module was less prominent (Fig. 5D, red labeled genes). These enriched genes in the two programs that we identified represent common features of the GZMB$^+$ and GZMK$^+$ subtypes of cytotoxic cells. They may serve as specific selection markers and targets for perturbation in further functional studies.

DISCUSSION

For the first time, we present here a single-cell level transcriptomic landscape of nucleated cells in UCB. By analyzing the expression pattern of known marker genes, we identified UCB cells belonging to almost all of the major hematopoietic lineages in PB, covering lymphoid, myeloid and hematopoietic progenitor cells. We also observed that certain cell populations were highly enriched in UCB cells, such as NRBCs, uIBCs and GZMB$^+$ NKT cells. The features we discovered regarding these cells were consistent in both UCB donors. However, it is important to keep in mind that the UCB donors' shared factors, such as genetic background, could contribute to the enrichment of these UCB-specific cell subtypes. A related technical challenge in the current study that we encountered was the severe batch effect among sample types and donors. To minimize the technical variance that could lead to misinterpretation of the data, we rigorously tested three widely used algorithms for batch effect correction, namely, CCA, SVA and MNN. Based on a quantitative evaluation of cell segregation in the tSNE space, performance of MNN and CCA appeared comparable and effective for our datasets, though MNN scored marginally higher.

In adults, red blood cells are generated mainly in the bone marrow from nucleated cells identified as erythroid precursors. These cells undergo morphological changes through cell divisions and gradual decrease in cell size and RNA species, increase in chromatin condensation and hemoglobin protein accumulation. Such changes have been associated with the early stages of maturation of red blood cell. In

our dataset we also observed such a dynamic cellular state in a linear polarity. While it is possible that the erythroid precursors at different stages in UCB may be migrated from the bone marrow, our finding also suggested the possibility that the erythroid precursors may undergo a similar maturation process in the UCB.

Progenitor cell populations in UCB also appeared to be a mixture of at least two distinct subpopulations. It is conceivable that the HSC subpopulation ($CD34^{+}$) we identified may be a mixture of hematopoietic stem cells and various early multipotent progenitors committed to differentiation, which was termed primed progenitors and extensively discussed in a recent study profiling UCB HSC at single-cell level [16]. Due to the lack of CD34 enrichment, the UCB data in current study have too few HSCs to recapitulate the heterogeneity reported in this study. The uIBC, a unique UCB subpopulation not seen in PB, were identified with characteristics of both basophil and mast cell signatures. A similar bipotent population (BMCP) exists in mouse spleen and is capable of divergent development [43]. Signature gene expression, including transcription factors and surface markers were remarkably similar between BMCP and uIBC, except that uIBC lack the expression of the conventional progenitor marker CD34. Although uIBC and HSC in UCB were globally similar in their transcriptomic profiles, the lack of CD34 made it difficult to conclude whether these uIBCs were indeed progenitors or transient intermediates captured during UCB hematopoiesis. The functional implication of their existence points to the development process downstream of Ba/Eo/Ma primed branch detected in the previous study [16], specifically, when the Ba/Eo/Ma primed cells lose stemness markers (i.e. CD34) and further express lineage

genes. Functional validations are necessary to determine the potential abilities of self-renewal and lineage regeneration of these cells and substantiate the similarity with mouse BMCP or Ba/Eo/Ma primed cells at functional level.

Next, we interrogated the UCB single-cell data at a finer scale and discovered unreported heterogeneity amongst CTL, NK and NKT cells in UCB that appeared in different composition and granzyme expression pattern as those in PB. It is noteworthy that mutually exclusive pattern between GZMA/B/perforin program versus GZMK program was a common feature in cytotoxic cell lineages in UCB and PB. This finding is consistent with the previous studies performed in PB [69], demonstrating that human granzymes are differentially expressed in distinct sub-populations that may have function outside of orchestrating cytotoxicity. Interestingly, a previously unknown NKT population that may be unique to UCB was identified as $GZMB^{+}$ NKT cells that do not express *GZMK* but highly express *GZMA*, *GZMH*, and *PRF1* genes, suggesting the activation of specific cytotoxicity mediated by granzyme and perforin pathways. NKT cells have an essential role in bridging innate and adaptive immunity against infectious diseases and tumorigenesis, thus they possess significant therapeutic values. UCB transplants have demonstrated remarkable effectiveness in treating many types of blood cancers. Adoptive transfer of the NKT cells has been tested in animal models [72, 73], and several clinical trials are in process to test the safety and efficiency of NKT cell transfer to harness the solid tumors in human [74-77]. The enhanced understanding of the NKT cell heterogeneity in UCB would benefit our selection of appropriate source and the activation of the cytotoxicity of NKT cells to target cancer and other diseases.

Therefore, we speculated that a targeted enrichment, modulation or engineering of the existing NKT populations in the UCB could lead to considerable improvement in the efficacy of enhancing protective immune responses.

Taken together, our data provides the first single-cell transcriptomic references for UCB, which could be used as a standard dataset for comparative analysis. We expect that this dataset will prove useful in uncovering the novel molecular signatures that define the cellular heterogeneity in UCB and provide markers for targeted enrichment of certain cell types of interest to researchers in multiple fields. Our dataset is a rich resource to formulate hypothesis of signaling pathway activation, transcription control and other mechanistic studies in the field of functional immunology at single cell level.

METHODS

Sample collection

Two umbilical cord blood samples were collected from healthy donors immediately after caesarean section with informed consents. Samples were stored in EDTA anticoagulant tubes and transported to laboratory within 1 hour. $CD45^+$ and $CD45^-$ cells were isolated from 1 mL cord blood by positive and negative selection, respectively, using Whole Blood CD45 MicroBeads (Miltenyi,130-090-872) and Whole Blood Column Kit (Miltenyi, 130-093-545). Next, the $CD45^+$ and $CD45^-$ cells were counted by hemocytometer and mixed at the ratio of 4 to 1. The cells were further gently pipetted into a single-cell suspension and diluted to concentration of 700 cell/μL. The public single cell gene expression dataset of peripheral blood mononuclear cells (PB1 and PB2) were generated in sample from a single donor. PB1 and PB2 in the current study correspond to Cell Ranger 2.0.1 processed "8k PBMCs from a Healthy Donor" and "4k PBMCs from a Healthy Donor", respectively, under the URL: https://support.10xgenomics.com/single-cell-gene-expression/datasets.

UCB library construction and sequencing

Single-cell suspension of UCB samples was loaded to Single-cell 3'Chips (10× Genomics, USA) and subjected to GemCode Single-Cell Instrument (10× Genomics, USA) to generate single-cell Gel Beads in Emulsion (GEMs), per manufacture's instruction. GEMs were next subjected to library construction by Chromium™ Single-cell 3' Reagent Kits v2 (10× Genomics, USA), steps of which included RT incubation,

cDNA amplification, fragmentation, end repair, A-tailing, adaptor ligation, and sample index PCR. However, such library was originally designed to be sequenced by the Illumina sequencing platform. In order to convert the libraries to that compatible with BGISEQ-500 sequencer, we performed a 12-cycle PCR on the libraries with BGISEQ adaptor primers, and subsequent DNA circularization, rolling-cycle amplification (RCA) to generate DNA Nano Balls (DNBs). The purified DNBs were sequenced by BGISEQ-500 sequencer, generating reads containing 16 bp of 10X™ Barcodes, 10 bp of unique molecular indices (UMI) and 100 bp of 3' cDNA sequences. Each library was sequenced in three lanes, yielding ~1.9 billion reads in total.

Alignment and initial processing of sequencing data

CellRanger toolkit (10X Genomics, USA, version 2.0.0) was employed to align the cDNA reads to GRCh38 transcriptome. Filtered UMI expression matrices of both samples were generated with the default parameters and an additional "--force-cells=4000" parameter [78]. The expression matrices of all samples were first normalized by "cellranger aggr" function in the CellRanger toolkit, with the parameter "--normalize=mapped". As a result, raw expression data of total ~32,000 single cells of UCB sample was generated.

Quality filtration of cells

In accordance with the published pipelines and quality control standard [18], abnormal cells in all datasets were uniformly filtered out based on their gene expression

distribution. A cell was considered as abnormal if any of the following criterion was met: (1) detected gene number is below 400; (2) detected gene number is higher than 2,000, 2,000, 3,500 and 3,000 for PB1, PB2, UCB1 and UCB2 datasets, respectively; (3) more than 8%, 8%, 6% and 7% of detected genes are mitochondria genes in PB1, PB2, UCB1 and UCB2 datasets, respectively. Detected gene is defined as any gene that expresses in at least 30 individual cells at level of UMI $\geq$ 1 in any given dataset. Total of 8,380, 3,977, 8,981 and 9,638 cells remained after the filtering in PB1, PB2, UCB1 and UCB2 datasets, respectively.

Cells clustering in individual UCB samples

Next, the filtered expression matrices of UCB1 and UCB2 were used for unsupervised cell-clustering by the Seurat package (2.3.4), adopting the typical pipeline that was recommended by the authors [18]. Total of 3,113 (UCB1) and 2,409 (UCB2) variable genes were used for "RunPCA" function. Subsequently, the top 10 PCs were subjected to "FindClusters" and "RunTSNE" function with high resolution setting at 2.0 (Supplementary Fig. 1A). In the dimensional reduced tSNE space, the clusters of NRBCs were identified on the basis of the concerted expression of hemoglobin genes, such as *HBG1* and *HBM* (Supplementary Fig. 1B). Then we bioinformatically isolated the total of 672 NRBCs from UCB1 and UCB2 as a sub-dataset for further analyses. The NRBC-excluded data were then subjected to merging and batch effect removal. The reason we excluded NRBC prior to data merging was that we noticed that the massively expressed hemoglobin genes significantly interfered the merging of UCB

cells with PB cells and cell clustering, yielding highly sample-segregated cell embeddings in the tSNE space, regardless of batch-removal methods or parameters used.

Batch effects correction

Strong technical bias introduced by sample preparation, library construction and/or sequencing was observed in the merged data (Supplementary Fig. 2A). To evaluate the available strategy for batch correction, we independently tested SVA, CCA and MNN and compared their outcome. For SVA method, we first log transformed the expression values (as in log(exp + 1)), then used the ComBat function in the SVA package to minimize batch effects with the default parameters [20]. For CCA, we performed Canonical Correlation Analysis in Seurat package to correct batch effects. We tested different parameters when processed CCA analysis, and observed best performance while chose 15 canonical vectors and 1,500 shared high variable genes.

For MNN, we first created a SingleCellExperiment object to store the counts and metadata together for each sample, using SingleCellExperiment package (1.3.10). These cells were pre-clustered by quickCluster function. Size factors was computed for the endogenous genes using the deconvolution method by computeSumFactors function [79]. We then acquired the normalized log-expression values and distinguished highly variable genes by trendVar function and decomposed the gene-specific variance into biological and technical components by decomposeVar function. To obtain a single set of features for batch correction, we computed the average

biological component across all 4 batches. All genes with positive biological components were retained to ensure that biological variance was preserved. All batches were rescaled to account for differences in sequencing depth by multiBatchNorm function. Lastly, fastMNN function was applied to the four samples, using the retained genes with parameters k=50, d=50, approximate=TRUE, auto.order=TRUE. In the end, corrected expression values for 3,570 highly variable genes was generated by tcrossprod function, and these expression values were used in downstream cell clustering and pseudotime analysis.

Evaluation of batch correction

The alignment scores of the methods above were calculated based on tSNE plots according to the strategy of previously study [19]. First, neutrophil and eosinophil that were only present in UCB datasets were masked from the datasets. Then, we randomly sampled cells from the four datasets with same number of cells and constructed a nearest-neighbor graph based on their relative positions in tSNE space. For each sampled cell, we calculated the cell numbers from the dataset sample in the *k* nearest-neighbors and average with total cells to obtain $\overline{x}$. The alignment score was then calculated as following:

$$\text{Alignment Score} = 1 - \frac{\overline{x}-\frac{k}{N}}{k-\frac{k}{N}}$$

The alignment scores were normalized by size of the datasets and scaled to range from 0 to 1. For Supplementary Figure 2E, the parameters used were *k* = 800, N = 4. As shown, alignment score of MNN was marginally higher than that of CCA. To

rule out the potential bias from the arbitrary selection of *k*, we tested different *k* from 100 to 1,000, and observed that the high scores by MNN was independent of *k* selection (Supplementary Fig. 2F).

Cell type annotation

After batch-correction by MNN, the merged expression matrix was further filtered following the typical Seurat pipeline. Specifically, ribosomal genes were removed and cells with mitochondria gene UMI percentage high than 10%, and cells with more than 11,000 total UMI counts were removed. Then the expression matrix was normalized by NormalizeData function. The corrected expression matrix was used to perform dimensionality reduction following the typical Seurat pipeline. Next, 3,556 variable genes in the batch-corrected expression matrix were used for RunPCA, ProjectPCA, FindClusters and RunTSNE functions with default parameters, except dims.use = 1:13 and resolution = 2.

Subsequently, the feature genes for each cluster were identified using normalized data by the Seurat FindAllMarkers function with parameter min.pct = 0.25, thresh.use = 0.25. Four minor clusters with ~5% (same as estimated by 10X Genomics, USA) of total cells were suspected as doublets as they share feature genes from two adjacent large clusters were removed from the datasets. The identity of each cell cluster was manually annotated by the specific expression of commonly known markers. Unsupervised annotation by comparing averaged single cell expression levels with bulk RNA-seq data of sorted immune cells was also performed to validate the results as

previously described [80]. Pearson's correlation was used to calculate the distance between the cell-averaged feature gene expression with the corresponding levels in bulk RNA-seq data (Supplementary Fig. 4A).

Pseudotime analysis of NRBC

Total of 672 NRBCs identified by the individually clustered UCB datasets were directly merged for the following analysis. After removing five abnormal cells on account of their significantly deviated mitochondrial gene expression level (>2.5 %), 667 nucleated red blood cells used to infer the developmental polarity of NRBCs. NRBCs were ordered according to the pseudotime deduced by 1,859 ordering genes excluding ribosomal protein transcripts, that are differentially expressed (FDR < 0.05) by "clusterCells" function in Monocle2 package (version 2.6.4). In parallel, pseudotemporal trajectory was deduced by diffusion map API in Scanpy package (python 3.6.6, scanpy 1.3.2), using default parameters (n_neighbors=20 and n_pcs=5 for preprocessing.neighbors function; and n_comps=15 for tools.diffmap function).

Diffusion map algorithm generated pseudotemporal ordering of cells were compared with that of Monocle2 using Spearman's rank-order correlation (Supplementary Fig. 5B).

Clustering and pseudotime analysis of UCB progenitor cells

UCB progenitor cells were re-clustered using Seurat packages, same as in the global clustering described above. In order to visualize the potential transition of cell

identities from HSC to uIBC, we used diffusion map API in Scanpy package to calculate the diffusion pseudotime trajectory with default parameters similarly as in NRBCs analysis, with exception of n_pcs=6. Then, we used FindAllMarkers function in Seurat package with parameters min.pct=0.3 to find feature genes within the two clusters. In order to identify the divergent transcription factor programs in the two groups of cells, a web-based tool "Enrichr" (http://amp.pharm.mssm.edu/Enrichr/) was employed to analyze the enrichment of transcription factor binding on the signature genes set of each progenitor cell group [81].

Cytotoxic cell clustering and profiling

Cytotoxic cells of interest were selected by unsupervised clustering at resolution=2 by the FindClusters function in Seurat package (Supplementary Fig. 6D). The two clusters (highlighted in Supplementary Fig. 6E) covering the gradient expression of multiple cytotoxic genes in Supplementary Fig. 6C were selected to create 2 new sub-datasets, according to their respective sample type. Then the 2 sets of the UMI matrices with 2,271 cells in PB and 879 cells in UCB were subjected to a typical Seurat pipeline. Sequential application of Seurat functions NormalizeData, RunPCA, ProjectPCA, FindClusters and RunTSNEfunctions with parameter dims.use = 1:3, resolution = 1.5 for UCB and dims.use = 1:8, resolution = 1.5 for PB were performed. Subsequently, the cluster-specific genes used to annotate cell subtypes were identified using normalized data by the Seurat FindAllMarkers function with parameter min.pct = 0.25, thresh.use = 0.25.

Signature gene selection in GZMK$^+$ and GZMB$^+$ subtypes

To identify the common features of the GZMK and GZMB programs in the cytotoxic cells (Fig. 5), the GZMB/GZMK expressing NK, NKT and CTL subtypes were used to create a new Seurat object by SubsetData function. The function FindAllMarkers was used to identify corresponding features genes of each clusters with parameter min.pct = 0.25, thresh.use = 0.25.

The four-way Venn diagrams of feature genes shown in Fig.5A and B were generated using R package VennDiagram. To verify the statistical significance of the enrichment of the four-way-overlapped genes (GZMB/GZMK program genes), One Sample t-test was carried out by testing the mean number of overlapping genes from randomly sampled pools of genes, sizes of which was kept the same as the original feature genes in the four subtypes. Co-expression modules in Figure 5C and D were identified by unsupervised clustering of Pearson's correlation of cell-averaged expression values.

Gene ontology analysis of UCB GZMB$^+$ NTK cells

To deduce the potential functions of the signature genes in UCB GZMB$^+$ NTK cells, gene ontology enrichment analysis was performed by clusterProfiler package (v3.8.1) using the top 100 feature genes of GZMK$^+$ NKT cells in UCB identified by Seurat package. Then we simplified the output from enrichGO by removing redundancy of enriched GO terms using simplify function.

AUTHOR CONTRIBUTION

X.LIU., Y.H. and S.L. jointly supervised research. Y.Z., B.L. and G.Y. designed the experiments. X.W., K.G., Y.Z. and X.Z. performed the experiments. Y.Z. and Jingwan WANG. pre-processed the sequencing data. Y.Z., X.LI., J.W., Z.W. and Jingwan WANG. analyzed the data. W.ZHAO and B.F. collected the cord blood. X.LI. and Y.Z. wrote the manuscript. X.LIU, Q.W., B.C., H.Y., F.C., Jian WANG., W.ZHANG, X.X. and F.X. revised the manuscript. All authors have reviewed and approved the final manuscript.

ACKNOWLEDGMENTS

We thank the two donors who generously provided the UCB samples. We also thank Liqin Xu, Zhikun Zhao for helpful discussions and BGI colleagues who have helped producing the high-quality data. This work was supported by Shenzhen Municipal Government of China (JCYJ20170817145404433 and JCYJ20170817145428361)

ETHICS, CONSENT AND PERMISSIONS

This study was approved by ethic committee of Shenzhen Second People’s hospital and BGI (BGI-IRB 18120). Written informed consents were obtained from both donors who donated the samples.

COMPETING INTERESTS

The authors declare no competing financial interests.

REFERENCES

1. Kurtzberg J. Update on umbilical cord blood transplantation. Current opinion in pediatrics. 2009;21 1:22-9.
2. Paloczi K. Immunophenotypic and functional characterization of human umbilical cord blood mononuclear cells. Leukemia. 1999;13 Suppl 1:S87-9.
3. Park SK and Won JH. Usefulness of umbilical cord blood cells in era of hematopoiesis research. International journal of stem cells. 2009;2 2:90-6.
4. Rocha V, Wagner JE, Jr., Sobocinski KA, Klein JP, Zhang MJ, Horowitz MM, et al. Graft-versus-host disease in children who have received a cord-blood or bone marrow transplant from an HLA-identical sibling. Eurocord and International Bone Marrow Transplant Registry Working Committee on Alternative Donor and Stem Cell Sources. The New England journal of medicine. 2000;342 25:1846-54. doi:10.1056/NEJM200006223422501.
5. Laughlin MJ, Barker J, Bambach B, Koc ON, Rizzieri DA, Wagner JE, et al. Hematopoietic engraftment and survival in adult recipients of umbilical-cord blood from unrelated donors. The New England journal of medicine. 2001;344 24:1815-22. doi:10.1056/NEJM200106143442402.
6. Migliaccio AR, Adamson JW, Stevens CE, Dobrila NL, Carrier CM and Rubinstein P. Cell dose and speed of engraftment in placental/umbilical cord blood transplantation: graft progenitor cell content is a better predictor than nucleated cell quantity. Blood. 2000;96 8:2717-22.
7. Navin NE. The first five years of single-cell cancer genomics and beyond. Genome Res. 2015;25 10:1499-507. doi:10.1101/gr.191098.115.
8. Tanay A and Regev A. Scaling single-cell genomics from phenomenology to mechanism. Nature. 2017;541 7637:331-8. doi:10.1038/nature21350.
9. Eltahla AA, Rizzetto S, Pirozyan MR, Betz-Stablein BD, Venturi V, Kedzierska K, et al. Linking the T cell receptor to the single cell transcriptome in antigen-specific human T cells. Immunology and cell biology. 2016;94 6:604-11. doi:10.1038/icb.2016.16.
10. Proserpio V and Mahata B. Single-cell technologies to study the immune system. Immunology. 2016;147 2:133-40. doi:10.1111/imm.12553.
11. Zheng C, Zheng L, Yoo JK, Guo H, Zhang Y, Guo X, et al. Landscape of Infiltrating T Cells in Liver Cancer Revealed by Single-Cell Sequencing. Cell. 2017;169 7:1342-56 e16. doi:10.1016/j.cell.2017.05.035.
12. Gaublomme JT, Yosef N, Lee Y, Gertner RS, Yang LV, Wu C, et al. Single-Cell Genomics Unveils Critical Regulators of Th17 Cell Pathogenicity. Cell. 2015;163 6:1400-12. doi:10.1016/j.cell.2015.11.009.
13. Patil VS, Madrigal A, Schmiedel BJ, Clarke J, O'Rourke P, de Silva AD, et al. Precursors of human CD4(+) cytotoxic T lymphocytes identified by single-cell transcriptome analysis. Science immunology. 2018;3 19 doi:10.1126/sciimmunol.aan8664.
14. Paul F, Arkin Y, Giladi A, Jaitin DA, Kenigsberg E, Keren-Shaul H, et al. Transcriptional Heterogeneity and Lineage Commitment in Myeloid Progenitors. Cell. 2015;163 7:1663-77. doi:10.1016/j.cell.2015.11.013.
15. Velten L, Haas SF, Raffel S, Blaszkiewicz S, Islam S, Hennig BP, et al. Human haematopoietic stem cell lineage commitment is a continuous process. Nat Cell Biol. 2017;19

4:271-81. doi:10.1038/ncb3493.
16. Zheng S, Papalexi E, Butler A, Stephenson W and Satija R. Molecular transitions in early progenitors during human cord blood hematopoiesis. Mol Syst Biol. 2018;14 3:e8041. doi:10.15252/msb.20178041.
17. Tusi BK, Wolock SL, Weinreb C, Hwang Y, Hidalgo D, Zilionis R, et al. Population snapshots predict early haematopoietic and erythroid hierarchies. Nature. 2018;555 7694:54-60. doi:10.1038/nature25741.
18. Satija R, Farrell JA, Gennert D, Schier AF and Regev A. Spatial reconstruction of single-cell gene expression data. Nat Biotechnol. 2015;33 5:495-502. doi:10.1038/nbt.3192.
19. Butler A, Hoffman P, Smibert P, Papalexi E and Satija R. Integrating single-cell transcriptomic data across different conditions, technologies, and species. Nat Biotechnol. 2018;36 5:411-20. doi:10.1038/nbt.4096.
20. Leek JT, Johnson WE, Parker HS, Jaffe AE and Storey JD. The sva package for removing batch effects and other unwanted variation in high-throughput experiments. Bioinformatics. 2012;28 6:882-3. doi:10.1093/bioinformatics/bts034.
21. Haghverdi L, Lun ATL, Morgan MD and Marioni JC. Batch effects in single-cell RNA-sequencing data are corrected by matching mutual nearest neighbors. Nat Biotechnol. 2018;36 5:421-7. doi:10.1038/nbt.4091.
22. Novershtern N, Subramanian A, Lawton LN, Mak RH, Haining WN, McConkey ME, et al. Densely interconnected transcriptional circuits control cell states in human hematopoiesis. Cell. 2011;144 2:296-309. doi:10.1016/j.cell.2011.01.004.
23. Migliaccio AR. Erythroblast enucleation. Haematologica. 2010;95 12:1985-8. doi:10.3324/haematol.2010.033225.
24. Ji P, Murata-Hori M and Lodish HF. Formation of mammalian erythrocytes: chromatin condensation and enucleation. Trends Cell Biol. 2011;21 7:409-15. doi:10.1016/j.tcb.2011.04.003.
25. Hebbar S, Misha M and Rai L. Significance of maternal and cord blood nucleated red blood cell count in pregnancies complicated by preeclampsia. Journal of pregnancy. 2014;2014:496416. doi:10.1155/2014/496416.
26. Hermansen MC. Nucleated red blood cells in the fetus and newborn. Archives of disease in childhood Fetal and neonatal edition. 2001;84 3:F211-5.
27. Merenstein GB, Blackmon LR and Kushner J. Nucleated red-cells in the newborn. Lancet. 1970;1 7659:1293-4.
28. Qiu X, Hill A, Packer J, Lin D, Ma YA and Trapnell C. Single-cell mRNA quantification and differential analysis with Census. Nat Methods. 2017;14 3:309-15. doi:10.1038/nmeth.4150.
29. Haghverdi L, Buttner M, Wolf FA, Buettner F and Theis FJ. Diffusion pseudotime robustly reconstructs lineage branching. Nat Methods. 2016;13 10:845-8. doi:10.1038/nmeth.3971.
30. van Schravendijk MR, Handunnetti SM, Barnwell JW and Howard RJ. Normal human erythrocytes express CD36, an adhesion molecule of monocytes, platelets, and endothelial cells. Blood. 1992;80 8:2105-14.
31. Oldenborg PA, Zheleznyak A, Fang YF, Lagenaur CF, Gresham HD and Lindberg FP. Role of CD47 as a marker of self on red blood cells. Science. 2000;288 5473:2051-4.
32. Dore LC and Crispino JD. Transcription factor networks in erythroid cell and megakaryocyte development. Blood. 2011;118 2:231-9. doi:10.1182/blood-2011-04-285981.

33. Bresnick EH, Hewitt KJ, Mehta C, Keles S, Paulson RF and Johnson KD. Mechanisms of erythrocyte development and regeneration: implications for regenerative medicine and beyond. Development. 2018;145 1 doi:10.1242/dev.151423.
34. Liu N, Hargreaves VV, Zhu Q, Kurland JV, Hong J, Kim W, et al. Direct Promoter Repression by BCL11A Controls the Fetal to Adult Hemoglobin Switch. Cell. 2018;173 2:430-42 e17. doi:10.1016/j.cell.2018.03.016.
35. Ohneda K and Yamamoto M. Roles of hematopoietic transcription factors GATA-1 and GATA-2 in the development of red blood cell lineage. Acta haematologica. 2002;108 4:237-45. doi:10.1159/000065660.
36. Kingsley PD, Greenfest-Allen E, Frame JM, Bushnell TP, Malik J, McGrath KE, et al. Ontogeny of erythroid gene expression. Blood. 2013;121 6:e5-e13. doi:10.1182/blood-2012-04-422394.
37. Ishibashi T, Yokota T, Satoh Y, Ichii M, Sudo T, Doi Y, et al. Identification of MS4A3 as a reliable marker for early myeloid differentiation in human hematopoiesis. Biochemical and biophysical research communications. 2018;495 3:2338-43. doi:10.1016/j.bbrc.2017.12.117.
38. Iwasaki H and Akashi K. Myeloid lineage commitment from the hematopoietic stem cell. Immunity. 2007;26 6:726-40. doi:10.1016/j.immuni.2007.06.004.
39. Stone KD, Prussin C and Metcalfe DD. IgE, mast cells, basophils, and eosinophils. The Journal of allergy and clinical immunology. 2010;125 2 Suppl 2:S73-80. doi:10.1016/j.jaci.2009.11.017.
40. Hausmann OV, Gentinetta T, Fux M, Ducrest S, Pichler WJ and Dahinden CA. Robust expression of CCR3 as a single basophil selection marker in flow cytometry. Allergy. 2011;66 1:85-91. doi:10.1111/j.1398-9995.2010.02431.x.
41. Chirumbolo S, Ortolani R and Vella A. CCR3 as a single selection marker compared to CD123/HLADR to isolate basophils in flow cytometry: some comments. Cytometry Part A : the journal of the International Society for Analytical Cytology. 2011;79 2:102-6. doi:10.1002/cyto.a.21008.
42. Gurish MF, Tao H, Abonia JP, Arya A, Friend DS, Parker CM, et al. Intestinal mast cell progenitors require CD49dbeta7 (alpha4beta7 integrin) for tissue-specific homing. The Journal of experimental medicine. 2001;194 9:1243-52.
43. Arinobu Y, Iwasaki H, Gurish MF, Mizuno S, Shigematsu H, Ozawa H, et al. Developmental checkpoints of the basophil/mast cell lineages in adult murine hematopoiesis. Proc Natl Acad Sci U S A. 2005;102 50:18105-10. doi:10.1073/pnas.0509148102.
44. Iwasaki H, Mizuno S, Arinobu Y, Ozawa H, Mori Y, Shigematsu H, et al. The order of expression of transcription factors directs hierarchical specification of hematopoietic lineages. Genes Dev. 2006;20 21:3010-21. doi:10.1101/gad.1493506.
45. Iwasaki H, Mizuno S, Mayfield R, Shigematsu H, Arinobu Y, Seed B, et al. Identification of eosinophil lineage-committed progenitors in the murine bone marrow. The Journal of experimental medicine. 2005;201 12:1891-7. doi:10.1084/jem.20050548.
46. Dahlin JS, Malinovschi A, Ohrvik H, Sandelin M, Janson C, Alving K, et al. Lin- CD34hi CD117int/hi FcepsilonRI+ cells in human blood constitute a rare population of mast cell progenitors. Blood. 2016;127 4:383-91. doi:10.1182/blood-2015-06-650648.
47. Coifman RR, Lafon S, Lee AB, Maggioni M, Nadler B, Warner F, et al. Geometric diffusions as a tool for harmonic analysis and structure definition of data: diffusion maps. Proc Natl Acad

Sci U S A. 2005;102 21:7426-31. doi:10.1073/pnas.0500334102.

48. Consortium EP. An integrated encyclopedia of DNA elements in the human genome. Nature. 2012;489 7414:57-74. doi:10.1038/nature11247.

49. Lachmann A, Xu H, Krishnan J, Berger SI, Mazloom AR and Ma'ayan A. ChEA: transcription factor regulation inferred from integrating genome-wide ChIP-X experiments. Bioinformatics. 2010;26 19:2438-44. doi:10.1093/bioinformatics/btq466.

50. Trop-Steinberg S and Azar Y. Is Myc an Important Biomarker? Myc Expression in Immune Disorders and Cancer. The American journal of the medical sciences. 2018;355 1:67-75. doi:10.1016/j.amjms.2017.06.007.

51. Amati B, Littlewood TD, Evan GI and Land H. The c-Myc protein induces cell cycle progression and apoptosis through dimerization with Max. EMBO J. 1993;12 13:5083-7.

52. Lin CY, Tuan J, Scalia P, Bui T and Comai L. The cell cycle regulatory factor TAF1 stimulates ribosomal DNA transcription by binding to the activator UBF. Curr Biol. 2002;12 24:2142-6.

53. Lu Z, Hong CC, Kong G, Assumpcao A, Ong IM, Bresnick EH, et al. Polycomb Group Protein YY1 Is an Essential Regulator of Hematopoietic Stem Cell Quiescence. Cell Rep. 2018;22 6:1545-59. doi:10.1016/j.celrep.2018.01.026.

54. North TE, Stacy T, Matheny CJ, Speck NA and de Bruijn MF. Runx1 is expressed in adult mouse hematopoietic stem cells and differentiating myeloid and lymphoid cells, but not in maturing erythroid cells. Stem cells. 2004;22 2:158-68. doi:10.1634/stemcells.22-2-158.

55. Dakic A, Metcalf D, Di Rago L, Mifsud S, Wu L and Nutt SL. PU.1 regulates the commitment of adult hematopoietic progenitors and restricts granulopoiesis. The Journal of experimental medicine. 2005;201 9:1487-502. doi:10.1084/jem.20050075.

56. Van Kaer L, Parekh VV and Wu L. Invariant natural killer T cells: bridging innate and adaptive immunity. Cell and tissue research. 2011;343 1:43-55. doi:10.1007/s00441-010-1023-3.

57. Bendelac A, Savage PB and Teyton L. The biology of NKT cells. Annual review of immunology. 2007;25:297-336. doi:10.1146/annurev.immunol.25.022106.141711.

58. Godfrey DI, MacDonald HR, Kronenberg M, Smyth MJ and Van Kaer L. NKT cells: what's in a name? Nature reviews Immunology. 2004;4 3:231-7. doi:10.1038/nri1309.

59. Kronenberg M. Toward an understanding of NKT cell biology: progress and paradoxes. Annual review of immunology. 2005;23:877-900. doi:10.1146/annurev.immunol.23.021704.115742.

60. Van Kaer L. NKT cells: T lymphocytes with innate effector functions. Current opinion in immunology. 2007;19 3:354-64. doi:10.1016/j.coi.2007.03.001.

61. Van Der Vliet HJ, Nishi N, Koezuka Y, Peyrat MA, Von Blomberg BM, Van Den Eertwegh AJ, et al. Effects of alpha-galactosylceramide (KRN7000), interleukin-12 and interleukin-7 on phenotype and cytokine profile of human Valpha24+ Vbeta11+ T cells. Immunology. 1999;98 4:557-63.

62. Vivier E and Anfossi N. Inhibitory NK-cell receptors on T cells: witness of the past, actors of the future. Nature reviews Immunology. 2004;4 3:190-8. doi:10.1038/nri1306.

63. Gumperz JE, Miyake S, Yamamura T and Brenner MB. Functionally distinct subsets of CD1d-restricted natural killer T cells revealed by CD1d tetramer staining. The Journal of experimental medicine. 2002;195 5:625-36.

64. Lee PT, Benlagha K, Teyton L and Bendelac A. Distinct functional lineages of human

V(alpha)24 natural killer T cells. The Journal of experimental medicine. 2002;195 5:637-41.
65. Coquet JM, Chakravarti S, Kyparissoudis K, McNab FW, Pitt LA, McKenzie BS, et al. Diverse cytokine production by NKT cell subsets and identification of an IL-17-producing CD4-NK1.1- NKT cell population. Proc Natl Acad Sci U S A. 2008;105 32:11287-92. doi:10.1073/pnas.0801631105.
66. Michel ML, Keller AC, Paget C, Fujio M, Trottein F, Savage PB, et al. Identification of an IL-17-producing NK1.1(neg) iNKT cell population involved in airway neutrophilia. The Journal of experimental medicine. 2007;204 5:995-1001. doi:10.1084/jem.20061551.
67. Brennan PJ, Brigl M and Brenner MB. Invariant natural killer T cells: an innate activation scheme linked to diverse effector functions. Nature reviews Immunology. 2013;13 2:101-17. doi:10.1038/nri3369.
68. Constantinides MG and Bendelac A. Transcriptional regulation of the NKT cell lineage. Current opinion in immunology. 2013;25 2:161-7. doi:10.1016/j.coi.2013.01.003.
69. Bade B, Boettcher HE, Lohrmann J, Hink-Schauer C, Bratke K, Jenne DE, et al. Differential expression of the granzymes A, K and M and perforin in human peripheral blood lymphocytes. Int Immunol. 2005;17 11:1419-28. doi:10.1093/intimm/dxh320.
70. Bengsch B, Ohtani T, Herati RS, Bovenschen N, Chang KM and Wherry EJ. Deep immune profiling by mass cytometry links human T and NK cell differentiation and cytotoxic molecule expression patterns. J Immunol Methods. 2018;453:3-10. doi:10.1016/j.jim.2017.03.009.
71. Kiniry BE, Hunt PW, Hecht FM, Somsouk M, Deeks SG and Shacklett BL. Differential Expression of CD8(+) T Cell Cytotoxic Effector Molecules in Blood and Gastrointestinal Mucosa in HIV-1 Infection. J Immunol. 2018;200 5:1876-88. doi:10.4049/jimmunol.1701532.
72. Lam PY, Nissen MD and Mattarollo SR. Invariant Natural Killer T Cells in Immune Regulation of Blood Cancers: Harnessing Their Potential in Immunotherapies. Front Immunol. 2017;8:1355. doi:10.3389/fimmu.2017.01355.
73. Bagnara D, Ibatici A, Corselli M, Sessarego N, Tenca C, De Santanna A, et al. Adoptive immunotherapy mediated by ex vivo expanded natural killer T cells against CD1d-expressing lymphoid neoplasms. Haematologica. 2009;94 7:967-74. doi:10.3324/haematol.2008.001339.
74. Exley MA, Friedlander P, Alatrakchi N, Vriend L, Yue S, Sasada T, et al. Adoptive Transfer of Invariant NKT Cells as Immunotherapy for Advanced Melanoma: A Phase I Clinical Trial. Clinical cancer research : an official journal of the American Association for Cancer Research. 2017;23 14:3510-9. doi:10.1158/1078-0432.CCR-16-0600.
75. Motohashi S, Ishikawa A, Ishikawa E, Otsuji M, Iizasa T, Hanaoka H, et al. A phase I study of in vitro expanded natural killer T cells in patients with advanced and recurrent non-small cell lung cancer. Clinical cancer research : an official journal of the American Association for Cancer Research. 2006;12 20 Pt 1:6079-86. doi:10.1158/1078-0432.CCR-06-0114.
76. Kunii N, Horiguchi S, Motohashi S, Yamamoto H, Ueno N, Yamamoto S, et al. Combination therapy of in vitro-expanded natural killer T cells and alpha-galactosylceramide-pulsed antigen-presenting cells in patients with recurrent head and neck carcinoma. Cancer science. 2009;100 6:1092-8. doi:10.1111/j.1349-7006.2009.01135.x.
77. Yamasaki K, Horiguchi S, Kurosaki M, Kunii N, Nagato K, Hanaoka H, et al. Induction of NKT cell-specific immune responses in cancer tissues after NKT cell-targeted adoptive immunotherapy. Clinical immunology. 2011;138 3:255-65. doi:10.1016/j.clim.2010.11.014.
78. Zheng GX, Terry JM, Belgrader P, Ryvkin P, Bent ZW, Wilson R, et al. Massively parallel

digital transcriptional profiling of single cells. Nat Commun. 2017;8:14049. doi:10.1038/ncomms14049.

79. Lun AT, Bach K and Marioni JC. Pooling across cells to normalize single-cell RNA sequencing data with many zero counts. Genome Biol. 2016;17:75. doi:10.1186/s13059-016-0947-7.

80. Azizi E, Carr AJ, Plitas G, Cornish AE, Konopacki C, Prabhakaran S, et al. Single-Cell Map of Diverse Immune Phenotypes in the Breast Tumor Microenvironment. Cell. 2018;174 5:1293-308 e36. doi:10.1016/j.cell.2018.05.060.

81. Chen EY, Tan CM, Kou Y, Duan Q, Wang Z, Meirelles GV, et al. Enrichr: interactive and collaborative HTML5 gene list enrichment analysis tool. BMC Bioinformatics. 2013;14:128. doi:10.1186/1471-2105-14-128.

FIGURE LEGEND

Figure 1: Cell types identified in the umbilical cord blood

A. Global t-distributed stochastic neighbor embedding (tSNE) plots of merged UCB and PB cells. Cell clusters are colored to indicate cell types by expressed known markers. UCB cells are colorized in the left panel and PB in the right. Cell type and respective colors are labeled on the right.

B. Heatmap of scaled average gene expression of the major canonical markers (columns) detected in different cell types in merged cells of UCB and PB (rows).

C. Distribution of each cell abundance in each cell type of the PB and UCB datasets.

Figure 2: Polarity of NRBCs in the UCB samples

A. The ordering of NRBCs along pseudotime in a two-dimensional space determined by Monocle2. Each dot represents a single NRBC. Color gradient represents the pseudotemporal order in the upper panel. Cells from the two UCB samples are labeled in the same topology in the bottom panel.

B. Heatmap of gene expression in NRBCs ordered by pseudotime (x-axis). Three clusters of pseudotime-dependent genes are grouped into primitive stage (top), intermediate stage (middle) and mature stage (bottom).

C. Heatmap of key transcription factor expression similar to B.

D. Numbers of detected UMI in each NRBC ordered by pseudotime. Each dot represents a NRBC, and the color represents the corresponding UCB sample of each cell.

E. Numbers of detected gene in each NRBC ordered by pseudotime. Each dot represents a NRBC, and the color represents the corresponding UCB sample of each cell.

Figure 3: Heterogeneous molecular signatures of progenitor cells in UCB

A. The re-clustered tSNE projection of progenitor cells from UCB and PB samples. The samples are labeled with different colors for each cell.

B. The two cell clusters, HSC and uIBC, are represented by triangles and dots, respectively. The color gradient represents the pseudotemporal order.

C. Heatmap of differentially expressed signature genes in the progenitors. Cells along the x-axis were ordered the same as tSNE 1 axis in A. The color bar on top denotes the HSC and uIBC clusters as well as the corresponding samples.

D. Violin plots of exemplary feature gene expression of the HSC and uIBC cells. Blue: uIBC and red: HSC.

E. Transcription factor enrichment analysis of the HSC and uIBC cells using the HSC signature genes (1,012 genes, top left) and the uIBC signature genes (106 genes, bottom left) revealed enriched transcription factors in HSC (top middle) and uIBC (bottom middle). The bargraphs of corresponding enrichment scores (-log FDR) are shown on the right.

F. Violin plots of exemplary enriched transcription factor expression in the HSC and uIBC cells. Blue: uIBC and red: HSC.

Figure 4: Heterogeneity of cytotoxic cells in PB and UCB

A. tSNE plots of re-clustered cytotoxic cells from the PB (left) and UCB (right) datasets. Each dot represents a single cytotoxic cell. Color demonstrates the expression of CD3D and KLRB1. Yellow: CD3D highly expressed cells, blue: KLRB1 highly expressed cell, red: cells highly express both CD3D and KLRB1, grey: cells express neither genes.

B. The same tSNE plots as in A, and the color demonstrates the expression of GZMB and GZMK in a similar color scheme.

C. tSNE plots of cytotoxic cells from the PB datasets. Cell subtypes ($GZMK^+$ CTL, $GZMB^+$ CTL, $GZMK^+$ NKT, $GZMB^+$ NK, $GZMK^+$ NK) are labeled with different colors.

D. tSNE plots of cytotoxic cells from the UCB datasets. Cell subtypes ($GZMK^+$ CTL, $GZMK^+$ NKT, $GZMB^+$ NKT, $GZMB^+$ NK, $GZMK^+$ NK) are labeled with different colors.

E. Violin plots of signature gene expression among the subtypes in UCB (right) and PB (left). Coloring is consistent with that in C.

F. Heatmap of exemplary differentially expressed signature genes in the GZMB$^+$ NKT and GZMK$^+$ NKT subtypes. The color bar on top denotes the GZMB$^+$ NKT and GZMK$^+$ NKT subtypes.

G. Gene ontology (GO) analysis of differentially expressed signature genes that specific to GZMB$^+$ NKT (upper panel) and those specific to GZMB$^+$ NKT (bottom panel) subtypes in UCB. The most enriched GO terms are ordered on the y-axis. X-axis represents the gene percentage in the enriched GO terms. The sizes of dots represent the number of genes included in each GO term. The color gradient of dots represents the adjusted p-values of each enriched GO term.

Figure 5: Enrichment of feature genes of granzyme B and K subtypes

A. Four-way Venn diagrams reveal the enrichment of the feature genes among the GZMB positive cell types.

B. Four-way Venn diagrams reveal the enrichment of the feature genes among the GZMK positive cell types.

C. Pearson's correlation of expression of the four-way-overlapped gene in A and B in UCB datasets.

D. Pearson's correlation of expression of the four-way-overlapped gene in A and B in PB datasets.

Supplementary Figure 1: Pre-clustering of UCB samples and exclusion of NRBCs

A. Pre-clustering of cells in UCB1 (left) and UCB2 (right). Each dot represents a single cell, and cells are color-labeled by cluster in the tSNE space.

B. tSNE plots of the normalized expression of hemoglobin genes *HBG1* (left) and *HBM* (right) in UCB1 (up) and UCB2 (bottom). The color gradient represents expression level.

Supplementary Figure 2: Sample distribution and evaluation of batch-correction methods

A. Sample distribution in the tSNE space without any batch-removal processing. Cells are color-labeled by sample.

B-D. Sample distribution in tSNE space after CCA (B), Combat (C) and MNN process (D). Cells are color-labeled by sample in the same way as in A.

E. Bargraph of alignment scores produced by different methods as shown in A-D.

F. Comparison of alignment scores between CCA and MNN with different parameters (from k=100 to k=1000).

Supplementary Figure 3: Signature gene expression of each cell types

A. Heatmap of scaled average gene expression of the signature genes (column) detected in different cell types in UCB and PB (rows).

B. tSNE plots of the normalized expression of marker genes in the same global topology as in Fig. 1A. Each dot represents a single cell, and the color gradient represents the normalized gene expression.

Supplementary Figure 4: Cell type annotation composition.

A. Pearson's correlation between the cell-averaged feature gene expression with the corresponding levels in bulk RNA-seq data generated in sorted cells.

B. Table of cell numbers of different cell types each sample.

Supplementary Figure 5: Pseudotime analysis in NRBCs and Progenitor cells

A. The ordering of NRBCs along pseudotime in a two-dimensional space determined by diffusion map. Each dot represents a single NRBC. Color gradient represents the pseudotemporal order in the left panel. Cells from the two UCB samples are labeled in the same topology in the right panel.

B. Correlation between pseudotemporal ordering of cells by Monocle2 and diffusion map. Correlation coefficient was calculated by Spearman's rank testing.

C. The ordering of progenitor cells along pseudotime in a two-dimensional space determined by diffusion map. Each dot represents a single cell, and the color gradient represents the order of pseudotime (left). The sample distribution along the pseudotime, and the color represents the corresponding sample (right).

Supplementary Figure 6: Cytotoxic signature gene expression in NK and NKT populations

A-C. Zoom-in tSNE plots of the normalized expression of cytotoxicity and related genes of the cytotoxic cell. Each dot represents a single cell, and the color gradient represents the normalized gene expression.

D. Unsupervised high-resolution clustering of merged PB and UCB cells in the same tSNE topology as in Fig.1A. Clusters are labeled by different colors.

E. Similar as D, cells with cytotoxic features that are further analyzed are highlighted in blue color.

Supplementary Figure 7: Differential gene expression in NK and NKT subpopulations

A. Cells are color-labeled by samples in the same tSNE space as in Fig. 4C and Fig. 4D. Each dot represents a single cell in PB (left) and UCB (right).

B. Violin plots show the scaled expression of indicated differential genes between $GZMB^{+}$ NKT and $GZMK^{+}$ NKT subsets in UCB.

Figure 1

Figure

Click here to download Figure UCB Fig181119.pdf

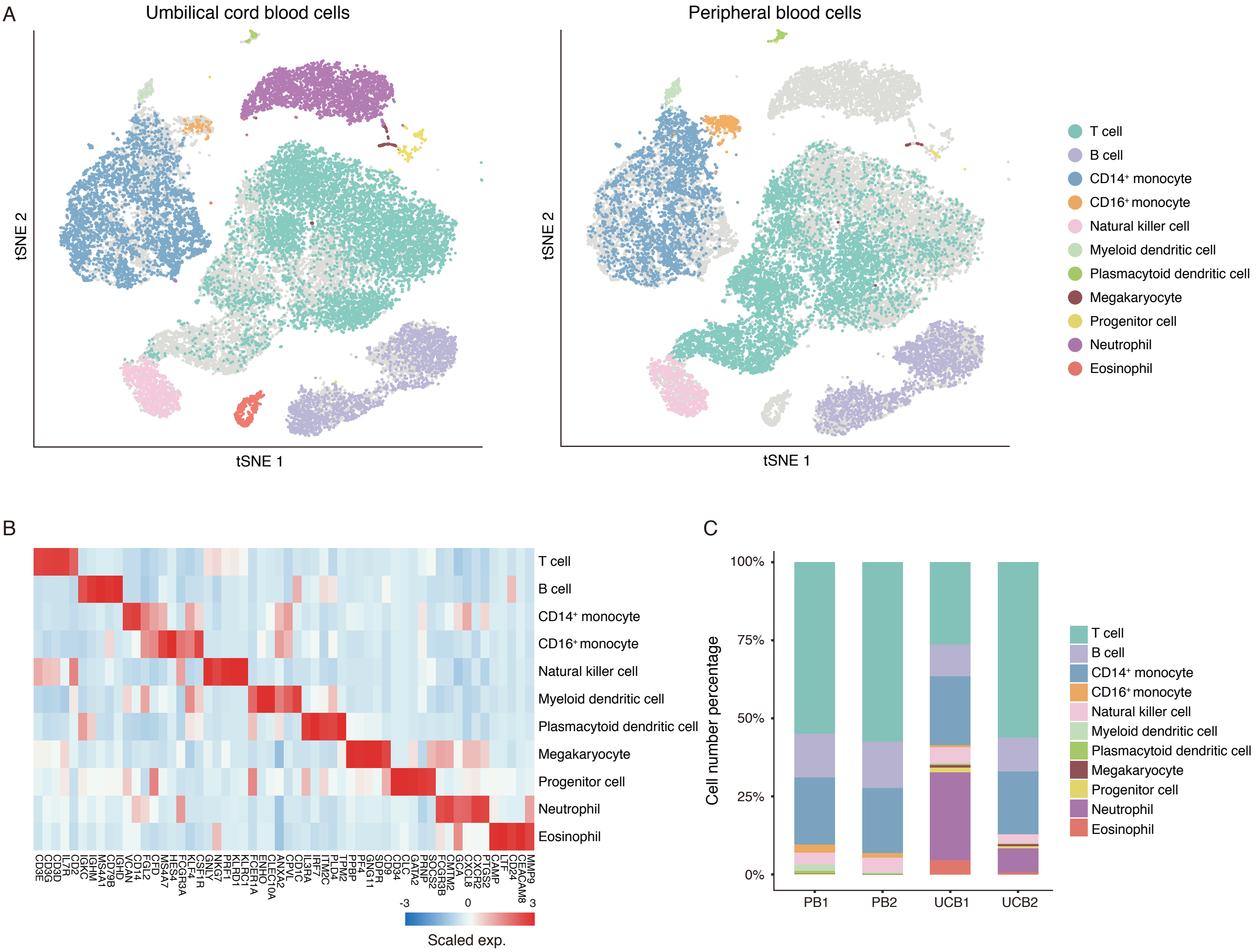

Figure 2

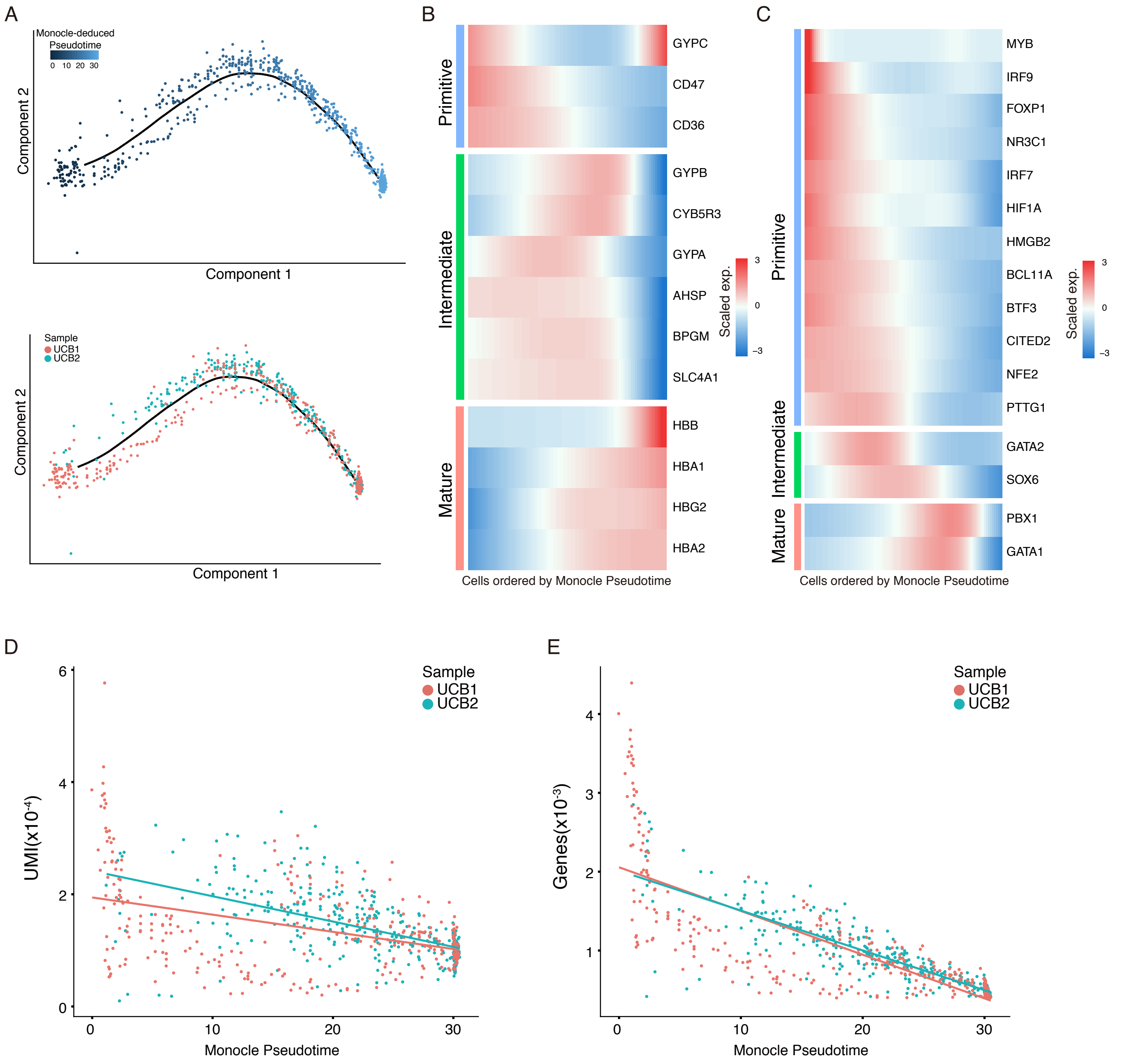
A
Monocle-deduced
Pseudotime
0 10 20 30
Component 2
Component 1
Sample
UCB1
UCB2
Component 2
Component 1
B
Primitive
Intermediate
Mature
GYPC
CD47
CD36
GYPB
CYB5R3
GYPA
AHSP
BPGM
SLC4A1
HBB
HBA1
HBG2
HBA2
Scaled exp.
3
0
−3
Cells ordered by Monocle Pseudotime
C
Primitive
Intermediate
Mature
MYB
IRF9
FOXP1
NR3C1
IRF7
HIF1A
HMGB2
BCL11A
BTF3
CITED2
NFE2
PTTG1
GATA2
SOX6
PBX1
GATA1
Scaled exp.
3
0
−3
Cells ordered by Monocle Pseudotime
D
UMI(x10^-4)
0
2
4
6
0
10
20
30
Monocle Pseudotime
Sample
UCB1
UCB2
E
Genes(x10^-3)
1
2
3
4
0
10
20
30
Monocle Pseudotime
Sample
UCB1
UCB2

Figure 3

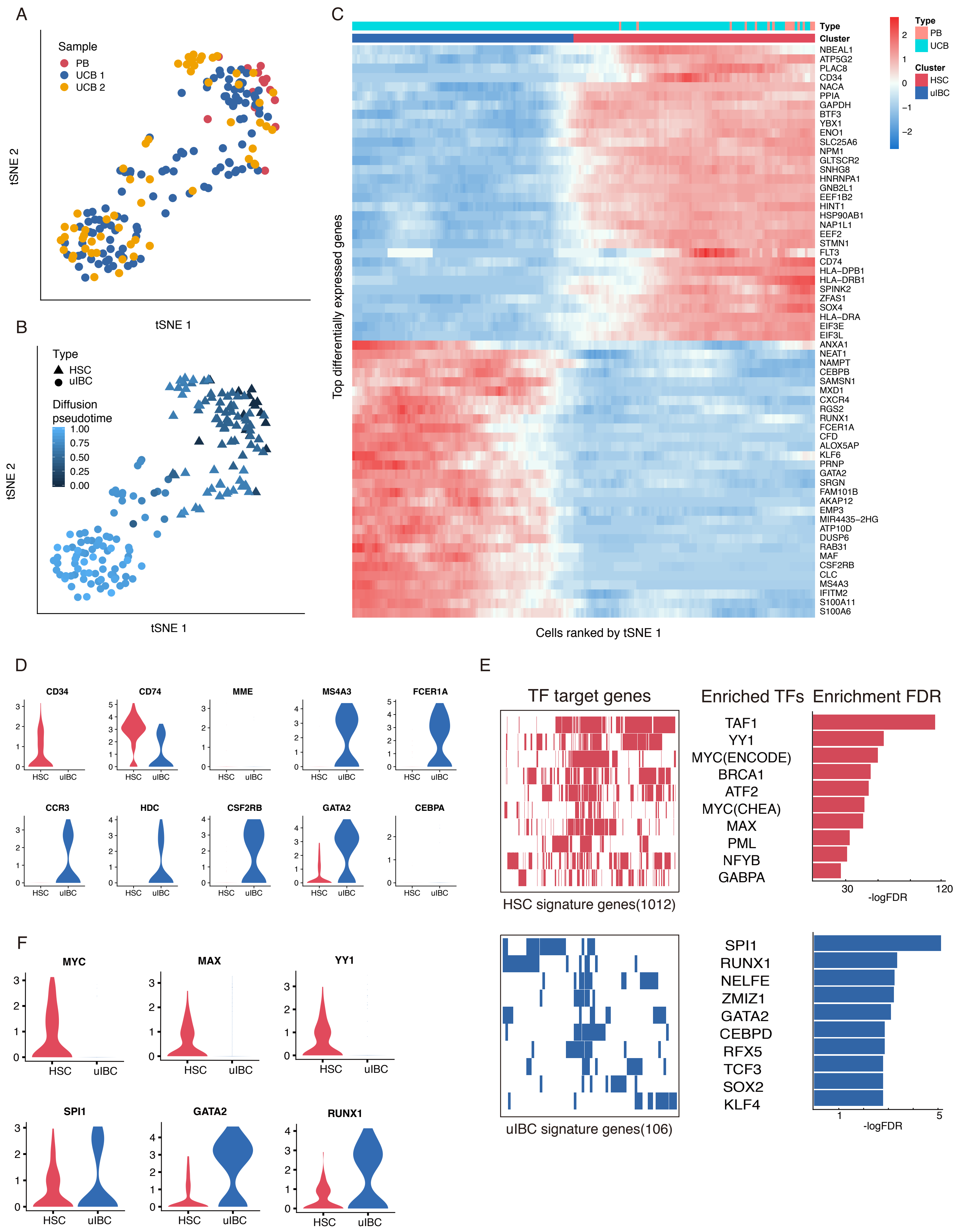
A
Sample
PB
UCB 1
UCB 2
tSNE 1
tSNE 2
B
Type
HSC
uIBC
Diffusion pseudotime
1.00
0.75
0.50
0.25
0.00
tSNE 1
tSNE 2
C
Type
Cluster
PB
UCB
HSC
uIBC
Top differentially expressed genes
NBEAL1
ATP5G2
PLAC8
CD34
NACA
PPIA
GAPDH
BTF3
YBX1
ENO1
SLC25A6
NPM1
GLTSCR2
SNHG8
HNRNPA1
GNB2L1
EEF1B2
HINT1
HSP90AB1
NAP1L1
EEF2
STMN1
FLT3
CD74
HLA–DPB1
HLA–DRB1
SPINK2
ZFAS1
SOX4
HLA–DRA
EIF3E
EIF3L
ANXA1
NEAT1
NAMPT
CEBPB
SAMSN1
MXD1
CXCR4
RGS2
RUNX1
FCER1A
CFD
ALOX5AP
KLF6
PRNP
GATA2
SRGN
FAM101B
AKAP12
EMP3
MIR4435–2HG
ATP10D
DUSP6
RAB31
MAF
CSF2RB
CLC
MS4A3
IFITM2
S100A11
S100A6
Cells ranked by tSNE 1
D
CD34
CD74
MME
MS4A3
FCER1A
CCR3
HDC
CSF2RB
GATA2
CEBPA
HSC
uIBC
E
TF target genes
Enriched TFs
Enrichment FDR
TAF1
YY1
MYC(ENCODE)
BRCA1
ATF2
MYC(CHEA)
MAX
PML
NFYB
GABPA
30
120
-logFDR
HSC signature genes(1012)
SPI1
RUNX1
NELFE
ZMIZ1
GATA2
CEBPD
RFX5
TCF3
SOX2
KLF4
1
5
-logFDR
uIBC signature genes(106)
F
MYC
MAX
YY1
SPI1
GATA2
RUNX1
HSC
uIBC

Figure 4

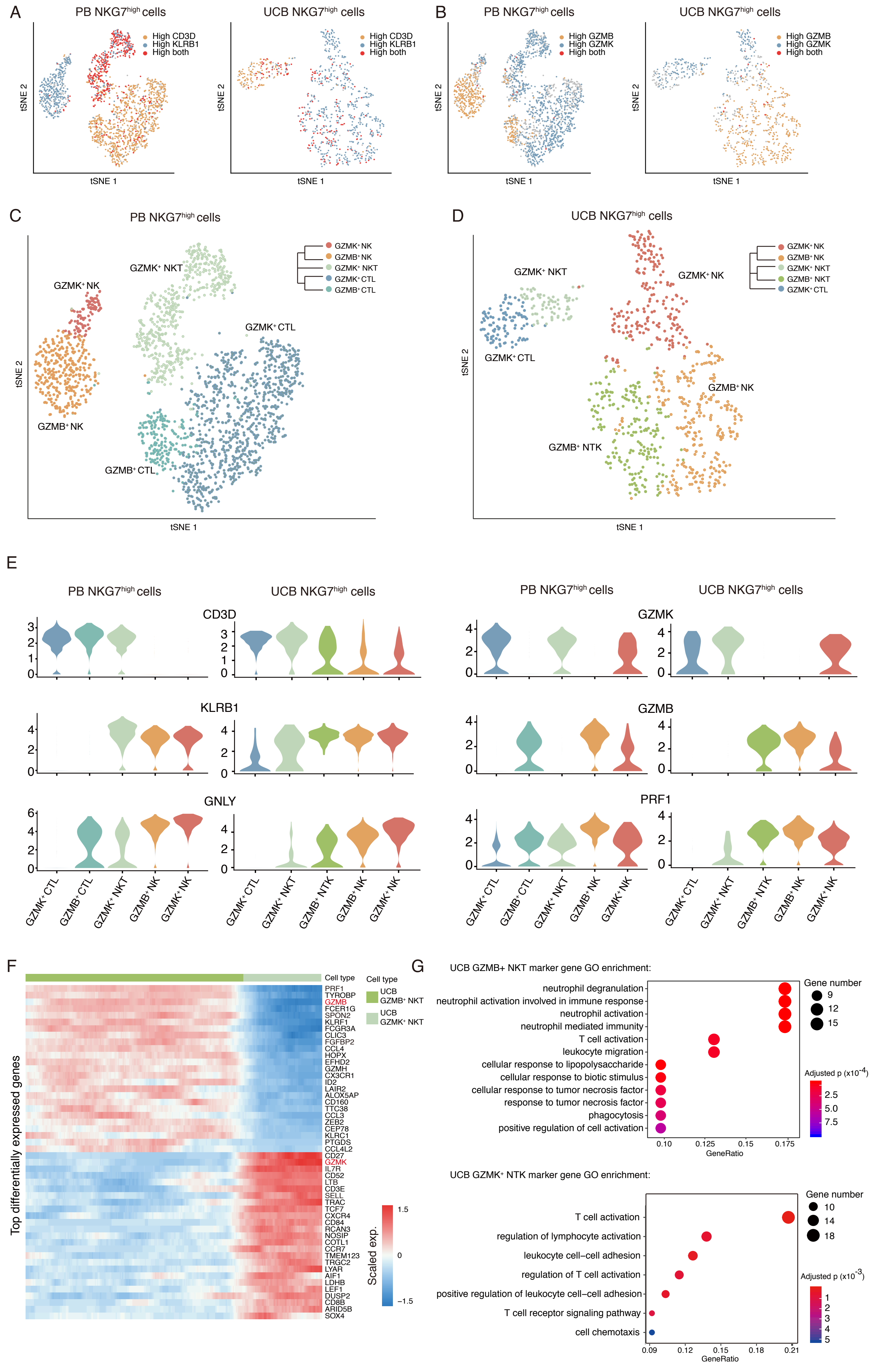

A
PB NKG7high cells
UCB NKG7high cells
High CD3D
High KLRB1
High both
tSNE 1
tSNE 2
B
High GZMB
High GZMK
C
PB NKG7high cells
GZMK+ NK
GZMB+ NK
GZMK+ NKT
GZMK+ CTL
GZMB+ CTL
D
UCB NKG7high cells
GZMB+ NKT
GZMB+ NTK
E
CD3D
KLRB1
GNLY
GZMK
GZMB
PRF1
F
Cell type
UCB GZMB+ NKT
UCB GZMK+ NKT
Top differentially expressed genes
Scaled exp.
PRF1
TYROBP
GZMB
FCER1G
SPON2
KLRF1
FCGR3A
CLIC3
FGFBP2
CCL4
HOPX
EFHD2
GZMH
CX3CR1
ID2
LAIR2
ALOX5AP
CD160
TTC38
CCL3
ZEB2
CEP78
KLRC1
PTGDS
CCL4L2
CD27
GZMK
IL7R
CD52
LTB
CD3E
SELL
TRAC
TCF7
CXCR4
CD84
RCAN3
NOSIP
COTL1
CCR7
TMEM123
TRGC2
LYAR
AIF1
LDHB
LEF1
DUSP2
CD8B
ARID5B
SOX4
G
UCB GZMB+ NKT marker gene GO enrichment:
neutrophil degranulation
neutrophil activation involved in immune response
neutrophil activation
neutrophil mediated immunity
T cell activation
leukocyte migration
cellular response to lipopolysaccharide
cellular response to biotic stimulus
cellular response to tumor necrosis factor
response to tumor necrosis factor
phagocytosis
positive regulation of cell activation
Gene number
Adjusted p (x10-4)
GeneRatio
UCB GZMK+ NTK marker gene GO enrichment:
T cell activation
regulation of lymphocyte activation
leukocyte cell−cell adhesion
regulation of T cell activation
positive regulation of leukocyte cell−cell adhesion
T cell receptor signaling pathway
cell chemotaxis
Adjusted p (x10-3)

Figure 5

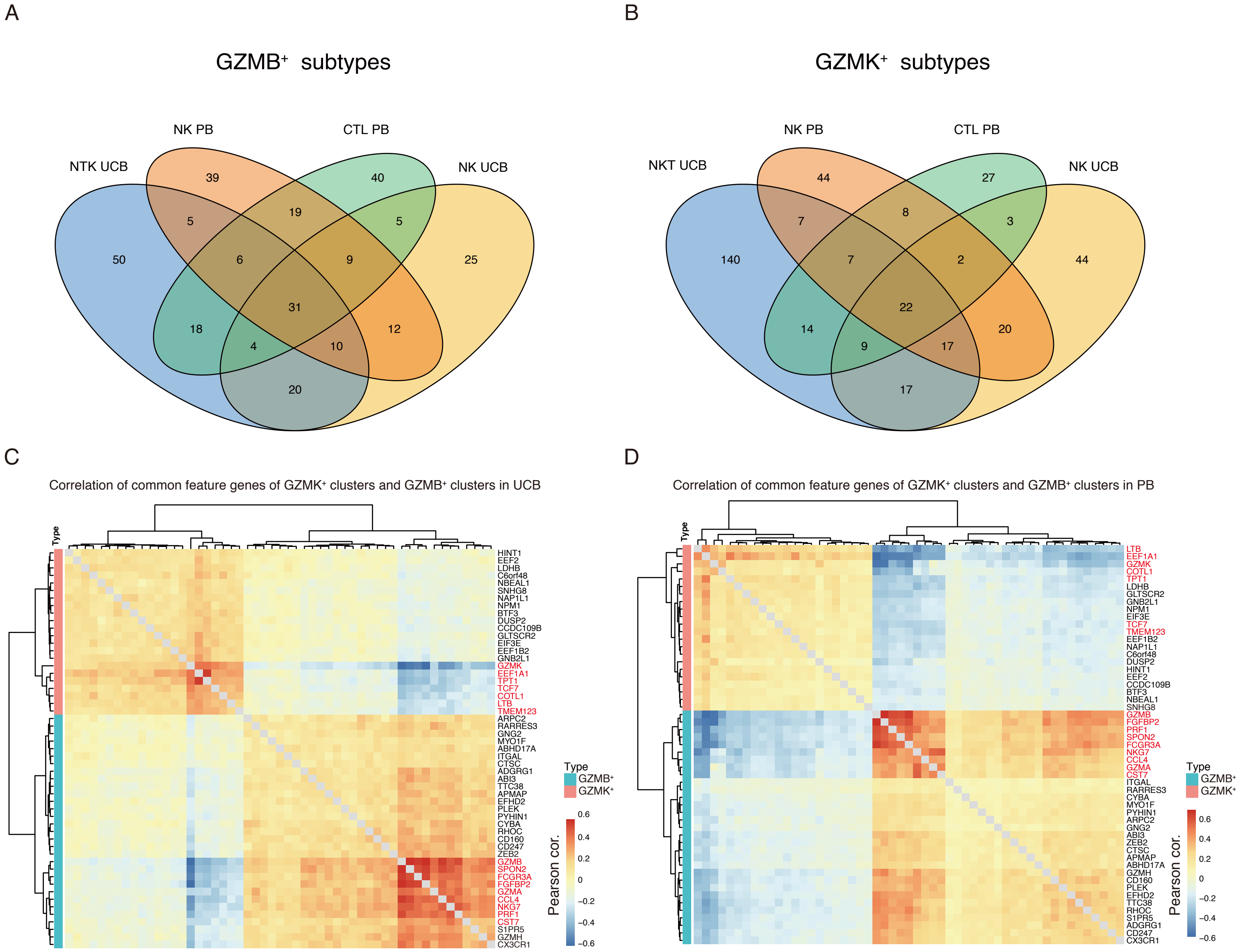
A
GZMB⁺ subtypes
NTK UCB
NK PB
CTL PB
NK UCB
39
40
50
5
19
5
25
6
9
31
18
12
4
10
20
B
GZMK⁺ subtypes
NKT UCB
NK PB
CTL PB
NK UCB
44
27
140
7
8
3
44
7
2
22
14
20
9
17
17
C
Correlation of common feature genes of GZMK⁺ clusters and GZMB⁺ clusters in UCB
Type
HINT1
EEF2
LDHB
C6orf48
NBEAL1
SNHG8
NAP1L1
NPM1
BTF3
DUSP2
CCDC109B
GLTSCR2
EIF3E
EEF1B2
GNB2L1
GZMK
EEF1A1
TPT1
TCF7
COTL1
LTB
TMEM123
ARPC2
RARRES3
GNG2
MYO1F
ABHD17A
ITGAL
CTSC
ADGRG1
ABI3
TTC38
APMAP
EFHD2
PLEK
PYHIN1
CYBA
RHOC
CD160
CD247
ZEB2
GZMB
SPON2
FCGR3A
FGFBP2
GZMA
CCL4
NKG7
PRF1
CST7
S1PR5
GZMH
CX3CR1
Type
GZMB⁺
GZMK⁺
Pearson cor.
0.6
0.4
0.2
0
−0.2
−0.4
−0.6
D
Correlation of common feature genes of GZMK⁺ clusters and GZMB⁺ clusters in PB
Type
LTB
EEF1A1
GZMK
COTL1
TPT1
LDHB
GLTSCR2
GNB2L1
NPM1
EIF3E
TCF7
TMEM123
EEF1B2
NAP1L1
C6orf48
DUSP2
HINT1
EEF2
CCDC109B
BTF3
NBEAL1
SNHG8
GZMB
FGFBP2
PRF1
SPON2
FCGR3A
NKG7
CCL4
GZMA
CST7
ITGAL
RARRES3
CYBA
MYO1F
PYHIN1
ARPC2
GNG2
ABI3
ZEB2
CTSC
APMAP
ABHD17A
GZMH
CD160
PLEK
EFHD2
TTC38
RHOC
S1PR5
ADGRG1
CD247
CX3CR1
Type
GZMB⁺
GZMK⁺
Pearson cor.
0.6
0.4
0.2
0
−0.2
−0.4
−0.6

Supplementary Figure 1

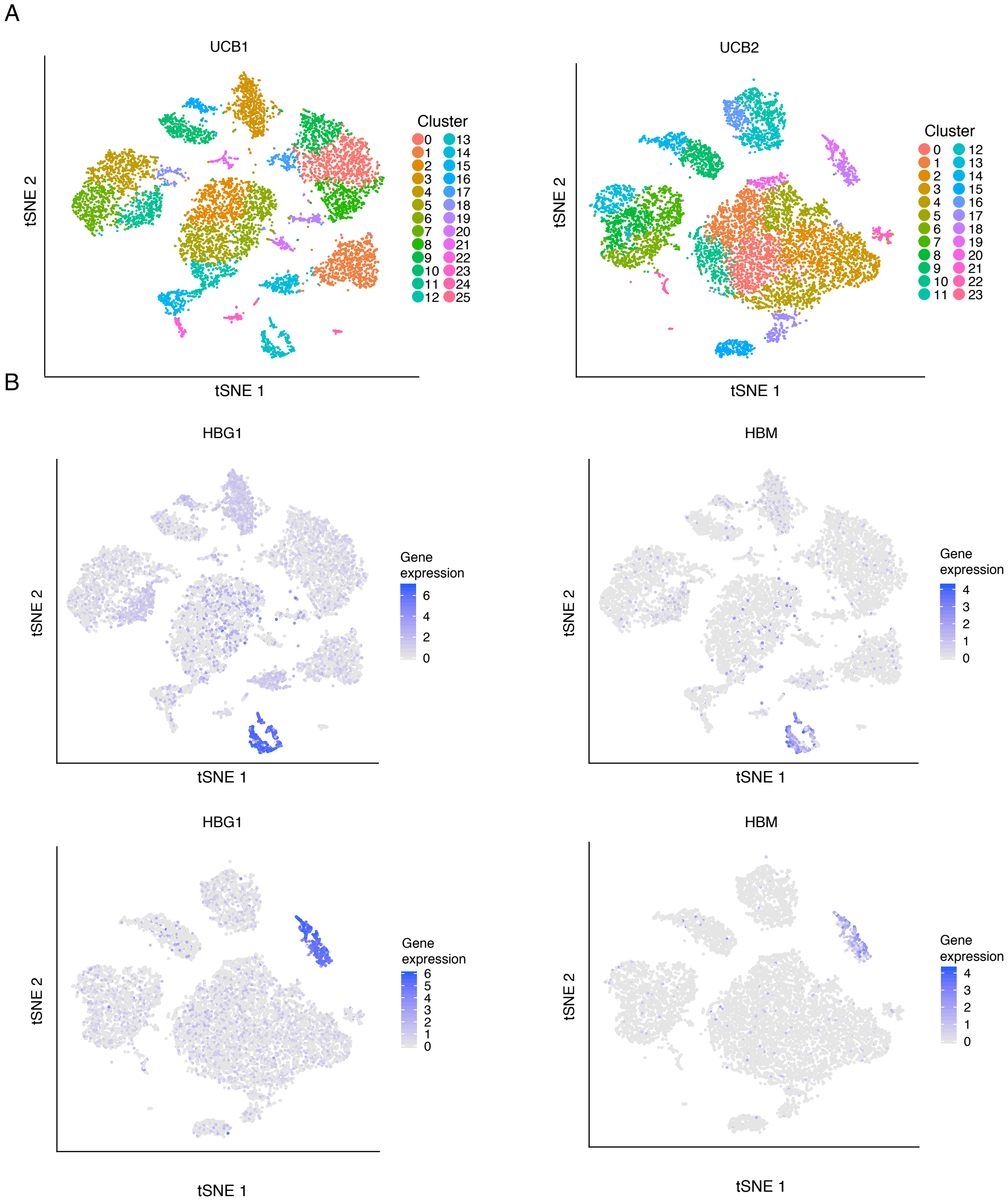

Supplementary Figure 2

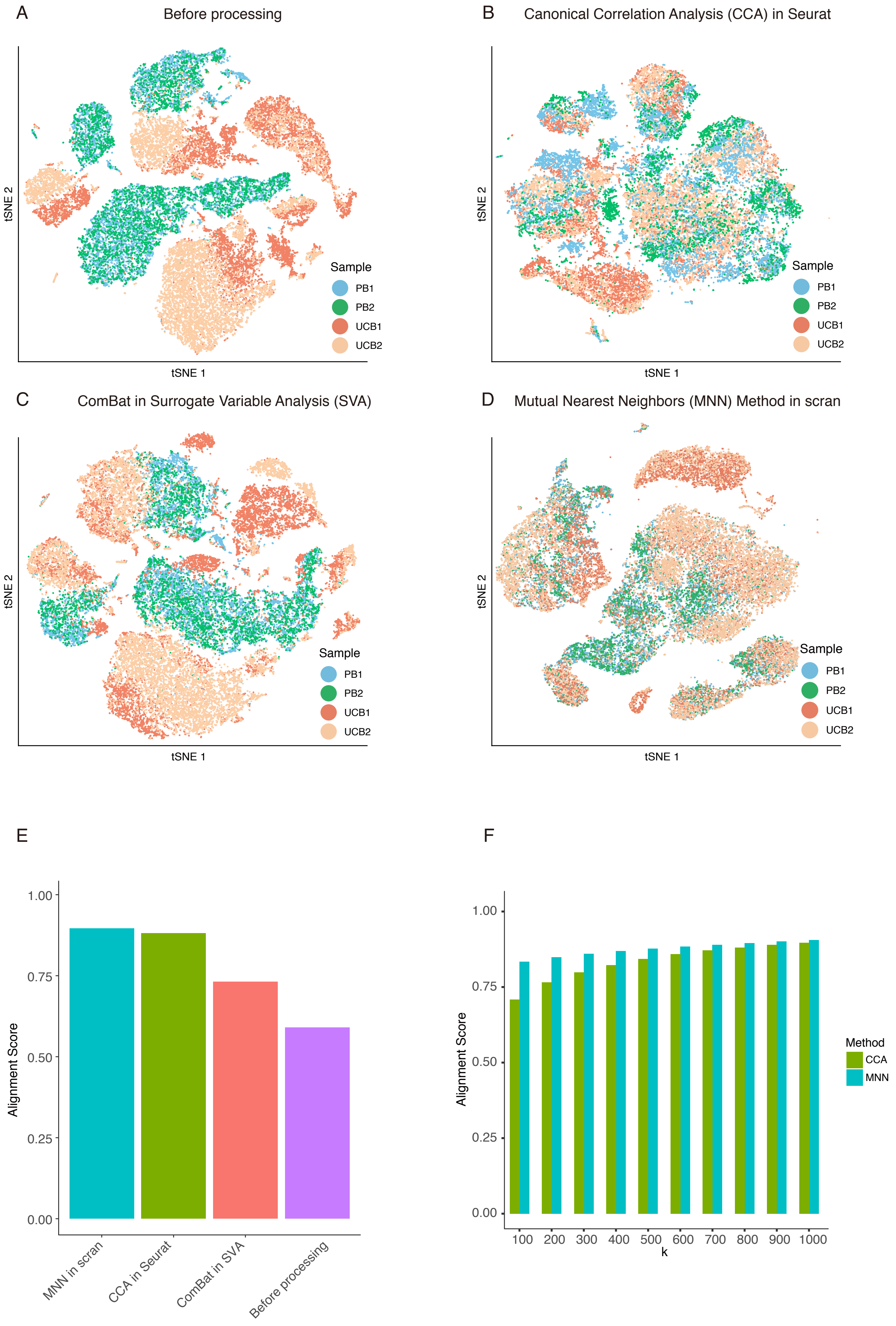

Supplementary Figure 3

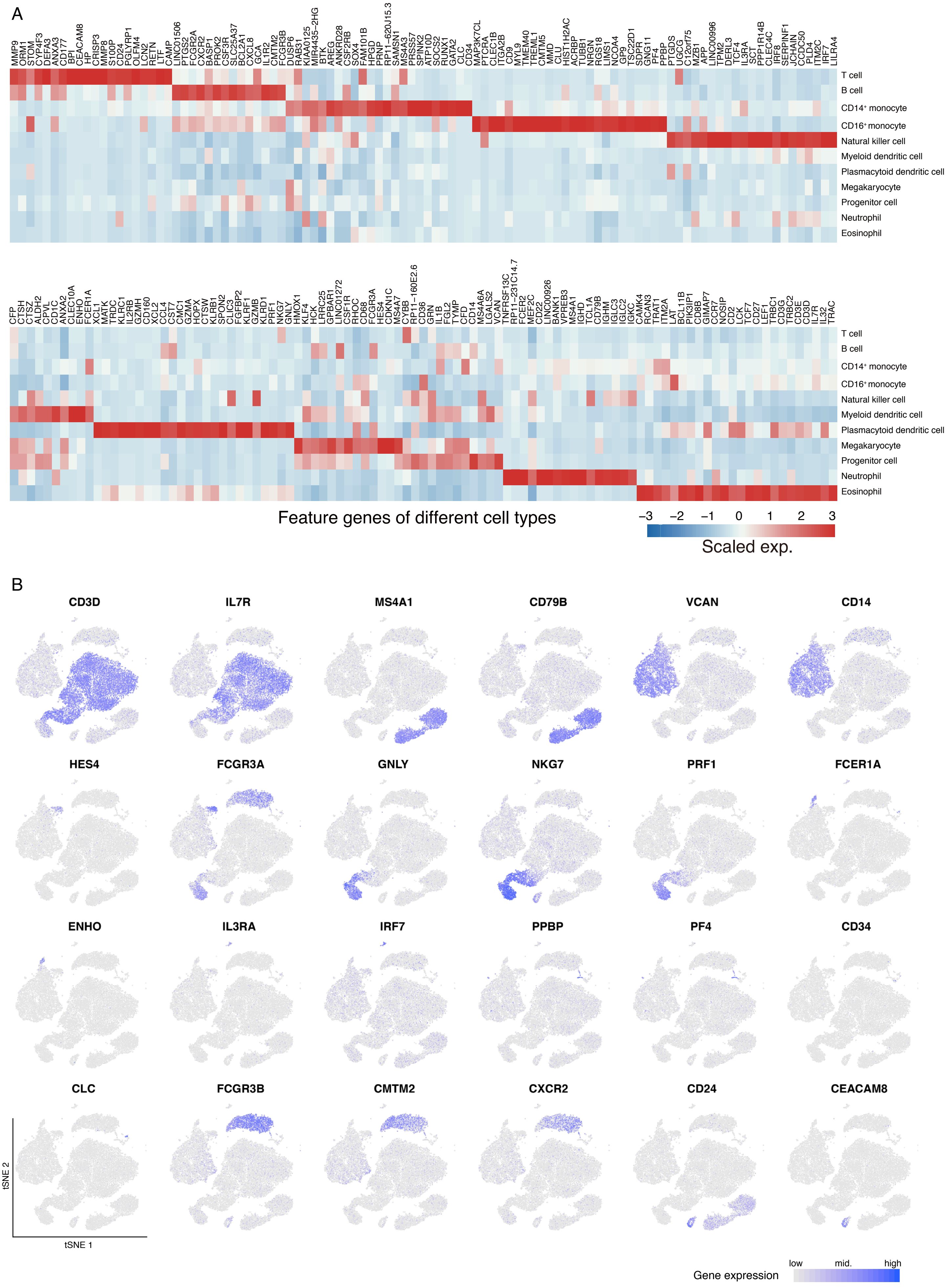

Supplementary Figure 4

A

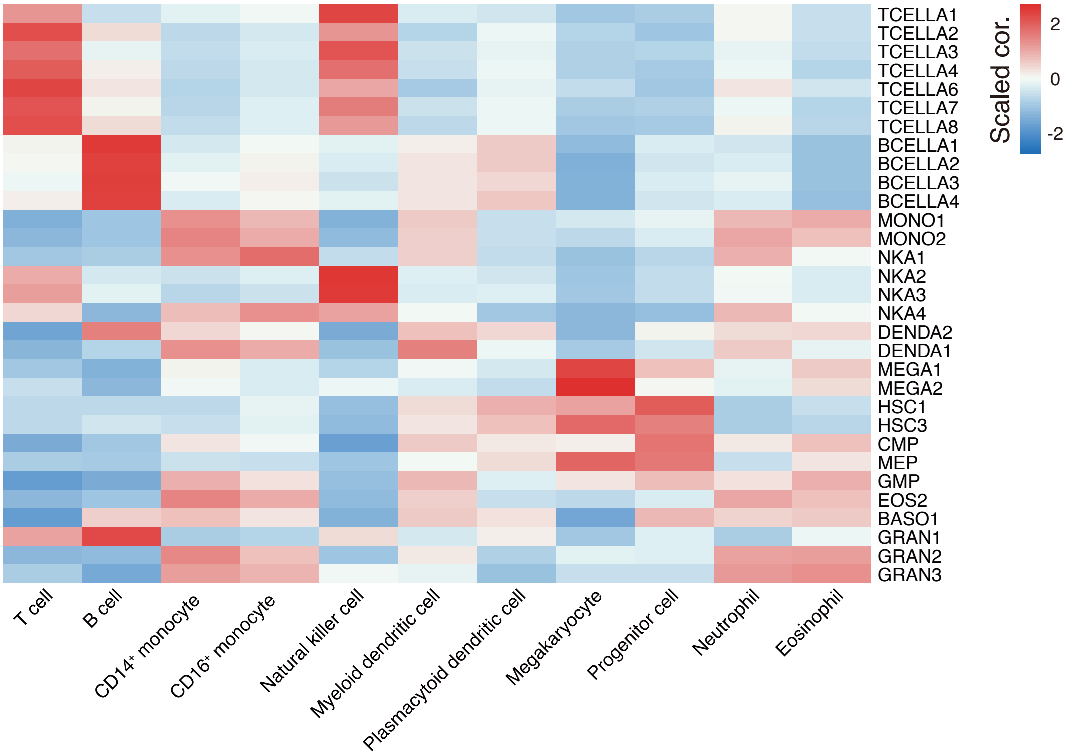

B

Cell type	PB1	PB2	UCB1	UCB2
T cell	4414	2247	1984	5287
B cell	1124	577	775	1025
CD14+ monocyte	1732	811	1663	1893
CD16+ monocyte	205	60	43	5
Natural killer cell	312	180	394	268
Myeloid dendritic cell	160	14	17	12
Plasmacytoid dendritic cell	65	13	30	14
Megakaryocyte	16	3	54	64
Progenitor cell	15	0	113	58
Neutrophil	0	0	2129	741
Eosinophil	0	0	347	54
NRBC	0	0	303	364

Supplementary Figure 5

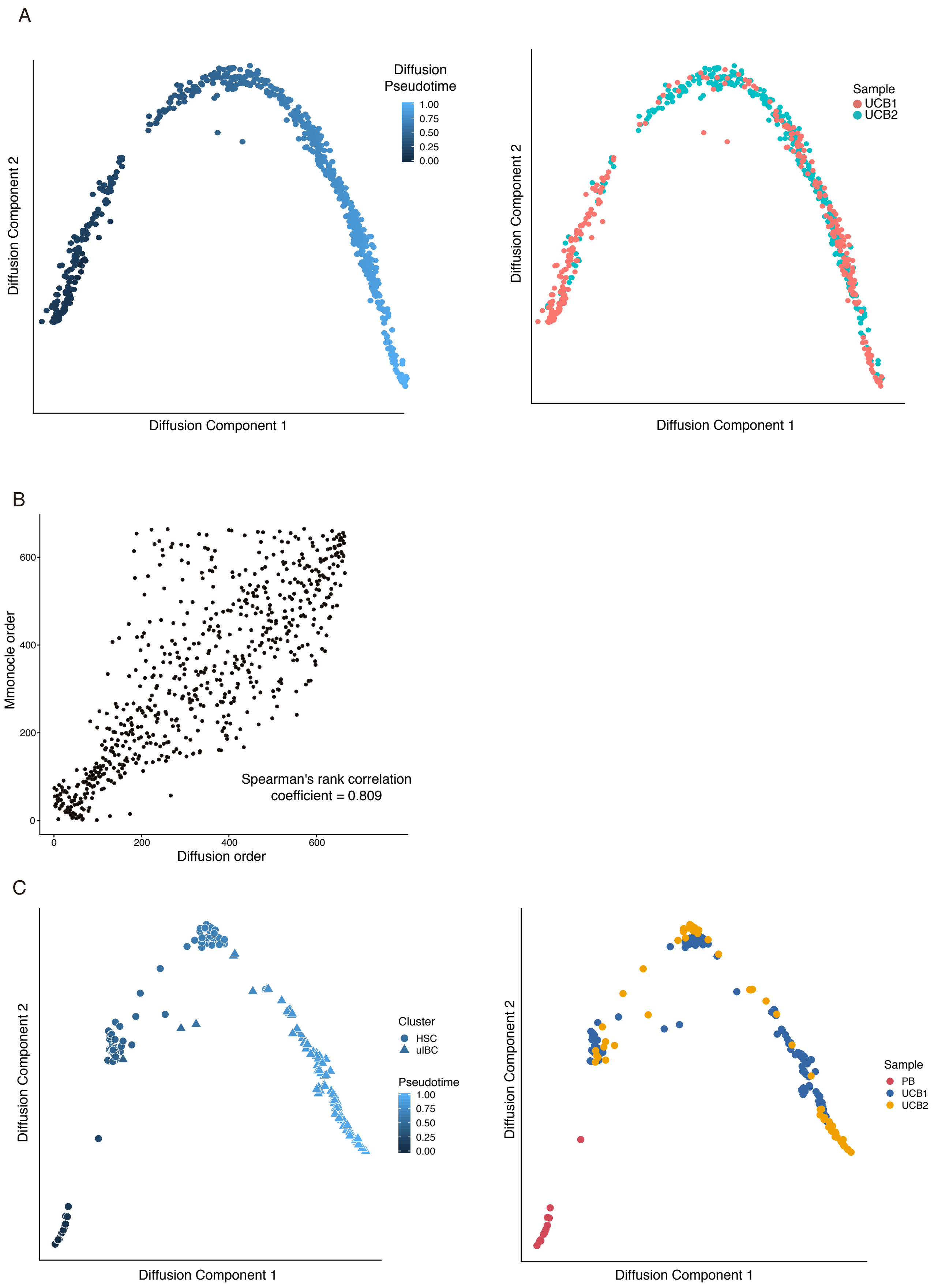

Supplementary Figure 6

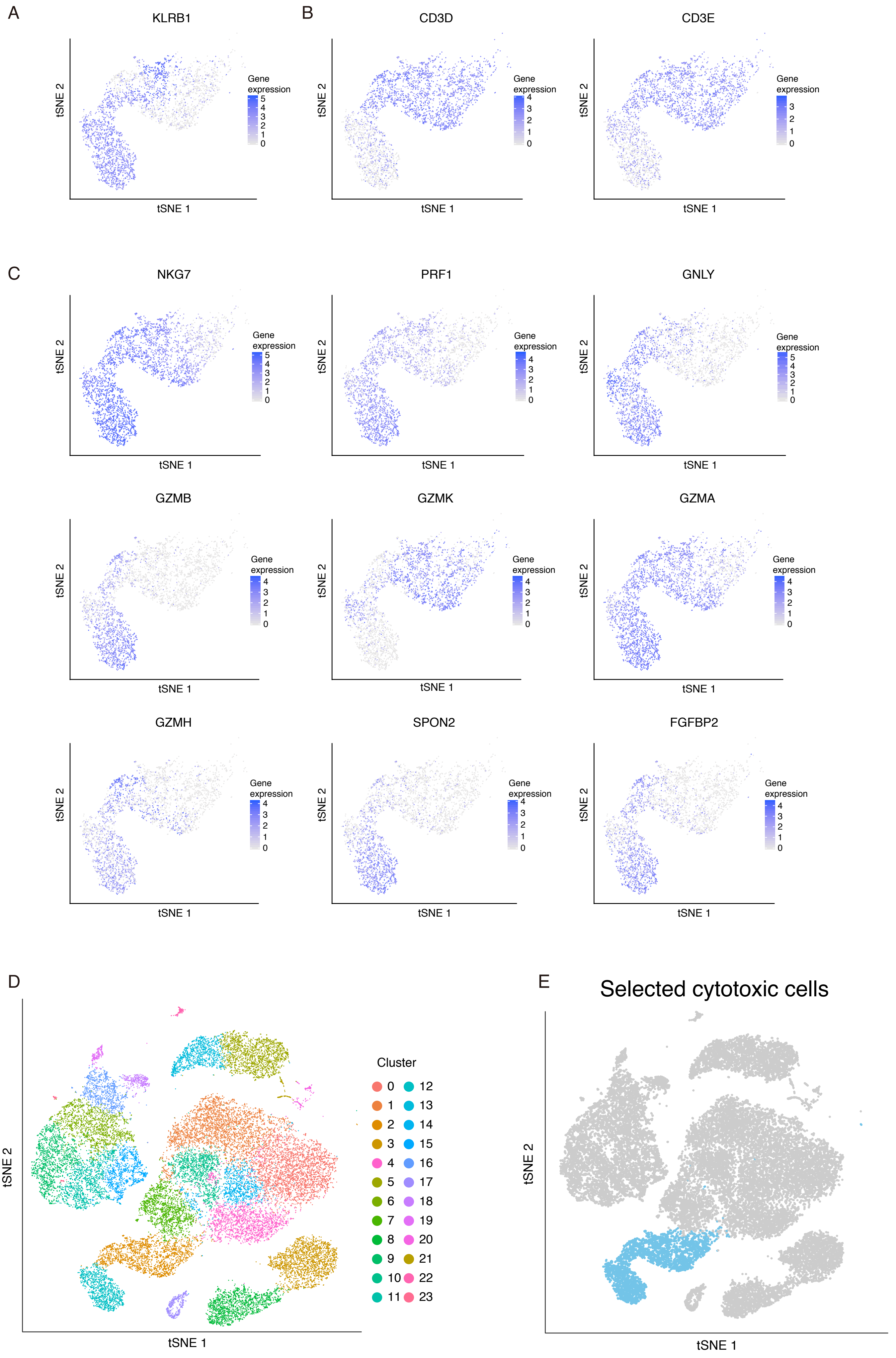

Supplementary Figure 7

A

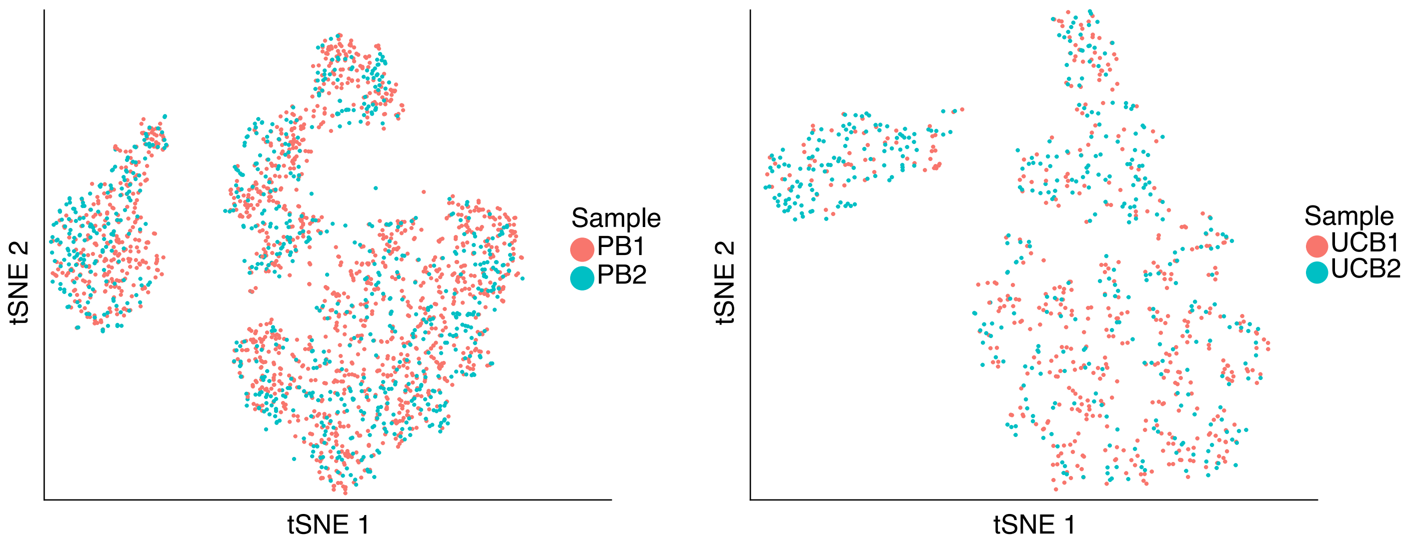

B

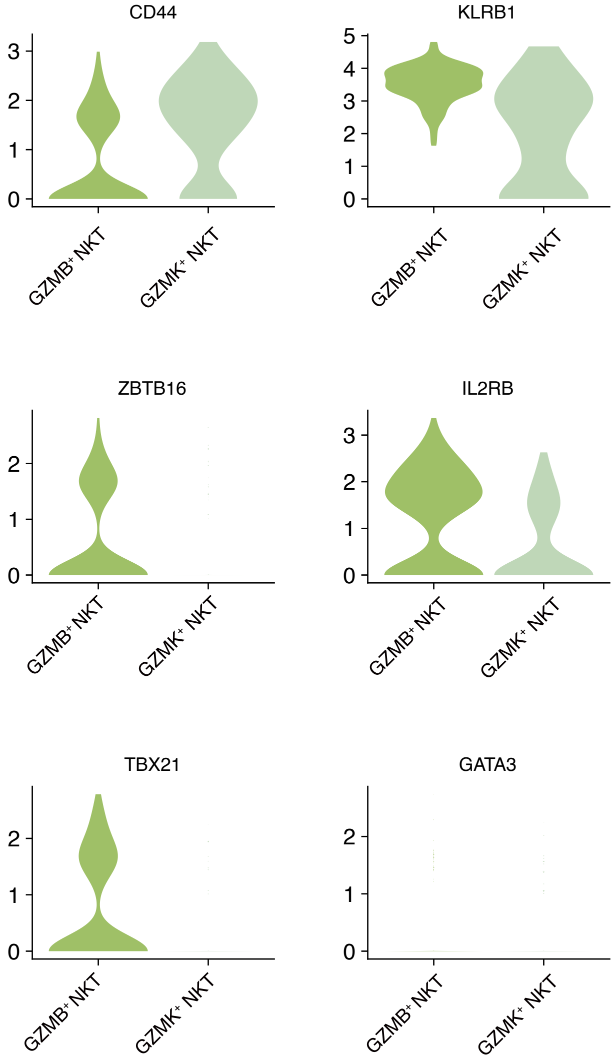

response to the reviewers

Click here to access/download
Supplementary Material
181119 Rebuttal.docx

Click here to download Personal Cover new cover.docx

Dear Dr. Scott Edmunds,

Enclosed please find the re-submission of our manuscript entitled “Single-cell Transcriptomic Landscape of Nucleated Cells in Umbilical Cord Blood” (GIGA-D-18-00231). Our manuscript provides the first single-cell landscape of nucleated cells in umbilical cord blood and detailed analysis of their characteristics, which will offer useful information for understanding and intervention of this important cell group. In our previous submission, the reviewers appreciated the merits of our study, but also raised extensive suggestions and concerns, especially in terms of data batch variations, which led to the open rejection of our manuscript.

After extensive re-analysis and detailed processing and comparisons of various pipelines, including significant efforts to minimize the batch effect of the data, we are now quite confident that we have comprehensively addressed all the concerns from the reviewers, and the quality of the manuscript has been significantly improved. Therefore, we sincerely hope you can re-evaluate our manuscript for publication in your journal.

We believe our work will be of broad interests to the readers of *Gigascience*. The manuscript has been approved by all the authors, and is in adhere to all the ethical guidelines. A detailed point-to-point response letter is attached to fully address all the concerns from the reviewers. We have also re-written the manuscript and **highlighted** the major additions and revisions from the former edition.

Thank you for all your help to improve the quality of the manuscript and we look forward to your favorable reply.

Xiao Liu, PhD
BGI-Shenzhen
