# Supplementary material for: Single-cell transcriptomic landscape of nucleated cells in umbilical cord blood
Source: Gigascience. 2019 May 2;8(5):giz047. doi: 10.1093/gigascience/giz047 (PMC6497034; doi:10.1093/gigascience/giz047)
Supplement: GIGA-D-18-00470_Revision_2.pdf [file giz047_giga-d-18-00470_revision_2.pdf]

GigaScience

Single-cell transcriptomic landscape of nucleated cells in umbilical cord blood

--Manuscript Draft--

**Manuscript Number:**	GIGA-D-18-00470R2
**Full Title:**	Single-cell transcriptomic landscape of nucleated cells in umbilical cord blood
**Article Type:**	Research
**Funding Information:**	Shenzhen Municipal Government of China (JCYJ20170817145404433) — Dr. Xiao Liu<br>Shenzhen Municipal Government of China (JCYJ20170817145428361) — Dr. Bin Li
**Abstract:**	Background<br><br>For both pediatric and adult patients, umbilical cord blood (UCB) transplant is a therapeutic option for a variety of hematologic diseases, such as blood cancers, myeloproliferative disorders, genetic diseases, and metabolic disorders. However, the level of cellular heterogeneity and diversity of nucleated cells in UCB has not yet been assessed in an unbiased and systemic fashion. In the current study, nucleated cells from UCB were subjected to single-cell RNA sequencing to simultaneously profile the gene expression signatures of thousands of cells, generating a rich resource for further functional studies. Here, we report the transcriptomes of 17,637 UCB cells, covering 12 major cell types, many of which can be further divided into distinct subpopulations.<br><br>Results<br><br>Pseudotemporal ordering of nucleated red blood cells identifies wave-like activation and suppression of transcription regulators, leading to a polarized cellular state, which may reflect nucleated red blood cell maturation. Progenitor cells in UCB also comprise two subpopulations with activation of divergent transcription programs, leading to specific cell fate commitment. Detailed profiling of cytotoxic cell populations unveiled granzymes B and K signatures in natural killer and natural killer T cell types in UCB.<br><br>Conclusions<br><br>Taken together, our data form a comprehensive single-cell transcriptomic landscape that reveals previously unrecognized cell types, pathways and mechanisms of gene expression regulation. These data may contribute to the efficacy and outcome of UCB transplant, broadening the scope of research and clinical innovations.
**Corresponding Author:**	Xiao Liu<br><br>CHINA
**Corresponding Author Secondary Information:**	
**Corresponding Author's Institution:**	
**Corresponding Author's Secondary Institution:**	
**First Author:**	Yi Zhao
**First Author Secondary Information:**	
**Order of Authors:**	Yi Zhao<br>Xiao Li<br>Weihua Zhao<br>Jingwan Wang<br>Jiawei Yu

Powered by Editorial Manager® and ProduXion Manager® from Aries Systems Corporation

	Ziyun Wan
	Kai Gao
	Gang Yi
	Xie Wang
	Bingbing Fan
	Qinkai Wu
	Bangwei Chen
	Feng Xie
	Jinghua Wu
	Wei Zhang
	Fang Chen
	Huanming Yang
	Jian Wang
	Xun Xu
	Bin Li
	Shiping Liu
	Yong Hou
	Xiao Liu
**Order of Authors Secondary Information:**	
**Response to Reviewers:**	We have edited and responded to all the comments from the copy editors, and provide a revised edition. Additionally, we have found some errors in the original supplementary figure 3, and provide a updated figure.
**Additional Information:**	
**Question**	**Response**
Are you submitting this manuscript to a special series or article collection?	No
**Experimental design and statistics**<br><br>Full details of the experimental design and statistical methods used should be given in the Methods section, as detailed in our Minimum Standards Reporting Checklist. Information essential to interpreting the data presented should be made available in the figure legends.<br><br>Have you included all the information requested in your manuscript?	Yes
**Resources**	Yes

Powered by Editorial Manager® and ProduXion Manager® from Aries Systems Corporation

A description of all resources used, including antibodies, cell lines, animals and software tools, with enough information to allow them to be uniquely identified, should be included in the Methods section. Authors are strongly encouraged to cite Research Resource Identifiers (RRIDs) for antibodies, model organisms and tools, where possible.<br><br>Have you included the information requested as detailed in our Minimum Standards Reporting Checklist?	
**Availability of data and materials**<br><br>All datasets and code on which the conclusions of the paper rely must be either included in your submission or deposited in publicly available repositories (where available and ethically appropriate), referencing such data using a unique identifier in the references and in the "Availability of Data and Materials" section of your manuscript.<br><br>Have you have met the above requirement as detailed in our Minimum Standards Reporting Checklist?	Yes

Click here to access/download;Manuscript;UCB ms 190330.docx
Click here to view linked References

Single-cell transcriptomic landscape of nucleated cells in umbilical cord blood

Yi Zhao[1,2,†], Xiao Li[2,†], Weihua Zhao[3,†], Jingwan Wang[2], Jiawei Yu[2], Ziyun Wan[2], Kai Gao[2], Gang Yi[4], Xie Wang[2], Bingbing Fan[3], Qinkai Wu[2], Bangwei Chen[2], Feng Xie[4], Jinghua Wu[2], Wei Zhang[2], Fang Chen[2], Huanming Yang[2,6], Jian Wang[2,6], Xun Xu[2], Bin Li[2,4,5], Shiping Liu[2], Yong Hou[2,*] and Xiao Liu[2,*]

[1]School of Biology and Biological Engineering, South China University of Technology, Guangzhou 510006, China; [2]BGI-Shenzhen, Shenzhen 518083, China; [3]Shenzhen Second People's Hospital, First Affiliated Hospital of Shenzhen University, Shenzhen 518035, Guangdong Province, China; [4]Shanghai Institute of Immunology, Shanghai JiaoTong University School of Medicine, Shanghai 200025, China; [5]Department of Immunology and Microbiology, Shanghai JiaoTong University School of Medicine, Shanghai 200025, China; [6]James D. Watson Institute of Genome Sciences, Hangzhou 310058, China

[†]Equal contributors

[*]Correspondence address: Xiao Liu, [Building No.11, Beishan Industrial Zone, Yantian District, Shenzhen 518083, Guangdong, China.]; Tel: [+86 13428700710]; email: [liuxiao@genomics.cn]

Yong Hou, [Building No.11, Beishan Industrial Zone, Yantian District, Shenzhen 518083, Guangdong, China.]; Tel: [+86 18588263403]; email: [houyong@genomics.cn]

ORCID IDs: Xiao Li: 0000-0001-8527-4438; Huanming Yang: 0000-0003-1703-3012; Yong Hou 0000-0002-0420-0726; Xiao Liu: 0000-0002-8073-0534

Abstract

Background: For both pediatric and adult patients, umbilical cord blood (UCB) transplant is a therapeutic option for a variety of hematologic diseases, such as blood cancers, myeloproliferative disorders, genetic diseases, and metabolic disorders. However, the level of cellular heterogeneity and diversity of nucleated cells in UCB has not yet been assessed in an unbiased and systemic fashion. In the current study, nucleated cells from UCB were subjected to single-cell RNA sequencing to simultaneously profile the gene expression signatures of thousands of cells, generating a rich resource for further functional studies. Here, we report the transcriptomes of 17,637 UCB cells, covering 12 major cell types, many of which can be further divided into distinct subpopulations.

Results: Pseudotemporal ordering of nucleated red blood cells identifies wave-like activation and suppression of transcription regulators, leading to a polarized cellular state, which may reflect nucleated red blood cell maturation. Progenitor cells in UCB also comprise two subpopulations with activation of divergent transcription programs,

leading to specific cell fate commitment. Detailed profiling of cytotoxic cell populations unveiled granzymes B and K signatures in natural killer and natural killer T cell types in UCB.

Conclusions: Taken together, our data form a comprehensive single-cell transcriptomic landscape that reveals previously unrecognized cell types, pathways and mechanisms of gene expression regulation. These data may contribute to the efficacy and outcome of UCB transplant, broadening the scope of research and clinical innovations.

Keywords: Umbilical cord blood, Single-cell RNA-seq, Transcriptomics, Nucleated red blood cell, Natural killer T cell

Introduction

Human umbilical cord blood (UCB) is an excellent source of hematopoietic progenitor cells. It has been widely used for bone marrow reconstitution since the 1980s [1, 2]. The progenitor cells contained in UCB can regenerate the entire lympho-hematopoietic compartment in the host. The most notable advantage of UCB transplant is the low risk of developing graft-versus-host disease (GVHD), even when donor and recipient are partially mismatched [3]. The immune cells in cord blood are virtually free from external stimulant and infection and thus are relatively more naïve. Such immunological immaturity is the key to alleviate the severity of GVHD by decreasing the alloreactive potential of lymphocytes [2, 4]. These advantages expand the clinical potential of UCB transplant in many cases, including some fatal diseases. The major limitation of UCB transplant, however, is the limited and inconsistent cell dose. It has

been shown that the success rate of engraftment was critically dependent on the number of nucleated cells in the donor UCB [4–6].

Although UCB is now widely used for important clinical applications, we know surprisingly little about its cellular and molecular characteristics. Specifically, the composition of progenitor, lymphocyte and other nucleated cells that affect the reconstitution potency after UCB engraftment is poorly understood. Recent advances in single-cell transcriptomics technology enable the exploration of cellular heterogeneity and deduction of functional relevance [7, 8]. Single-cell RNA-seq (scRNA-seq) studies of human peripheral blood (PB) cells have revealed new insights into immune cell composition and disease-related functional abnormalities [9–11]. Previous studies in mouse and human have focused on hematopoietic stem cell, erythroblast, and certain T cell subtypes, unveiling novel biological properties at the single-cell level [12–17]. However, scRNA-seq studies have not thoroughly characterized the major types of nucleated cells in UCB, especially erythrocyte and cytotoxic innate immune cells, despite their profound clinical significance. Thus, the current study aimed to investigate the nucleated cells present in UCB to depict a landscape view of the cellular composition and their transcriptomes. Such key information will undoubtedly facilitate clinical innovation to develop more efficient and cost-effective UCB transplantation.

Results

A single-cell transcription atlas of nucleated cells in umbilical cord blood

To acquire a transcriptomic map of umbilical cord blood (UCB) cells at single-cell resolution, we collected UCB from two healthy donors and isolated nucleated cells for single-cell RNA-sequencing using the 10× Chromium platform. After stringent quality control and filtering by multiple criteria (see Methods), transcriptomes of 7852 and 9785 single cells from the two UCB samples (UCB1 and UCB2) were acquired, detecting on average 1270 and 1460 genes per cell, respectively. To determine the unique cell subpopulations and the specific state of gene expression in UCB, we utilized the public single-cell transcriptomics dataset of peripheral blood (PB) cells for comparison. This dataset includes two independently generated libraries (PB1 and PB2), containing a total of 11,948 single-cell profiles of peripheral blood mononuclear cells (PBMC) measuring 1069 genes per cell on average. These are at a comparable level with those of the UCB data.

All four single-cell datasets were merged to enable a systematic comparison between UCB and PB cells. To identify cell populations based on their expression signatures, we analyzed the merged data using a typical pipeline in the Seurat software, including dimensionality reduction and subsequent unsupervised cell clustering [18]. However, when the data were visualized in a two-dimensional space by t-distributed stochastic neighborhood embedding (tSNE), we initially observed a strong segregation of UCB cells from PB cells regardless of cell type; a typical manifestation of batch effect. We also noticed that a group of UCB cells (3.92% of all UCB cells) that express massive amounts of hemoglobin genes, such as *HBG1* and *HBM* (Supplementary Fig. 1A and B), tend to significantly interfere with the merging of UCB cells with PB cells

and cell clustering, generating highly sample-segregated cell embeddings in the tSNE space (data not shown). Thus, prior to merging with the PB data, we excluded these cell clusters, which were later identified as nucleated red blood cells (NRBCs) and were further analyzed.

To isolate biological variance from the interfering technical variances in the remaining data, we employed three independent computational methods, canonical correlation analysis (CCA) [19], surrogate variable analysis (SVA) [20], and mutual nearest neighbors (MNN) [21], to systemically correct the potential technical variance (Supplementary Fig. 2A–D). We then quantitatively evaluated the corrected data using an alignment score-based method [19]. Results indicated that the MNN algorithm most successfully eliminated the batch effect in the current dataset (Supplementary Fig. 2E and F). Thus, we proceeded to use MNN-corrected expression matrices for the Seurat pipeline and all subsequent analysis.

A global view was generated to illustrate the cell composition landscape of UCB. Aside from the NRBCs, 11 distinct cell populations were clustered – based on their gene expression profiles – in both UCB samples. A merged PB dataset was clustered in parallel with UCB cells in the same tSNE space (Fig. 1A). All of the clusters identified were shared by the two UCB samples, demonstrating the robustness of our biological replicate (Supplementary Fig. 2D). Clusters of cells expressing known markers of major immune cell types were assigned with their respective identities (Fig. 1B, Supplementary Fig. 3A). The expression patterns of a few representative marker genes are shown as examples (Supplementary Fig. 3B). To further validate the cell type

annotations, we calculated transcriptome-wide correlations between cluster mean expression and previously characterized bulk RNA-seq profiles of sorted immune cell types, as reported in previous studies [22], which was in accordance with the annotation yielded by canonical marker genes (Supplementary Fig. 4A). Nine major immune cell types and hematopoietic lineages found in PB were identified in UCB, while neutrophils, eosinophils and the bioinformatically excluded NRBCs, were only present in the UCB data. The neutrophil and eosinophil discrepancy was expected because of different cell enrichment approaches used (see Methods) (Fig. 1C, Supplementary Fig. 4B). We focused the scope of current study on a few cell types with profound clinical applications. However, the cellulome landscape of UCB data constitutes a rich resource that can be used as a reference to complement transcriptomics analysis performed in bulk or single cell settings, as well as a guide to future functional studies.

Polarity of cord nucleated red blood cell

In mammalian hematopoiesis, NRBCs, or erythroblasts, undergo several developmental stages in the bone marrow, progressively decreasing cellular volume and RNA content, while accumulating specific functional proteins such as hemoglobin [23, 24]. It has been long known that erythroblasts exist in relatively large numbers in cord blood [25–27]. However, little is known about whether such developmental processes exist in the cord blood, or whether the erythroblast population is homogenous. In our dataset, we found that NRBCs constitute a significant proportion of the total nucleated cells in UCB (Supplementary Fig. 4B). Interestingly, NRBCs in the UCB

samples displayed pronounced polarity defined by the divergent expression of a gene repertoire. We employed Monocle2 software to identify differential genes among NRBCs, and deduced a pseudotemporal ordering of the cells that suggested a gradual change of cellular state [28] (see Methods). Evidently, the NRBCs from both UCB samples formed a linear trajectory along the pseudotime axis, with no significant branching, indicating that the cell polarity resulted from continuous changes of gene expression (Fig. 2A). To further validate the dual polarity of NRBCs in UCB, we employed an independent approach to construct a diffusion pseudotime map based on the transitions between cells using diffusion-like random walks [29] (Supplementary Fig. 5A). Cell ordering along the trajectories deduced by the two algorithms showed remarkable concordance (Supplementary Fig. 5B).

Next, we modeled gene expression along the Monocle2-inferred trajectory to identify genes characterized by a wave-like pattern. The most prominent were those genes encoding surface markers and proteins that are critical to the function of red blood cells, such as CD47, CD36, hemoglobin and glycophorins [30] (Fig. 2B). The CD47 molecule has long been considered as a cell surface marker of primitive erythrocytes [31]. Hemoglobin genes, in contrast, are highly expressed in the relatively mature form of NRBCs. Thus, the polarity observed here most likely reflected the maturity state of the NRBCs. An intermediate cell state that bridges the naïve state (CD47 high) and the mature state (hemoglobin high) was also observed. This intermediate stage was characterized by the elevated expression of a set of genes including those encoding glycophorins (*GYPA* and *GYPB*), suggesting that the cells in this stage exerted a

specific function, rather than being just transient intermediates. Strikingly, several key transcriptional regulators of erythrocyte homeostasis, including GATA1/2 and BCL11A [32–34], also clearly exhibited divergent patterns along the pseudotime axis (Fig. 2C). GATA1 is a well-characterized transcription factor responsible for the activation of multiple hemoglobin-encoding genes in erythroid ontogeny [35], while BCL11A is a transcription factor that silences hemoglobin-encoding genes [34]. Other examples were CITED2 and SOX6, transcription factors that have recently been characterized as signature molecules specifically expressed in mouse primitive and definitive erythroblasts, respectively. These showed similar specificity in the naïve and intermediate cellular states, as defined by the pseudotime axis [36]. To provide further evidence of this model, a gradual decrease in the numbers of RNA molecules (represented by unique molecular indices; UMI) (Fig. 2D) and expressed genes (Fig. 2E) across the pseudotime axis was observed, and permutation analyses demonstrated significant correlation between the gradual decrease and the pseudotemporal ordering of the cells (Supplementary Fig. 5C). This potentially reflects diminishing global gene expression activity caused by NRBC enucleation. These lines of evidence further corroborated the polarity identified in the NRBC population in UCB projected maturation progress, and strongly indicated that the differential activation of transcriptional programs was one of the underlining mechanisms.

Molecular signatures of UCB progenitor cell

A distinct progenitor population was found in UCB, which shared a similar transcriptome profile with the hematopoietic stem cells (HSCs) in the PB dataset (Fig. 1A, Supplementary Fig. 4A). However, when tSNE clustering was performed with the progenitor population at a finer resolution, a secondary subpopulation emerged, demonstrating the heterogeneity of the progenitor population in the UCB (Fig. 3A). One subpopulation of UCB progenitor cells overlapped with HSCs in PB and specifically expressed canonical HSC marker genes such as *CD34*, *SOX4* and *FLT3* (*CD135*) (Fig. 3B, triangles), suggesting their identity as cord blood HSCs. Interestingly, the other subpopulation comprised cells only from the UCB (Fig. 3B, dots) and did not express the HSC canonical markers (Fig. 3C, 3D) despite the similarity in overall spectrum of gene expression, which drove the clustered embeddings of these cells in the tSNE space. Surprisingly, this $CD34^{-}$ UCB-specific progenitor population highly expressed the myeloid lineage-specific gene *MS4A3* (Fig. 3D), a known signature of granulocytic-monocytic progenitors (GMPs) [37]. GMPs give rise to mast cell progenitors (MCP) and basophil progenitors (BPC), which are found in the bone marrow, spleen and gastrointestinal mucosa [38]. Furthermore, *FCER1A*, the gene encoding the Fc fragment of the IgE receptor, which is also a surface marker frequently used in cell sorting for mast cells [39], was highly expressed in the $CD34^{-}$ cell population; while *CCR3*, a sorting marker for basophils [40, 41], was co-expressed at a comparable level. Similarly, many genes with regulatory roles in mast cell and basophil differentiation, exemplified by *HDC* and *CSF2RB*, respectively [16, 38, 42], were also co-expressed at a high level (Fig. 3D). The concerted activation of gene repertoires

critical in GMP-MCP and GMP-BPC ontogeny axes strongly suggested that these cells were bi-potent progenitors or intermediate cells, similar to the basophil/mast cell progenitor (BMCP) first verified in spleens of adult mice [43]. A high level of GATA2 and a low level of CEBPA transcription factors was also consistent with the signatures of mouse BMCP [43–45] (Fig. 3D). Such expression signatures are also reminiscent of recently identified basophil/eosinophil/mast cell progenitors (Ba/Eo/Ma) in human cord blood and bone marrow [16, 46]. A critical difference between the UCB subpopulation and the mouse BMCP or human Ba/Eo/Ma is that *CD34* expression is turned off, suggesting limited stemness and differentiation commitment in these cells. We thus hypothesized that these cells represent intermediates before bifurcation during basophil and mast cell differentiation; we termed them umbilical intermediate bi-potent cells (uIBC). To further explore this hypothesis, we sought to use diffusion maps [29, 47] to characterize the trajectory of the speculated transition from HSC to uIBC. While a gradual shifting of identities from HSC to uIBC was observed on the first diffusion component, the uIBC side of the trajectory did not show a conclusive bifurcation towards mast cell and basophil lineages; this is likely because of the limited cell number (Supplementary Fig. 5D).

Next, we asked whether the switch of cell identities resulted from the alteration of transcriptional programming governing the differentiation process. Transcription factor enrichment analysis utilizing the Encode [48] and ChEA [49] databases was performed to detect over-represented combinations of conserved transcription factor binding sites in a given set of genes. The analysis revealed that TAF, YY1 and MYC

were mostly enriched for activating highly expressed genes found in the HSCs compared with uIBC (Fig. 3E). These transcription factors are well known for their roles in proliferation and cell cycle control [50–53]. Conversely, RUNX1, SPI1 and GATA2 were ranked as the top-enriched transcription factors for activating highly expressed genes in the uIBCs (Fig. 3E). These transcription factors are conventionally considered as master regulators of differentiation of the myeloid lineage [44, 54, 55]. Such functional correlation was further corroborated by the mutually exclusive expression pattern of the top-enriched factors. For example, high expression levels of *MYC*, *MAX* and *YY1*, enriched for activating HSC feature genes, were detected in the HSCs; and – vice versa – high expression levels of *SP1*, *GATA2* and *RUNX1*, were detected in the uIBC (Fig. 3F). These lines of evidence supported that the two subtypes of cells we found in the progenitor population in UCB were divergent on the hematopoietic axis and may have cord blood-specific functions.

Heterogeneity of cytotoxic innate immune cells

Effective immune response against infection, allergy and cancer generally requires coordinated activation of innate and adaptive immune systems. Recent studies have shown that natural killer (NK) T (NKT) cells emerge as a bridge between innate and adaptive immunity to mediate immune responses [56]. In the overall tSNE projection, NK cells were clustered as a contiguous ‘peninsula’ extending from the T cell population (Fig. 1A). Interestingly, *KLRB1*, a lineage marker of NK cells, was expressed in a gradient pattern across the two cell types, with no distinct boundary

(Supplementary Fig. 6A). Remarkably, the expression of *CD3D/E* was in a reversed gradient with that of *KLRB1* (Supplementary Fig. 6B), as well as those of the cytotoxic genes *NKG7*, *PRF1* and *GNLY* (Supplementary Fig. 6C). This pattern of expression indicated the existence of a group of cells with a bridging identity across the interface; most likely NKT cells. Unlike NK or T cells, NKT cells exhibit distinct tissue specificity under homeostatic conditions, suggesting compartmentalized functions [57-60]. To selectively investigate these cells, we utilized high-resolution clustering results generated by Seurat (see Methods), producing more detailed clusters of T and NK cells (Supplementary Fig. 6D), two of which corresponded to NK cells, the adjacent T cells and the bridging NKT cells that displayed gradient expression of *CD3D/E* and *KLRB1* (Supplementary Fig. 6E). The T cells in this cluster expressed CD8 but not CD4, thus were considered to be cytotoxic T cells (Supplementary Fig. 7A). We next carried out subclustering with these cells to further reveal heterogeneity. By relative expression levels of the lineage markers, and the fact that all of these cells express a spectrum of cytotoxic marker genes, such as *NKG7*, *PRF1* and *GNLY*, at high levels (Supplementary Fig. 6A-C), we assigned the cell identity as cytotoxic T lymphocytes (CTL) ($CD3^+CD4^-CD8^+KLRB1^-$), NK ($CD3^-KLRB1^+$) and NKT ($CD3^+KLRB1^+$) cells [58, 61, 62] (Fig. 4A).

Although CTL, NK and NKT cells were all present in the PB and UCB samples, the cell compositions were rather different. Apparent heterogeneity was observed in all three cell lineages, and – remarkably – was represented by the mutually exclusive expression of two granzyme genes, *GZMB* and *GZMK* (Fig. 4B). For example, the NK

and CTL cells in PB were each divided into two subgroups, specifically expressing *GZMB* and *GZMK* (Fig. 4C). Similarly, NK and NKT cells in UCB were also subgrouped into GZMK$^+$ and GZMB$^+$ populations (Fig. 4D). Thus, based on the expression of lineage markers and the two granzyme genes used for this classification scheme (Fig. 4E), a total of six distinct cell subtypes were defined. All subtypes found in UCB were consistent between donors (Supplementary Fig. 7B); however, both UCB donors lacked GZMB$^+$ CTL cells that were present in PB, possibly because of the lack of specific antigen stimulation. It was noteworthy that GZMB$^+$ NKT cells were abundantly detected in UCB, but were missing in PB, begging the question as to whether this particular subtype possessed specific functions. Collectively, the cell distribution of NKT and CTL indicated that UCB has stronger innate immunity and less adaptive immunity compared to PB. NKT cells were previously reported to have tissue-specific gene expression programs that lead to diverse functions, and were termed NKT1, NKT2 and NKT17, predominantly localized in liver, lung and peripheral lymph node, respectively [58, 63–66]. In our data, the expression profile of GZMB$^+$ NKT cells was mostly similar to that of the NKT1 type, highlighted by signature expression of *CD44*, *KLRB1*, *ZBTB16*, *IL2RB* and *TBX21* (Supplementary Fig. 7C). However, neither GZMB$^+$ or GZMK$^+$ cells expressed *GATA3*, a crucial transcription factor found in NKT2 and NKT17 [67, 68]. Together with the lack of *KLRB1* expression, the GZMK$^+$ NKT cell subtype is distinct from the known NKT2 or NKT17 subtypes [67, 68]. The enriched GZMB$^+$ NKT cells in UCB express a spectrum of chemokines and genes in cytotoxic pathways, which may mediate recruitment with other immune cell types to

coordinate an innate immune response (Fig. 4F). Gene ontology (GO) analysis further corroborated that the highly expressed genes of the GZMB$^+$ cells were enriched in innate cytotoxic immunity, such as neutrophil-mediated immunity, cellular response to infectious antigens and necrosis factors, while GZMK$^+$ cells were enriched in lymphocyte activation, lymphocyte cell–cell adhesion and chemotaxis pathways (Fig. 4G). Thus, we concluded that the cell composition of NKT and other cytotoxic cells varies between PB and UCB.

Unlike NKT, GZMK$^+$ and GZMB$^+$ NK subtypes were both present in PB and UCB (Fig. 4C and D). They may function differently because of their respective granzyme gene activation [69]. Recent studies have shown that the orchestrated expression of granzymes is part of the functional program that enables cytotoxic cells to exert specific functions [70, 71]. As exemplified by the NK subtypes, *GZMB* and *GZMK* expression represents such functional diversity, and highlights their respective cytotoxic gene expression programs. To reveal the elements of these two programs, we systemically compared the GZMB$^+$ subtypes of NK, NKT and CTL cells found in PB or UCB by testing the co-occurrence of signature genes specific to each subtype (see Methods). Among the four sets of signature genes – ranging from 116 to 144 in number – 31 signature genes were found to be shared by all four subtypes (Fig. 5A). Similarly, 22 signature genes were found to be common in the corresponding GZMK$^+$ subtypes (Fig. 5B). Permutation tests were performed to estimate the significance of the four-way intersection in both cases, and the resulting *P* values were both $< 3x10^{-16}$. These two sets of signature genes (31 and 22) that we found were defined as GZMB and

GZMK co-expressed genes, respectively, which were likely to contribute to the elimination of specific antigens. To corroborate these findings, we calculated the Pearson's correlation of cell-averaged expression of all 53 genes in GZMB$^+$ and GZMK$^+$ subtypes of NK and NKT cells in UCB, and CTL and NK cells in PB. As expected, unsupervised clustering revealed two major modules, corresponding to the GZMB and GZMK programs (Fig. 5C and D). Interestingly, within each program a smaller core module was discovered, highlighted by *EEF1A1*, *TPT1*, *COTL1* and *LTB* in the GZMK program; and *FGFBP2*, *PRF1*, *GZMA*, *FCGR3A* and *CCL4* in the GZMB program (Fig. 5C, red labeled genes). Similar analysis was performed in the PB cells. Here, the core modules were largely consistent with those of UCB, although the GZMK core module was less prominent (Fig. 5D, red labeled genes). The enriched genes identified in the two programs represent common features of the GZMB$^+$ and GZMK$^+$ subtypes of cytotoxic cells. They may serve as specific selection markers and targets for perturbation in further functional studies.

Discussion

Here, we present for the first time, a single cell-level transcriptomic landscape of nucleated cells in UCB. By analyzing the expression patterns of known marker genes, we identified UCB cells belonging to almost all of the major hematopoietic lineages in PB, covering lymphoid, myeloid and hematopoietic progenitor cells. We also observed that certain cell populations were highly enriched in UCB cells, such as NRBCs, uIBCs and GZMB$^+$ NKT cells. The features of these cells we discovered were consistent in

both UCB donors. However, it is important to keep in mind that the UCB donors' shared factors, such as genetic background, may contribute to the enrichment of these UCB-specific cell subtypes. A related technical challenge we encountered in the current study was the severe batch effect among sample types and donors. To minimize any technical variance that could lead to misinterpretation of the data, we rigorously tested three widely used algorithms for batch effect correction; namely, CCA, SVA and MNN. Based on a quantitative evaluation of cell segregation in the tSNE space, MNN and CCA appeared comparable and effective for our datasets, though MNN scored marginally higher.

In adults, red blood cells are mainly generated in the bone marrow from nucleated cells, which are identified as erythroid precursors. These cells undergo morphological changes throughout cell divisions, gradually decreasing in cell size and RNA species, and increasing in chromatin condensation and hemoglobin protein accumulation. Such changes have been associated with the early stages of maturation of red blood cells. In our dataset, we also observed such a dynamic cellular state in a linear polarity. While it is possible that the erythroid precursors at different stages in UCB may have migrated from the bone marrow, our findings also suggested that erythroid precursors might undergo a similar maturation process in the UCB.

Progenitor cell populations in UCB also appeared to be a mixture of at least two distinct subpopulations. It is conceivable that the HSC subpopulation ($CD34^{+}$) we identified is a mixture of HSC and various early multipotent progenitors committed to differentiation; these were termed 'primed progenitors' and were extensively discussed

in a recent profiling study of UCB HSC at the single cell level [16]. The lack of CD34 enrichment means that the UCB data in the current study have too few HSCs to recapitulate the heterogeneity reported in this study. The uIBC, a unique UCB subpopulation not seen in PB, was identified, with characteristics of both basophil and mast cell signatures. A similar bipotent population (BMCP) exists in mouse spleen, and is capable of divergent development [43]. Signature gene expression, including transcription factors and surface markers, were remarkably similar between BMCP and uIBC, except that uIBCs lack expression of the conventional progenitor marker CD34. Although uIBC and HSC in UCB were globally similar in their transcriptomic profiles, the lack of CD34 made it difficult to conclude whether or not these uIBCs were indeed progenitors, or transient intermediates captured during UCB hematopoiesis. The functional implication of their existence points to the developmental process downstream of the Ba/Eo/Ma primed branch detected in the previous study [16]; specifically, when the Ba/Eo/Ma primed cells lose stemness markers (e.g., CD34) and further express lineage genes. Functional validations are necessary to determine the potential abilities of self-renewal and lineage regeneration of these cells, and to substantiate the similarity with mouse BMCP or Ba/Eo/Ma primed cells at functional level.

Next, we interrogated the UCB single-cell data at a finer scale, and discovered unreported heterogeneity among CTL, NK and NKT cells in UCB, which appeared in different compositions and granzyme expression patterns to those in PB. It is noteworthy that the mutually exclusive pattern between the GZMA/B/perforin program

versus the GZMK program was a common feature in cytotoxic cell lineages in UCB and PB. This finding is consistent with previous studies performed in PB [69], demonstrating that human granzymes are differentially expressed in distinct subpopulations that may function outside of orchestrating cytotoxicity. Multiple recent studies utilizing single cell technologies have found that diversified expression of granzyme genes are indicative to T cell states under disease conditions, such as liver cancer, colorectal cancer, non-small cell lung cancer and HIV-1 infection. The consensus is that *GZMB*-expressing T cells tend to recapitulate the transcriptome of effective memory T cells, and *GMZK*-expressing T cells seem to be a transitional intermediate between effective and exhausted state [11, 71–73]. It is clear that such a pattern is not specific to disease conditions, as we now have shown that similar granzyme programs exist in cord blood CTL/NKT/NK cells as well. However, we did not find significant expression of exhausted marker genes in cord blood GMZK$^+$ CTLs, possibly because of the lack of constant antigen stimulus. The specification of GZMK$^+$ and GZMB$^+$ cells is likely to reflect different consecutive activation states, which might be interchangeable upon changes of the tissue microenvironment. Interestingly, a previously unknown NKT population, which may be unique to UCB, was identified as GZMB$^+$ NKT cells that do not express *GZMK* but highly express *GZMA*, *GZMH*, and *PRF1* genes instead, suggesting the activation of specific cytotoxicity mediated by granzyme and perforin pathways. NKT cells have an essential role in bridging innate and adaptive immunity against infectious diseases and tumorigenesis, thus they possess significant therapeutic value. UCB transplants have demonstrated remarkable

effectiveness in treating many types of blood cancers. Adoptive transfer of NKT cells has been tested in animal models [74, 75], and several clinical trials are underway to test the safety and efficiency of NKT cell transfer to harness solid tumors in humans [76–79]. Enhanced understanding of NKT cell heterogeneity in UCB would benefit our selection of appropriate sources, and activation of the cytotoxicity of NKT cells to target cancer and other diseases. Therefore, we speculated that a targeted enrichment, modulation or engineering of the existing NKT populations in UCB could lead to considerable improvement in the efficacy of enhancing protective immune responses.

Potential implications

Taken together, our data provide the first single cell transcriptomic references for UCB, which could be used as a standard dataset for comparative analysis. We expect that this dataset will prove useful in uncovering the novel molecular signatures that define the cellular heterogeneity of UCB, and will provide markers for targeted enrichment of certain cell types of interest to researchers in multiple fields. Our dataset is a rich resource to formulate hypotheses of signaling pathway activation, transcription control and other mechanistic studies in the field of functional immunology at the single cell level.

Methods

Sample collection

The project was reviewed and approved by the BGI-IRB and the ethics committee of Shenzhen Second People's Hospital under number 18120. Two umbilical cord blood (UBC) samples were collected from healthy donors immediately after caesarean section with informed consent. Samples were stored in ethylenediaminetetraacetic acid (EDTA) anticoagulant tubes and transported to the laboratory within 1 hour. $CD45^+$ and $CD45^-$ cells were isolated from 1 mL cord blood by positive and negative selection, respectively, using Whole Blood CD45 MicroBeads (Miltenyi,130-090-872) and a Whole Blood Column Kit (Miltenyi, 130-093-545). Next, a hemocytometer was used to count $CD45^+$ and $CD45^-$ cells, and these were mixed in a ratio of 4:1. The cells were gently pipetted into a single-cell suspension and diluted to concentration of 700 cell/μL. Public single-cell gene expression datasets of peripheral blood mononuclear cells (PB1 and PB2) were generated from a sample from a single donor. In the current study, PB1 and PB2 correspond to Cell Ranger 2.0.1-processed '8k PBMCs from a healthy donor' and '4k PBMCs from a healthy donor', respectively [80]

UCB library construction and sequencing

Single-cell suspensions of UCB samples were loaded to chips from the Single Cell 3′ Chip Kit (10× Genomics, USA) and subjected to the GemCode Single Cell Instrument (10× Genomics, USA) to generate single-cell gel beads in emulsion (GEMs), as per the manufacturer's instructions. Next, GEMs were subjected to library construction using Chromium™ Single Cell 3′ Reagent Kits v2 (10× Genomics, USA), the steps of which included incubation at room temperature, cDNA amplification, fragmentation, end

repair, A-tailing, adaptor ligation, and sample index polymerase chain reaction (PCR). Because this library was designed to be sequenced by the Illumina sequencing platform, we converted the libraries to be compatible with the BGISEQ-500 sequencer. To do so, we performed a 12-cycle PCR on the libraries using BGISEQ adaptor primers, with subsequent DNA circularization and rolling-cycle amplification (RCA) to generate DNA nanoballs (DNBs). Purified DNBs were sequenced using the BGISEQ-500 sequencer, generating reads containing 16 bp of 10X™ barcodes, 10 bp of unique molecular indices (UMI), and 100 bp of 3′ cDNA sequences. Each library was sequenced in three lanes, yielding ~1.9 billion reads in total [81–83].

Alignment and initial processing of sequencing data

The CellRanger toolkit (10X Genomics, USA, version 2.0.0) was used to align cDNA reads to the Genome Reference Consortium human build patch 38 (GRCh38) transcriptome. Filtered UMI expression matrices of both samples were generated using default parameters, and an additional “--force-cells=4000” parameter [84]. The expression matrices of all samples were first normalized using the “cellranger aggr” function in the CellRanger toolkit, with the parameter “--normalize=mapped”. As a result, raw expression data was generated for ~32,000 single cells of the UCB sample.

Quality filtration of cells

In accordance with published pipelines and quality control standards [18], abnormal cells in all datasets were uniformly filtered out based on their gene expression

distribution. A cell was considered as abnormal if any of the following criteria were met: (1) detected gene number below 400; (2) detected gene number higher than 2000, 2000, 3500, and 3000 for PB1, PB2, UCB1, and UCB2 datasets, respectively; and (3) more than 8%, 8%, 6% and 7% of detected genes are mitochondria genes in PB1, PB2, UCB1, and UCB2 datasets, respectively. A ‘detected gene’ is defined as any gene expressed in at least 30 individual cells at a level of UMI ≥1 in any given dataset. After filtering PB1, PB2, UCB1, and UCB2 datasets, 8380, 3977, 8981, and 9638 cells remained, respectively.

Cell clustering in individual UCB samples

Next, the filtered expression matrices of UCB1 and UCB2 were used for unsupervised cell clustering using Seurat (version 2.3.4), adopting the typical pipeline recommended by the authors (Seurat, RRID:SCR_016341) [18]. A total of 3113 (UCB1) and 2409 (UCB2) variable genes were used for the “RunPCA” function. Subsequently, the top 10 principal components (PCs) were subjected to the “FindClusters” and “RunTSNE” functions, with high-resolution setting at 2.0 (Supplementary Fig. 1A). In the dimensionally reduced t-distributed stochastic neighbor embedding (tSNE) space, clusters of nucleated red blood cells (NRBCs) were identified on the basis of the concerted expression of hemoglobin genes, such as *HBG1* and *HBM* (Supplementary Fig. 1B). Then, we bioinformatically isolated 672 NRBCs from UCB1 and UCB2 as a subdataset for further analyses. NRBC-excluded data were then subjected to merging and batch effect removal. NRBCs were excluded prior to data merging because we

noticed that the massively expressed hemoglobin genes significantly interfered with the merging of UCB cells with PB cells and cell clustering, yielding highly sample-segregated cell embeddings in the tSNE space, regardless of batch-removal methods or parameters used.

Correction of batch effects

Strong technical bias introduced by sample preparation, library construction and/or sequencing was observed in the merged data (Supplementary Fig. 2A). To evaluate the available strategy for batch correction, we independently tested surrogate variable analysis (SVA), canonical correlation analysis (CCA) and mutual nearest neighbor (MNN) analysis, and compared their outcomes. For the SVA method, we first log-transformed the expression values (as in log(exp + 1)), then used the ComBat function in the SVA package to minimize batch effects, with default parameters [20]. CCA was performed in Seurat to correct batch effects. Having tested different parameters, the best performance was observed when we used 15 canonical vectors and 1500 shared, high-variable genes.

For MNN, we first created a SingleCellExperiment object to store the counts and metadata for each sample, using SingleCellExperiment (version 1.3.10). These cells were pre-clustered using the quickCluster function. Size factors were computed for endogenous genes using the deconvolution method by computeSumFactors [85]. We then acquired normalized log-expression values and distinguished highly variable genes using the trendVar function, and decomposed the gene-specific variance into

biological and technical components using the decomposeVar function. To obtain a single set of features for batch correction, we computed the average biological component across all four batches. All genes with positive biological components were retained to ensure that biological variance was preserved. All batches were rescaled to account for differences in sequencing depth using the multiBatchNorm function. Lastly, the fastMNN function was applied to each of the four samples, using retained genes with the parameters k=50, d=50, approximate=TRUE, and auto.order=TRUE. Finally, corrected expression values for 3570 highly variable genes were generated using the tcrossprod function, and these expression values were used for downstream cell clustering and pseudotime analysis.

Evaluation of batch correction

Alignment scores of the methods described above were calculated based on tSNE plots, according to the strategy employed in a previous study [19]. First, neutrophils and eosinophils that were only present in UCB datasets were masked from the datasets. Then, we randomly sampled cells from the four datasets with the same number of cells, and constructed a nearest-neighbor graph based on their relative positions in tSNE space. For each sampled cell, we calculated the cell numbers from the dataset sample in the k nearest-neighbors and average with total cells to obtain $\overline{x}$. The alignment score was then calculated as following:

$$\text{Alignment score} = 1 - \frac{\overline{x} - \frac{k}{N}}{k - \frac{k}{N}}$$

Alignment scores were normalized by dataset size and scaled to range from 0 to 1. For Supplementary Figure 2E, the parameters used were k = 800, N = 4. As shown, the MNN alignment score was marginally higher than that of CCA. To rule out potential bias from the arbitrary selection of *k*, we tested different values of *k* from 100 to 1000, and observed that the high scores generated by MNN were independent of *k* selection (Supplementary Fig. 2F).

Cell type annotation

After batch correction by MNN, the merged expression matrix was further filtered following the typical Seurat pipeline. Specifically, ribosomal genes and cells with a mitochondrial gene UMI percentage high than 10% were removed, as well as cells with a total UMI count of more than 11,000. Then, the expression matrix was normalized using the NormalizeData function. The corrected expression matrix was used for dimensionality reduction following the typical Seurat pipeline. Next, 3556 variable genes in the batch-corrected expression matrix were used for RunPCA, ProjectPCA, FindClusters and RunTSNE functions with default parameters, except dims.use = 1:13 and resolution = 2.

Subsequently, the feature genes for each cluster were identified using normalized data with the Seurat FindAllMarkers function, with parameters min.pct=0.25, and thresh.use=0.25. Four minor clusters with ~5% (same as estimated by 10X Genomics, USA) total cells, which were suspected to be doublets because they shared feature genes from two adjacent large clusters, were removed from the datasets.

A total of 8043, 3905, 7852 and 9785 cells remained for annotation in PB1, PB2, UCB1, and UCB2 datasets, respectively (Supplementary Fig. 4B). The identity of each cell cluster was manually annotated by the specific expression of commonly known markers. Unsupervised annotation, by comparing averaged single cell expression levels with bulk RNA-seq data of sorted immune cells, was also performed to validate the results, as previously described [86]. Pearson's correlation was used to calculate the distance between the cell-averaged feature gene expression and the corresponding levels in bulk RNA-seq data (Supplementary Fig. 4A).

Pseudotime analysis of NRBCs

A total of 672 NRBCs, identified from the individually clustered UCB datasets, were directly merged for the following analysis. After removing five abnormal cells on account of their significantly deviated mitochondrial gene expression level (>2.5 %), 667 NRBCs were used to infer the developmental polarity of NRBCs. NRBCs were ordered according to the pseudotime deduced by 1859 ordering genes excluding ribosomal protein transcripts, which were differentially expressed (FDR < 0.05), using the "clusterCells" function in Monocle2 (version 2.6.4). The genes that changed as a function of pseudotime were further identified and clustered to allow visualization of modules of genes co-varying across pseudotime, according to the typical pipeline provided in the Monocle2 manual. The cluster-representing red blood cell effector, and known development-relevant genes for the heatmap plots, were further manually selected based on the literature. In parallel, a pseudotemporal trajectory was deduced

using the diffusion map API in Scanpy (python 3.6.6, scanpy 1.3.2), using default parameters (n_neighbors=20 and n_pcs=5 for the pre-processing.neighbors function, and n_comps=15 for the tools.diffmap function).

Diffusion map algorithm-generated pseudotemporal ordering of cells was compared with that of Monocle2 using Spearman's rank-order correlation (Supplementary Fig. 5B). Permutation analysis was performed by randomly shuffling the pseudotemporal ordering of cells 1000 times, and generating a distribution of Spearman's rank-order correlation coefficient. Student's t-test was then applied to estimate the statistical significance of deviation of the observed coefficient produced by Monocle pseudotime from the permutation distribution.

Clustering and pseudotime analysis of UCB progenitor cells

UCB progenitor cells were re-clustered using Seurat, as described for global clustering above. To visualize the potential transition of cell identities from hematopoietic stem cells (HSCs) to umbilical intermediate bi-potent cells (uIBCs), we used the diffusion map API in Scanpy to calculate the diffusion pseudotime trajectory; as in NRBCs analysis, default parameters were used, with the exception of n_pcs=6. Then, we used the FindAllMarkers function in Seurat, with the parameter min.pct=0.3, to find feature genes within the two clusters. To identify the divergent transcription factor programs in the two groups of cells, a web-based tool 'Enrichr' [87] was employed to analyze the enrichment of transcription factor binding on the signature genes set of each progenitor cell group [88].

Cytotoxic cell clustering and profiling

Cytotoxic cells of interest were selected by unsupervised clustering at resolution=2 using the FindClusters function in Seurat (Supplementary Fig. 6D). The two clusters (highlighted in Supplementary Fig. 6E) covering the gradient expression of multiple cytotoxic genes in Supplementary Fig. 6C were selected to create two new subdatasets, according to their respective sample type. Then, the two sets of UMI matrices (2271 cells in PB, and 879 cells in UCB) were subjected to a typical Seurat pipeline. The Seurat functions NormalizeData, RunPCA, ProjectPCA, FindClusters, and RunTSNEfunctions, with the parameters dims.use=1:3, and resolution=1.5 for UCB; and dims.use=1:8, and resolution=1.5 for PB, were sequentially applied. Subsequently, the cluster-specific genes used to annotate cell subtypes were identified using normalized data and the Seurat FindAllMarkers function, with the parameters min.pct=0.25, and thresh.use=0.25.

Signature gene selection in GZMK^{+} and GZMB^{+} subtypes

To identify common features of GZMK and GZMB (four-way-overlapped genes) programs in the cytotoxic cells (Fig. 5), GZMB/GZMK-expressing NK, NKTC and cytotoxic T cell (CTL) subtypes were used to create a new Seurat object using the SubsetData function. The function FindAllMarkers was used to identify corresponding feature genes of each cluster, with the parameters min.pct=0.25, and thresh.use=0.25.

The four-way Venn diagrams of feature genes shown in Fig. 5A and Fig. 5B were generated using the R package VennDiagram. To verify the statistical significance of the enrichment of the four-way-overlapped genes (GZMB/GZMK program genes), a one sample t-test was carried out by testing the mean number of overlapping genes from randomly sampled pools of genes. The sizes of these genes were kept the same as in the original feature genes in the four subtypes. The co-expression modules shown in Fig. 5C and Fig. 5D were identified by unsupervised clustering of Pearson's correlation of cell-averaged expression values.

Gene ontology analysis of UCB GZMB$^+$ NTK cells

To deduce the potential functions of the signature genes in UCB GZMB$^+$ NTK cells, gene ontology enrichment analysis was performed using clusterProfiler (version 3.8.1, RRID:SCR_016884) [89], with the top 100 feature genes of GZMK$^+$ NKT cells in UCB identified by Seurat. We then simplified the output from enrichGO by removing redundancy of enriched GO terms with the 'simplify' function.

Availability of source code and requirements

Not applicable.

Availability of supporting data and materials

The raw data reported in this study are deposited in the National Center for Biotechnology Information (NCBI) Sequence Read Archive (SRA) under bioproject number PRJNA524398, and in the CNGB Nucleotide Sequence Archive (CNSA)

(CNSA: https://db.cngb.org/cnsa/) with accession number: CNP0000090. Aligned sequences in CRAM format, and the gene expression matrix for every single cell, have also been uploaded to the *GigaScience* GigaDB repository [90].

List of abbreviations

UCB	umbilical cord blood
PB	peripheral blood
NK	natural killer cells
NKT	natural killer cells
PBMC	peripheral blood mononuclear cells
tSNE	t-distributed stochastic neighborhood embedding
CCA	canonical correlation analysis
SVA	surrogate variable analysis
MNN	mutual nearest neighbors
NRBC(s)	nucleated red blood cell(s)
UMI	unique molecular indices
HSC	hematopoietic stem cell
GMP	granulocytic-monocytic progenitor
BMCP	basophil/mast cell progenitor
uIBC	umbilical intermediate bi-potent cells
CTL	cytotoxic T lymphocytes
GO	Gene ontology

Declarations

Consent for publication

Not applicable.

Competing interests

Some of the authors are employed by BGI, including Yi Zhao, Xiao Li, Jingwan Wang, Jiawei Yu, Ziyun Wan, Kai Gao, Xie Wang, Qinkai Wu, Bangwei Chen, Jinghua Wu, Wei Zhang, Fang Chen, Huanming Yang, Jian Wang, Xun Xu, Bin Li, Shiping Liu, Yong Hou and Xiao Liu. The other authors declare no competing interests.

Funding

This work was supported by the Shenzhen Municipal Government of China (grant numbers JCYJ20170817145404433 and JCYJ20170817145428361)

Authors' contributions

X. Liu, Y.H., and S.L. jointly supervised the research. Y.Z., B.L., and G.Y. designed the experiments. X.W., K.G., Y.Z., and X.Z. performed the experiments. Y.Z. and Jingwan Wang pre-processed the sequencing data. Y.Z., X.Li., Jian Wang., Z.W., and Jingwan Wang analyzed the data. W. Zhao and B.F. collected the cord blood. X. Li, Y.Z., and X. Liu wrote the manuscript. X. Liu, Q.W., B.C., H.Y., F.C., Jian Wang, W. Zhang, X.X., and F.X. revised the manuscript. All authors have reviewed and approved the final manuscript.

Acknowledgments

We thank the two donors who generously provided the UCB samples. We also thank Lennart Hammarström for help editing the language, Liqin Xu, Zhikun Zhao for helpful discussions, and BGI colleagues who helped to produce the high-quality data.

References

1. Kurtzberg J. Update on umbilical cord blood transplantation. Current opinion in pediatrics. 2009;21 1:22-9.
2. Paloczi K. Immunophenotypic and functional characterization of human umbilical cord blood mononuclear cells. Leukemia. 1999;13 Suppl 1:S87-9.
3. Park SK and Won JH. Usefulness of umbilical cord blood cells in era of hematopoiesis research. International journal of stem cells. 2009;2 2:90-6.
4. Rocha V, Wagner JE, Jr., Sobocinski KA, Klein JP, Zhang MJ, Horowitz MM, et al. Graft-versus-host disease in children who have received a cord-blood or bone marrow transplant from an HLA-identical sibling. Eurocord and International Bone Marrow Transplant Registry Working Committee on Alternative Donor and Stem Cell Sources. The New England journal of medicine. 2000;342 25:1846-54. doi:10.1056/NEJM200006223422501.
5. Laughlin MJ, Barker J, Bambach B, Koc ON, Rizzieri DA, Wagner JE, et al. Hematopoietic engraftment and survival in adult recipients of umbilical-cord blood from unrelated donors. The New England journal of medicine. 2001;344

24:1815-22. doi:10.1056/NEJM200106143442402.

6. Migliaccio AR, Adamson JW, Stevens CE, Dobrila NL, Carrier CM and Rubinstein P. Cell dose and speed of engraftment in placental/umbilical cord blood transplantation: graft progenitor cell content is a better predictor than nucleated cell quantity. Blood. 2000;96 8:2717-22.

7. Navin NE. The first five years of single-cell cancer genomics and beyond. Genome research. 2015;25 10:1499-507. doi:10.1101/gr.191098.115.

8. Tanay A and Regev A. Scaling single-cell genomics from phenomenology to mechanism. Nature. 2017;541 7637:331-8. doi:10.1038/nature21350.

9. Eltahla AA, Rizzetto S, Pirozyan MR, Betz-Stablein BD, Venturi V, Kedzierska K, et al. Linking the T cell receptor to the single cell transcriptome in antigen-specific human T cells. Immunology and cell biology. 2016;94 6:604-11. doi:10.1038/icb.2016.16.

10. Proserpio V and Mahata B. Single-cell technologies to study the immune system. Immunology. 2016;147 2:133-40. doi:10.1111/imm.12553.

11. Zheng C, Zheng L, Yoo JK, Guo H, Zhang Y, Guo X, et al. Landscape of Infiltrating T Cells in Liver Cancer Revealed by Single-Cell Sequencing. Cell. 2017;169 7:1342-56 e16. doi:10.1016/j.cell.2017.05.035.

12. Gaublomme JT, Yosef N, Lee Y, Gertner RS, Yang LV, Wu C, et al. Single-Cell Genomics Unveils Critical Regulators of Th17 Cell Pathogenicity. Cell. 2015;163 6:1400-12. doi:10.1016/j.cell.2015.11.009.

13. Patil VS, Madrigal A, Schmiedel BJ, Clarke J, O'Rourke P, de Silva AD, et al.

Precursors of human CD4(+) cytotoxic T lymphocytes identified by single-cell transcriptome analysis. Science immunology. 2018;3 19 doi:10.1126/sciimmunol.aan8664.

14. Paul F, Arkin Y, Giladi A, Jaitin DA, Kenigsberg E, Keren-Shaul H, et al. Transcriptional Heterogeneity and Lineage Commitment in Myeloid Progenitors. Cell. 2015;163 7:1663-77. doi:10.1016/j.cell.2015.11.013.

15. Velten L, Haas SF, Raffel S, Blaszkiewicz S, Islam S, Hennig BP, et al. Human haematopoietic stem cell lineage commitment is a continuous process. Nature cell biology. 2017;19 4:271-81. doi:10.1038/ncb3493.

16. Zheng S, Papalexi E, Butler A, Stephenson W and Satija R. Molecular transitions in early progenitors during human cord blood hematopoiesis. Mol Syst Biol. 2018;14 3:e8041. doi:10.15252/msb.20178041.

17. Tusi BK, Wolock SL, Weinreb C, Hwang Y, Hidalgo D, Zilionis R, et al. Population snapshots predict early haematopoietic and erythroid hierarchies. Nature. 2018;555 7694:54-60. doi:10.1038/nature25741.

18. Satija R, Farrell JA, Gennert D, Schier AF and Regev A. Spatial reconstruction of single-cell gene expression data. Nat Biotechnol. 2015;33 5:495-502. doi:10.1038/nbt.3192.

19. Butler A, Hoffman P, Smibert P, Papalexi E and Satija R. Integrating single-cell transcriptomic data across different conditions, technologies, and species. Nat Biotechnol. 2018;36 5:411-20. doi:10.1038/nbt.4096.

20. Leek JT, Johnson WE, Parker HS, Jaffe AE and Storey JD. The sva package

for removing batch effects and other unwanted variation in high-throughput experiments. Bioinformatics. 2012;28 6:882-3. doi:10.1093/bioinformatics/bts034.

21. Haghverdi L, Lun ATL, Morgan MD and Marioni JC. Batch effects in single-cell RNA-sequencing data are corrected by matching mutual nearest neighbors. Nat Biotechnol. 2018;36 5:421-7. doi:10.1038/nbt.4091.

22. Novershtern N, Subramanian A, Lawton LN, Mak RH, Haining WN, McConkey ME, et al. Densely interconnected transcriptional circuits control cell states in human hematopoiesis. Cell. 2011;144 2:296-309. doi:10.1016/j.cell.2011.01.004.

23. Migliaccio AR. Erythroblast enucleation. Haematologica. 2010;95 12:1985-8. doi:10.3324/haematol.2010.033225.

24. Ji P, Murata-Hori M and Lodish HF. Formation of mammalian erythrocytes: chromatin condensation and enucleation. Trends in cell biology. 2011;21 7:409-15. doi:10.1016/j.tcb.2011.04.003.

25. Hebbar S, Misha M and Rai L. Significance of maternal and cord blood nucleated red blood cell count in pregnancies complicated by preeclampsia. Journal of pregnancy. 2014;2014:496416. doi:10.1155/2014/496416.

26. Hermansen MC. Nucleated red blood cells in the fetus and newborn. Archives of disease in childhood Fetal and neonatal edition. 2001;84 3:F211-5.

27. Merenstein GB, Blackmon LR and Kushner J. Nucleated red-cells in the newborn. Lancet. 1970;1 7659:1293-4.

28. Qiu X, Hill A, Packer J, Lin D, Ma YA and Trapnell C. Single-cell mRNA quantification and differential analysis with Census. Nat Methods. 2017;14 3:309-15. doi:10.1038/nmeth.4150.

29. Haghverdi L, Buttner M, Wolf FA, Buettner F and Theis FJ. Diffusion pseudotime robustly reconstructs lineage branching. Nat Methods. 2016;13 10:845-8. doi:10.1038/nmeth.3971.

30. van Schravendijk MR, Handunnetti SM, Barnwell JW and Howard RJ. Normal human erythrocytes express CD36, an adhesion molecule of monocytes, platelets, and endothelial cells. Blood. 1992;80 8:2105-14.

31. Oldenborg PA, Zheleznyak A, Fang YF, Lagenaur CF, Gresham HD and Lindberg FP. Role of CD47 as a marker of self on red blood cells. Science. 2000;288 5473:2051-4.

32. Dore LC and Crispino JD. Transcription factor networks in erythroid cell and megakaryocyte development. Blood. 2011;118 2:231-9. doi:10.1182/blood-2011-04-285981.

33. Bresnick EH, Hewitt KJ, Mehta C, Keles S, Paulson RF and Johnson KD. Mechanisms of erythrocyte development and regeneration: implications for regenerative medicine and beyond. Development. 2018;145 1 doi:10.1242/dev.151423.

34. Liu N, Hargreaves VV, Zhu Q, Kurland JV, Hong J, Kim W, et al. Direct Promoter Repression by BCL11A Controls the Fetal to Adult Hemoglobin Switch. Cell. 2018;173 2:430-42 e17. doi:10.1016/j.cell.2018.03.016.

35. Ohneda K and Yamamoto M. Roles of hematopoietic transcription factors GATA-1 and GATA-2 in the development of red blood cell lineage. Acta haematologica. 2002;108 4:237-45. doi:10.1159/000065660.

36. Kingsley PD, Greenfest-Allen E, Frame JM, Bushnell TP, Malik J, McGrath KE, et al. Ontogeny of erythroid gene expression. Blood. 2013;121 6:e5-e13. doi:10.1182/blood-2012-04-422394.

37. Ishibashi T, Yokota T, Satoh Y, Ichii M, Sudo T, Doi Y, et al. Identification of MS4A3 as a reliable marker for early myeloid differentiation in human hematopoiesis. Biochemical and biophysical research communications. 2018;495 3:2338-43. doi:10.1016/j.bbrc.2017.12.117.

38. Iwasaki H and Akashi K. Myeloid lineage commitment from the hematopoietic stem cell. Immunity. 2007;26 6:726-40. doi:10.1016/j.immuni.2007.06.004.

39. Stone KD, Prussin C and Metcalfe DD. IgE, mast cells, basophils, and eosinophils. The Journal of allergy and clinical immunology. 2010;125 2 Suppl 2:S73-80. doi:10.1016/j.jaci.2009.11.017.

40. Hausmann OV, Gentinetta T, Fux M, Ducrest S, Pichler WJ and Dahinden CA. Robust expression of CCR3 as a single basophil selection marker in flow cytometry. Allergy. 2011;66 1:85-91. doi:10.1111/j.1398-9995.2010.02431.x.

41. Chirumbolo S, Ortolani R and Vella A. CCR3 as a single selection marker compared to CD123/HLADR to isolate basophils in flow cytometry: some comments. Cytometry Part A : the journal of the International Society for

Analytical Cytology. 2011;79 2:102-6. doi:10.1002/cyto.a.21008.

42. Gurish MF, Tao H, Abonia JP, Arya A, Friend DS, Parker CM, et al. Intestinal mast cell progenitors require CD49dbeta7 (alpha4beta7 integrin) for tissue-specific homing. The Journal of experimental medicine. 2001;194 9:1243-52.

43. Arinobu Y, Iwasaki H, Gurish MF, Mizuno S, Shigematsu H, Ozawa H, et al. Developmental checkpoints of the basophil/mast cell lineages in adult murine hematopoiesis. Proc Natl Acad Sci U S A. 2005;102 50:18105-10. doi:10.1073/pnas.0509148102.

44. Iwasaki H, Mizuno S, Arinobu Y, Ozawa H, Mori Y, Shigematsu H, et al. The order of expression of transcription factors directs hierarchical specification of hematopoietic lineages. Genes & development. 2006;20 21:3010-21. doi:10.1101/gad.1493506.

45. Iwasaki H, Mizuno S, Mayfield R, Shigematsu H, Arinobu Y, Seed B, et al. Identification of eosinophil lineage-committed progenitors in the murine bone marrow. The Journal of experimental medicine. 2005;201 12:1891-7. doi:10.1084/jem.20050548.

46. Dahlin JS, Malinovschi A, Ohrvik H, Sandelin M, Janson C, Alving K, et al. Lin- CD34hi CD117int/hi FcepsilonRI+ cells in human blood constitute a rare population of mast cell progenitors. Blood. 2016;127 4:383-91. doi:10.1182/blood-2015-06-650648.

47. Coifman RR, Lafon S, Lee AB, Maggioni M, Nadler B, Warner F, et al. Geometric diffusions as a tool for harmonic analysis and structure definition

of data: diffusion maps. Proc Natl Acad Sci U S A. 2005;102 21:7426-31. doi:10.1073/pnas.0500334102.

48. Consortium EP. An integrated encyclopedia of DNA elements in the human genome. Nature. 2012;489 7414:57-74. doi:10.1038/nature11247.

49. Lachmann A, Xu H, Krishnan J, Berger SI, Mazloom AR and Ma'ayan A. ChEA: transcription factor regulation inferred from integrating genome-wide ChIP-X experiments. Bioinformatics. 2010;26 19:2438-44. doi:10.1093/bioinformatics/btq466.

50. Trop-Steinberg S and Azar Y. Is Myc an Important Biomarker? Myc Expression in Immune Disorders and Cancer. The American journal of the medical sciences. 2018;355 1:67-75. doi:10.1016/j.amjms.2017.06.007.

51. Amati B, Littlewood TD, Evan GI and Land H. The c-Myc protein induces cell cycle progression and apoptosis through dimerization with Max. The EMBO journal. 1993;12 13:5083-7.

52. Lin CY, Tuan J, Scalia P, Bui T and Comai L. The cell cycle regulatory factor TAF1 stimulates ribosomal DNA transcription by binding to the activator UBF. Current biology : CB. 2002;12 24:2142-6.

53. Lu Z, Hong CC, Kong G, Assumpcao A, Ong IM, Bresnick EH, et al. Polycomb Group Protein YY1 Is an Essential Regulator of Hematopoietic Stem Cell Quiescence. Cell Rep. 2018;22 6:1545-59. doi:10.1016/j.celrep.2018.01.026.

54. North TE, Stacy T, Matheny CJ, Speck NA and de Bruijn MF. Runx1 is

expressed in adult mouse hematopoietic stem cells and differentiating myeloid and lymphoid cells, but not in maturing erythroid cells. Stem cells. 2004;22 2:158-68. doi:10.1634/stemcells.22-2-158.

55. Dakic A, Metcalf D, Di Rago L, Mifsud S, Wu L and Nutt SL. PU.1 regulates the commitment of adult hematopoietic progenitors and restricts granulopoiesis. The Journal of experimental medicine. 2005;201 9:1487-502. doi:10.1084/jem.20050075.

56. Van Kaer L, Parekh VV and Wu L. Invariant natural killer T cells: bridging innate and adaptive immunity. Cell and tissue research. 2011;343 1:43-55. doi:10.1007/s00441-010-1023-3.

57. Bendelac A, Savage PB and Teyton L. The biology of NKT cells. Annual review of immunology. 2007;25:297-336. doi:10.1146/annurev.immunol.25.022106.141711.

58. Godfrey DI, MacDonald HR, Kronenberg M, Smyth MJ and Van Kaer L. NKT cells: what's in a name? Nature reviews Immunology. 2004;4 3:231-7. doi:10.1038/nri1309.

59. Kronenberg M. Toward an understanding of NKT cell biology: progress and paradoxes. Annual review of immunology. 2005;23:877-900. doi:10.1146/annurev.immunol.23.021704.115742.

60. Van Kaer L. NKT cells: T lymphocytes with innate effector functions. Current opinion in immunology. 2007;19 3:354-64. doi:10.1016/j.coi.2007.03.001.

61. Van Der Vliet HJ, Nishi N, Koezuka Y, Peyrat MA, Von Blomberg BM, Van

Den Eertwegh AJ, et al. Effects of alpha-galactosylceramide (KRN7000), interleukin-12 and interleukin-7 on phenotype and cytokine profile of human Valpha24+ Vbeta11+ T cells. Immunology. 1999;98 4:557-63.

62. Vivier E and Anfossi N. Inhibitory NK-cell receptors on T cells: witness of the past, actors of the future. Nature reviews Immunology. 2004;4 3:190-8. doi:10.1038/nri1306.

63. Gumperz JE, Miyake S, Yamamura T and Brenner MB. Functionally distinct subsets of CD1d-restricted natural killer T cells revealed by CD1d tetramer staining. The Journal of experimental medicine. 2002;195 5:625-36.

64. Lee PT, Benlagha K, Teyton L and Bendelac A. Distinct functional lineages of human V(alpha)24 natural killer T cells. The Journal of experimental medicine. 2002;195 5:637-41.

65. Coquet JM, Chakravarti S, Kyparissoudis K, McNab FW, Pitt LA, McKenzie BS, et al. Diverse cytokine production by NKT cell subsets and identification of an IL-17-producing CD4-NK1.1- NKT cell population. Proc Natl Acad Sci U S A. 2008;105 32:11287-92. doi:10.1073/pnas.0801631105.

66. Michel ML, Keller AC, Paget C, Fujio M, Trottein F, Savage PB, et al. Identification of an IL-17-producing NK1.1(neg) iNKT cell population involved in airway neutrophilia. The Journal of experimental medicine. 2007;204 5:995-1001. doi:10.1084/jem.20061551.

67. Brennan PJ, Brigl M and Brenner MB. Invariant natural killer T cells: an innate activation scheme linked to diverse effector functions. Nature reviews

Immunology. 2013;13 2:101-17. doi:10.1038/nri3369.

68. Constantinides MG and Bendelac A. Transcriptional regulation of the NKT cell lineage. Current opinion in immunology. 2013;25 2:161-7. doi:10.1016/j.coi.2013.01.003.

69. Bade B, Boettcher HE, Lohrmann J, Hink-Schauer C, Bratke K, Jenne DE, et al. Differential expression of the granzymes A, K and M and perforin in human peripheral blood lymphocytes. Int Immunol. 2005;17 11:1419-28. doi:10.1093/intimm/dxh320.

70. Bengsch B, Ohtani T, Herati RS, Bovenschen N, Chang KM and Wherry EJ. Deep immune profiling by mass cytometry links human T and NK cell differentiation and cytotoxic molecule expression patterns. J Immunol Methods. 2018;453:3-10. doi:10.1016/j.jim.2017.03.009.

71. Kiniry BE, Hunt PW, Hecht FM, Somsouk M, Deeks SG and Shacklett BL. Differential Expression of CD8(+) T Cell Cytotoxic Effector Molecules in Blood and Gastrointestinal Mucosa in HIV-1 Infection. J Immunol. 2018;200 5:1876-88. doi:10.4049/jimmunol.1701532.

72. Guo X, Zhang Y, Zheng L, Zheng C, Song J, Zhang Q, et al. Global characterization of T cells in non-small-cell lung cancer by single-cell sequencing. Nat Med. 2018;24 7:978-85. doi:10.1038/s41591-018-0045-3.

73. Zhang L, Yu X, Zheng L, Zhang Y, Li Y, Fang Q, et al. Lineage tracking reveals dynamic relationships of T cells in colorectal cancer. Nature. 2018;564 7735:268-72. doi:10.1038/s41586-018-0694-x.

74. Lam PY, Nissen MD and Mattarollo SR. Invariant Natural Killer T Cells in Immune Regulation of Blood Cancers: Harnessing Their Potential in Immunotherapies. Frontiers in immunology. 2017;8:1355. doi:10.3389/fimmu.2017.01355.

75. Bagnara D, Ibatici A, Corselli M, Sessarego N, Tenca C, De Santanna A, et al. Adoptive immunotherapy mediated by ex vivo expanded natural killer T cells against CD1d-expressing lymphoid neoplasms. Haematologica. 2009;94 7:967-74. doi:10.3324/haematol.2008.001339.

76. Exley MA, Friedlander P, Alatrakchi N, Vriend L, Yue S, Sasada T, et al. Adoptive Transfer of Invariant NKT Cells as Immunotherapy for Advanced Melanoma: A Phase I Clinical Trial. Clinical cancer research : an official journal of the American Association for Cancer Research. 2017;23 14:3510-9. doi:10.1158/1078-0432.CCR-16-0600.

77. Motohashi S, Ishikawa A, Ishikawa E, Otsuji M, Iizasa T, Hanaoka H, et al. A phase I study of in vitro expanded natural killer T cells in patients with advanced and recurrent non-small cell lung cancer. Clinical cancer research : an official journal of the American Association for Cancer Research. 2006;12 20 Pt 1:6079-86. doi:10.1158/1078-0432.CCR-06-0114.

78. Kunii N, Horiguchi S, Motohashi S, Yamamoto H, Ueno N, Yamamoto S, et al. Combination therapy of in vitro-expanded natural killer T cells and alpha-galactosylceramide-pulsed antigen-presenting cells in patients with recurrent head and neck carcinoma. Cancer science. 2009;100 6:1092-8.

doi:10.1111/j.1349-7006.2009.01135.x.

79. Yamasaki K, Horiguchi S, Kurosaki M, Kunii N, Nagato K, Hanaoka H, et al. Induction of NKT cell-specific immune responses in cancer tissues after NKT cell-targeted adoptive immunotherapy. Clinical immunology. 2011;138 3:255-65. doi:10.1016/j.clim.2010.11.014.

80. Single cell gene expression datasets for '4k PBMCs' and '8k PBMCs'. https://support.10xgenomics.com/single-cell-gene-expression/datasets. Accessed 30 March 2019.

81. Huang J, Liang X, Xuan Y, Geng C, Li Y, Lu H, et al. A reference human genome dataset of the BGISEQ-500 sequencer. Gigascience. 2017;6 5:1-9. doi:10.1093/gigascience/gix024.

82. Liang X: Protocols for BGISEQ-500 sequencing. https://www.protocols.io/view/bgiseq-500-sequencing-pq7dmzn. Accessed 29 March 2019.

83. Liang X: Protocols for BGISEQ-500 WGS library construction. https://www.protocols.io/view/bgiseq-500-wgs-library-construction-ps5dng6. Accessed 29 March 2019.

84. Zheng GX, Terry JM, Belgrader P, Ryvkin P, Bent ZW, Wilson R, et al. Massively parallel digital transcriptional profiling of single cells. Nat Commun. 2017;8:14049. doi:10.1038/ncomms14049.

85. Lun AT, Bach K and Marioni JC. Pooling across cells to normalize single-cell RNA sequencing data with many zero counts. Genome Biol. 2016;17:75.

doi:10.1186/s13059-016-0947-7.

86. Azizi E, Carr AJ, Plitas G, Cornish AE, Konopacki C, Prabhakaran S, et al. Single-Cell Map of Diverse Immune Phenotypes in the Breast Tumor Microenvironment. Cell. 2018;174 5:1293-308 e36. doi:10.1016/j.cell.2018.05.060.

87. Chen E, Kuleshov M, Jones M, Tan C, Kou Y, Clark N, et al.: Enrichr: interactive and collaborative HTML5 gene list enrichment analysis tool. http://amp.pharm.mssm.edu/Enrichr/. Accessed 30 March 2019.

88. Chen EY, Tan CM, Kou Y, Duan Q, Wang Z, Meirelles GV, et al. Enrichr: interactive and collaborative HTML5 gene list enrichment analysis tool. BMC Bioinformatics. 2013;14:128. doi:10.1186/1471-2105-14-128.

89. Yu G, Wang LG, Han Y and He QY. clusterProfiler: an R package for comparing biological themes among gene clusters. OMICS. 2012;16 5:284-7. doi:10.1089/omi.2011.0118.

90. Zhao Y; Li X; Zhao W; Wang J; Yu J; Wan Z; Gao K; Yi G; Wang X; Fan B; Wu Q; Chen B; Wu J; Zhang W; Chen F; Yang H; Wang J; Xu X; Li B; Liu S; Hou Y; Liu X (2019): Supporting data for "Single-cell Transcriptomic Landscape of Nucleated Cells in Umbilical Cord Blood" GigaScience Database. http://dx.doi.org/10.5524/100567

Figure legends

Figure 1: Cell types identified in the umbilical cord blood

(A) Global t-distributed stochastic neighbor embedding (tSNE) plots of merged umbilical cord blood (UCB) and peripheral blood (PB) cells. Cell clusters are colored to indicate cell types by expressed known markers. UCB cells are colorized in the left panel, and PB cells are colorized in the right panel. Cell types and their respective colors are labeled on the right. **(B)** Heatmap of scaled average gene expression of the major canonical markers (columns) detected in different cell types in merged cells of UCB and PB (rows). **(C)** Distribution of the abundance of each cell in each cell type in the PB and UCB datasets.

Figure 2: Polarity of nucleated red blood cells in the umbilical cord blood samples

(A) The order of nucleated red blood cells (NRBCs) along pseudotime in a two-dimensional space determined by Monocle2. Each dot represents a single NRBC. Color gradient represents the pseudotemporal order in the upper panel. Cells from the two UCB samples are labeled in the same topology in the bottom panel. **(B)** Heatmap of gene expression in NRBCs ordered by pseudotime (x-axis). Three clusters of pseudotime-dependent genes are grouped into primitive stage (top), intermediate stage (middle) and mature stage (bottom). **(C)** Heatmap of key transcription factor expression, similar to (B). **(D)** Numbers of detected UMI in each NRBC ordered by pseudotime. Each dot represents a NRBC, and the color represents the corresponding UCB sample of each cell. Y-axis represents number of detected unique molecular indices (UMI; thousands). Overall Spearman's correlation coefficient and corresponding *P* values are show at the top. **(E)** Numbers of detected genes in each NRBC, ordered by pseudotime.

Each dot represents an NRBC, and the color represents the corresponding UCB sample of each cell. Y-axis represents the number of detected genes (thousands). Overall Spearman's correlation coefficient and corresponding *P* values are show at the top.

Figure 3: Heterogeneous molecular signatures of progenitor cells in umbilical cord blood

(A) The re-clustered t-distributed stochastic neighbor embedding (tSNE) projection of progenitor cells from umbilical cord blood (UCB) and peripheral blood (PB) samples. The samples are labeled with different colors for each cell. **(B)** The two cell clusters, hematopoetic stem cells (HSC) and umbilical intermediate bi-potent cells (uIBC), are represented by triangles and dots, respectively. The color gradient represents the pseudotemporal order. **(C)** Heatmap of differentially expressed signature genes in the progenitors. Cells along the x-axis were ordered in the same way as in tSNE 1 axis in (A). The color bar on top denotes the HSC and uIBC clusters as well as the corresponding samples. **(D)** Violin plots of exemplary feature gene expressions of HSC (red) and uIBC (blue) cells. **(E)** Transcription factor enrichment analysis of HSC and uIBC cells using HSC signature genes (1012 genes, top left) and uIBC signature genes (106 genes, bottom left) revealed enriched transcription factors in HSC (top middle) and uIBC (bottom middle). Bar graphs of corresponding enrichment scores (-log false discovery rate, FDR) are shown on the right. **(F)** Violin plots of exemplary enriched transcription factor expression in HSC (red) and uIBC (blue) cells.

Figure 4: Heterogeneity of cytotoxic cells in peripheral blood and umbilical cord blood

(A) t-distributed stochastic neighbor embedding (tSNE) plots of re-clustered cytotoxic cells from peripheral blood (PB) (left) and umbilical cord blood (UCB; right) datasets. Each dot represents a single cytotoxic cell. Yellow color demonstrates high expression of CD3D; blue indicates KLRB1 highly expressed cell; red indicates cells highly expressing both CD3D and KLRB1; grey indicates cells expressing neither gene. **(B)** The tSNE plots with the same topology as in (A), with the gradient colors demonstrating the expression of GZMB and GZMK. **(C)** tSNE plots of cytotoxic cells from the PB datasets. Cell subtypes ($GZMK^+$ CTL, $GZMB^+$ CTL, $GZMK^+$ NKT, $GZMB^+$ NK, $GZMK^+$ NK) are labeled with different colors. **(D)** tSNE plots of cytotoxic cells from the UCB datasets. Cell subtypes ($GZMK^+$ CTL, $GZMK^+$ NKT, $GZMB^+$ NKT, $GZMB^+$ NK, $GZMK^+$ NK) are labeled with different colors. **(E)** Violin plots of signature gene expression of the subtypes in UCB (right) and PB (left). Color labeling of cell subtypes is consistent with that in (C). **(F)** Heatmap of exemplary differentially expressed signature genes in the $GZMB^+$ NKT and $GZMK^+$ NKT subtypes. The color bar on top denotes the $GZMB^+$ NKT and $GZMK^+$ NKT subtypes. **(G)** Gene ontology (GO) analysis of differentially expressed signature genes specific to $GZMB^+$ NKT (upper panel), and to $GZMK^+$ NKT (bottom panel) subtypes in UCB. The most enriched GO terms are ordered on the y-axis. X-axis represents the gene percentage in enriched GO terms. Sizes of the dots represent the number of genes included in each GO term. The color gradient of dots represents the adjusted *P*-values of each enriched GO term.

Figure 5: Enrichment of feature genes of granzyme B and K subtypes

(A) Four-way Venn diagrams reveal the enrichment of feature genes among granzyme B (GZMB)-positive cell types. **(B)** Four-way Venn diagrams reveal the enrichment of feature genes among granzyme K (GZMK)-positive cell types. **(C)** Pearson's correlation of expression of the four-way-overlapped genes in (A) and (B) from umbilical cord blood (UCB) datasets. **(D)** Pearson's correlation of expression of the four-way-overlapped genes in (A) and (B) in peripheral blood (PB) datasets.

Supplementary Figure 1: Pre-clustering of umbilical cord blood samples and exclusion of nucleated red blood cells

(A) Pre-clustering of cells in umbilical cord blood (UBC) dataset 1 (UCB1; left) and UCB2 (right). Each dot represents a single cell, and cells are color-labeled by cluster in t-distributed stochastic neighbor embedding (tSNE) space. **(B)** tSNE plots of the normalized expression of hemoglobin genes *HBG1* (left) and *HBM* (right) in UCB1 (top) and UCB2 (bottom). The color gradient represents the relative expression level.

Supplementary Figure 2: Sample distribution and evaluation of batch correction methods

(A) Sample distribution in t-distributed stochastic neighbor embedding (tSNE) space before batch removal processing. Cells are color-labeled by sample. **(B–D)** Sample distribution in tSNE space after canonical correlation analysis (CCA) (B), Combat (C)

and mutual nearest neighbor (MNN) (D) processes. Cells are color-labeled by sample in the same way as in (A). **(E)** Bar plot of alignment scores produced by different methods as shown in (A–D). **(F)** Comparison of alignment scores between CCA and MNN, with different parameters (from k=100 to k=1000).

Supplementary Figure 3: Signature gene expression of each cell type

(A) Heatmap of the scaled average gene expression of signature genes (column) detected in different cell types in umbilical cord blood (UCB) and peripheral blood (PB) (rows). **(B)** t-distributed stochastic neighbor embedding (tSNE) plots of the normalized expression of marker genes in the same global topology as in Fig. 1A. Each dot represents a single cell, and the color gradient represents the normalized gene expression.

Supplementary Figure 4: Cell type annotation composition

(A) Pearson's correlation between cell-averaged feature gene expression, with the corresponding levels in bulk RNA-seq data generated in sorted cells. **(B)** Table of cell numbers and percentages of different cell types in each sample.

Supplementary Figure 5: Pseudotime analysis in nucleated red blood cells and progenitor cells

(A) The order of nucleated red blood cells (NRBCs) along pseudotime in a two-dimensional space, as determined by diffusion map. Each dot represents a single NRBC.

Color gradient represents the pseudotemporal order in the left panel. Cells from the two umbilical cord blood (UCB) samples are labeled with the same topology as in the right panel. **(B)** Correlation between the pseudotemporal ordering of cells using Monocle and diffusion map. The correlation coefficient was calculated by Spearman's rank testing. **(C)** Permutation analysis of Spearman's correlation coefficient, as generated by Monocle pseudotemporal ordering (observed value) versus a distribution of coefficient generated by 1000-time randomly shuffled pseudotemporal orderings. Red vertical lines represent the observed values. *P* values were calculated by Student's t test. **(D)** The order of progenitor cells along pseudotime in a two-dimensional space, as determined by diffusion map. Each dot represents a single cell, and the color gradient represents the order of pseudotime (left). The sample distribution along the pseudotime, and the color, represents the corresponding sample (right).

Supplementary Figure 6: Cytotoxic signature gene expression in natural killer and natural killer T cell populations

(A–C) Zoomed-in t-distributed stochastic neighbor embedding (tSNE) plots of the normalized expression of cytotoxicity and related genes of the cytotoxic cell. Each dot represents a single cell, and the color gradient represents normalized gene expression. **(D)** Unsupervised high-resolution clustering of merged peripheral blood (PB) and umbilical cord blood (UCB) cells, in the same tSNE topology as in Fig.1A. Clusters are labeled by different colors. **(E)** As for (D), cells with cytotoxic features that are further analyzed are highlighted in blue color.

Supplementary Figure 7: Differential gene expression in natural killer and natural killer T cell subpopulations

(A) Global expression patterns of T cell subtype markers CD4, CD8A and CD8B. Lower right panel illustrates the T cell subtype distribution determined by the markers. **(B)** Cells are color-labeled by samples in the same t-distributed stochastic neighbor embedding (tSNE) space as in Fig. 4C and Fig. 4D. Each dot represents a single cell in peripheral blood (PB; left) and umbilical cord blood (UCB; right). **(C)** Violin plots show the scaled expression of indicated differential genes between granzyme B-positive (GZMB$^+$) natural killer T cell (NKT) and granzyme K-positive (GZMK$^+$) NKT subsets in UCB.

errors corrected in the original figure

Click here to access/download
Supplementary Material
update Supplementary Figure 3.pdf
