# Supplementary material for: Single-cell transcriptomic landscape of nucleated cells in umbilical cord blood
Source: Gigascience. 2019 May 2;8(5):giz047. doi: 10.1093/gigascience/giz047 (PMC6497034; doi:10.1093/gigascience/giz047)

Supplementary Figure 1

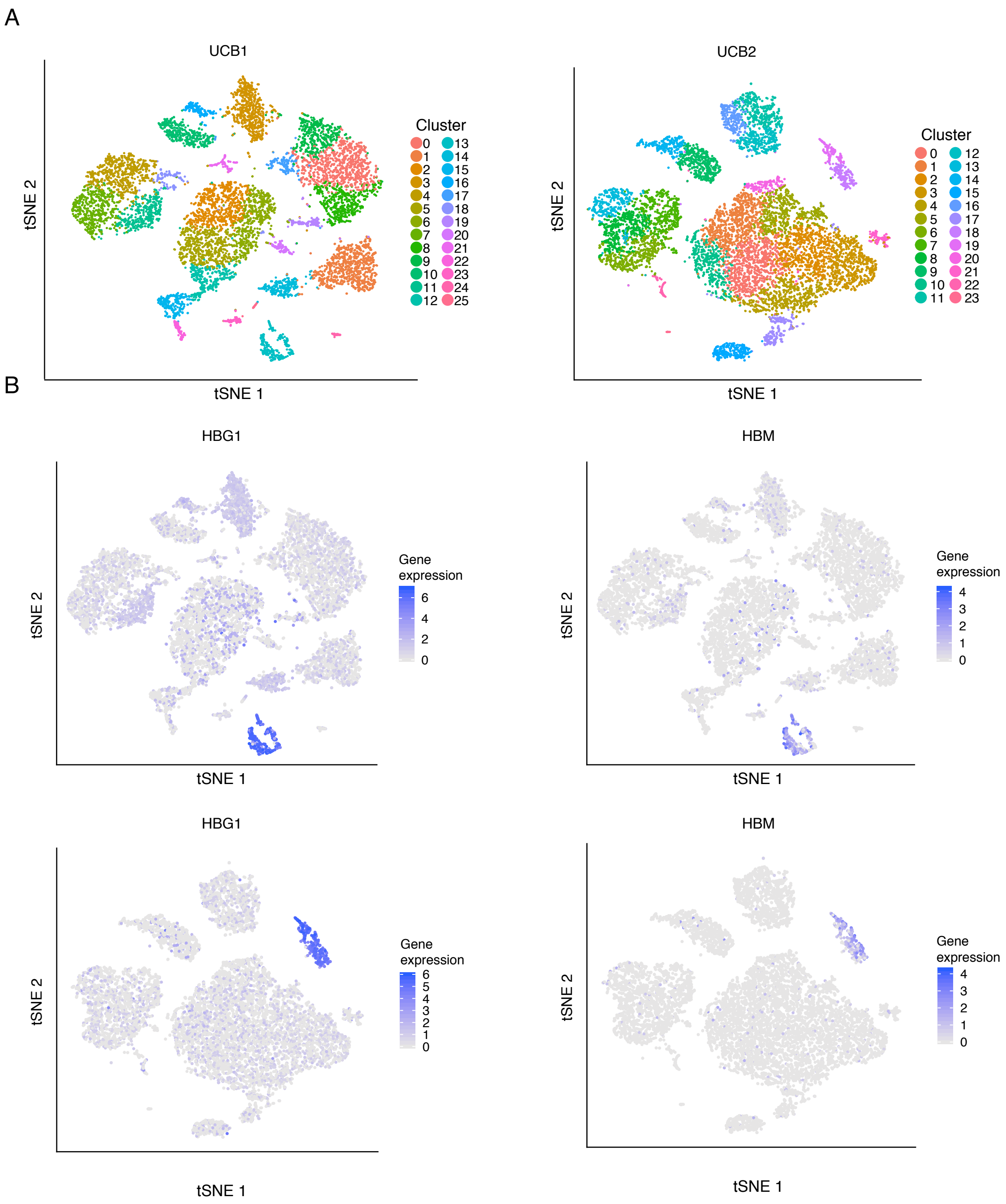

Supplementary Figure 2

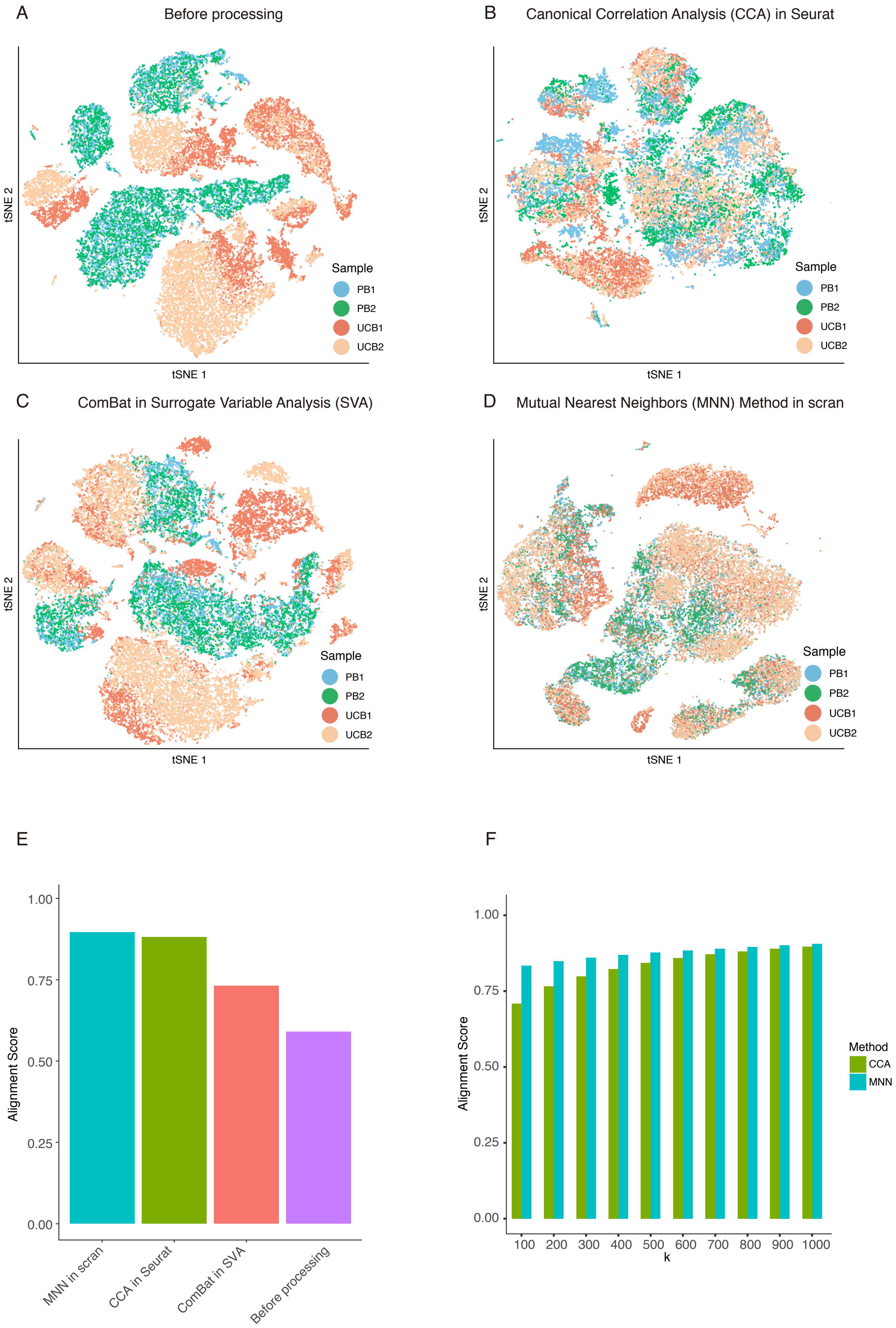

Supplementary Figure 3

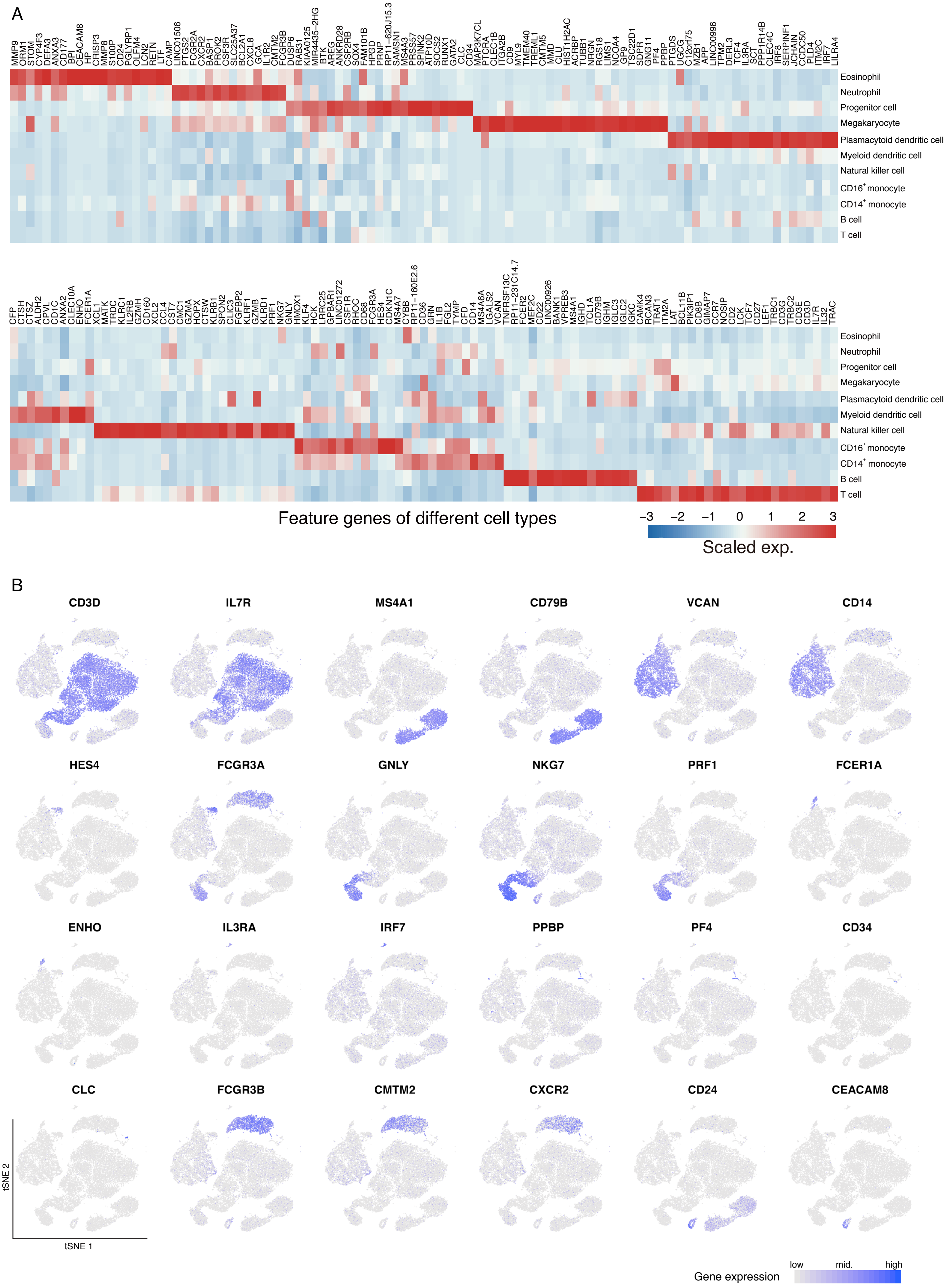

Supplementary Figure 4

A

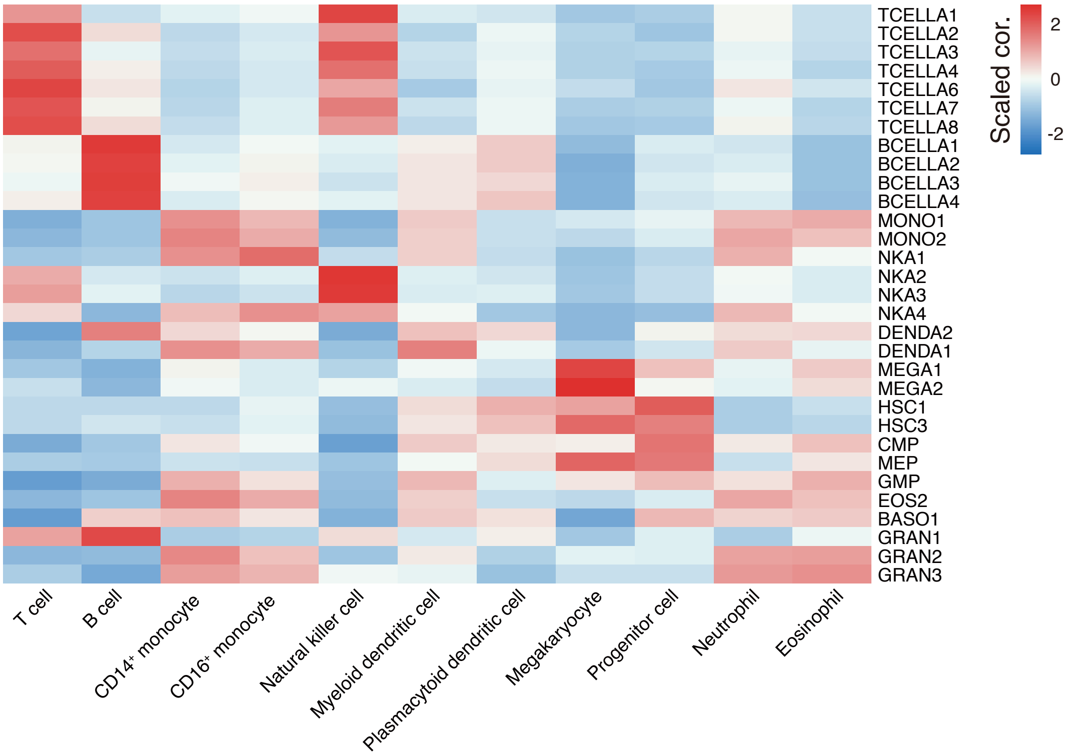

B

Cell type	PB1	PB2	UCB1	UCB2	PB1	PB2	UCB1	UCB2
	cell number				cell percentage			
T cell	4414	2247	1984	5287	54.88	57.54	25.27	54.03
B cell	1124	577	775	1025	13.97	14.78	9.87	10.48
CD14$^+$ monocyte	1732	811	1663	1893	21.53	20.77	21.18	19.35
FCGR3A$^+$ monocyte	205	60	43	5	2.55	1.54	0.55	0.05
Natural killer cell	312	180	394	268	3.88	4.61	5.02	2.74
Myeloid dendritic cell	160	14	17	12	1.99	0.36	0.22	0.12
Plasmacytoid dendritic cell	65	13	30	14	0.81	0.33	0.38	0.14
Megakaryocyte	16	3	54	64	0.20	0.08	0.69	0.65
Progenitor cell	15	0	113	58	0.19	0	1.44	0.59
Neutrophil	0	0	2129	741	0	0	27.11	7.57
Eosinophil	0	0	347	54	0	0	4.42	0.55
NRBC	0	0	303	364	0	0	3.86	3.72

Supplementary Figure 5

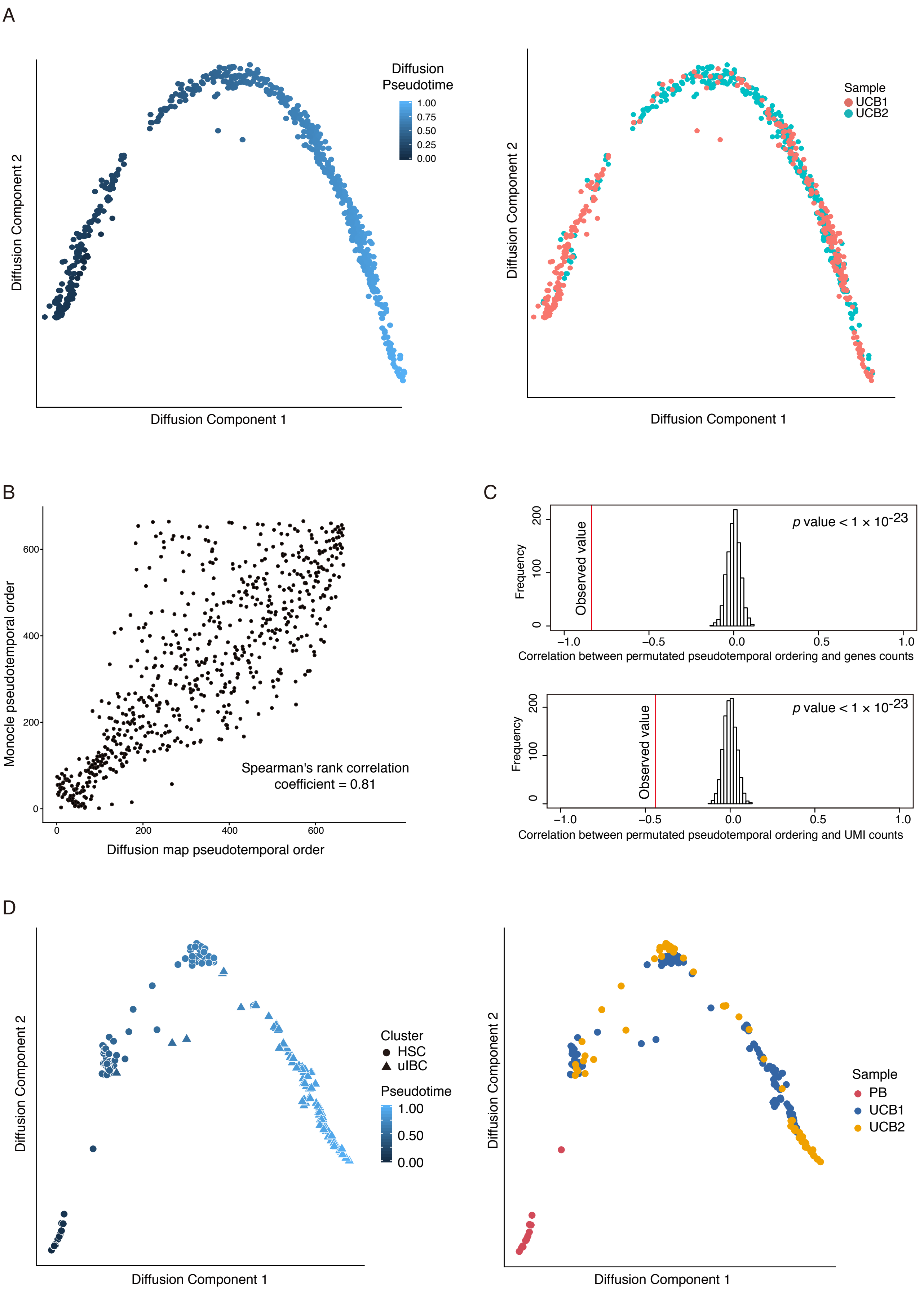

Supplementary Figure 6

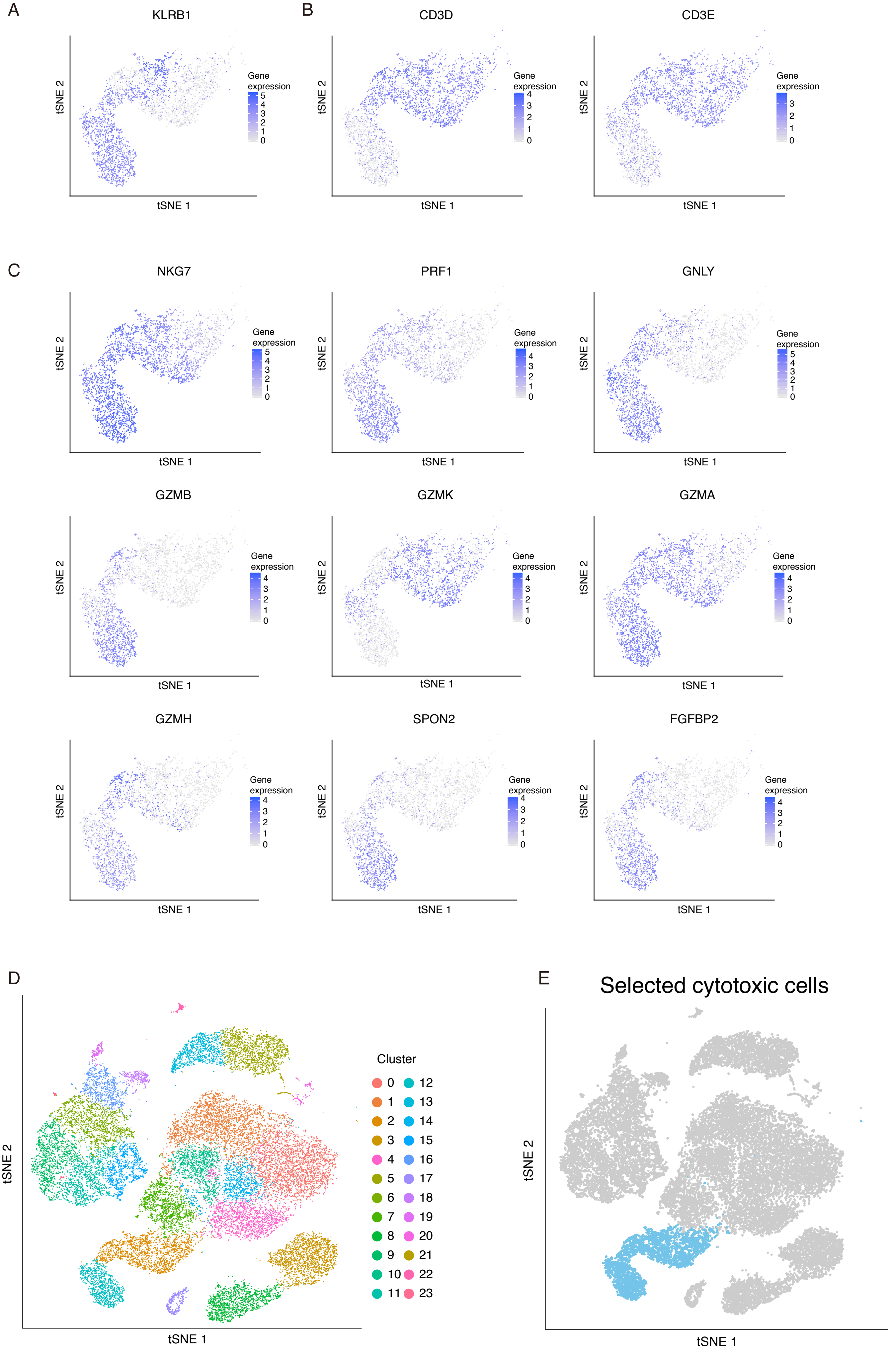

Supplementary Figure 7

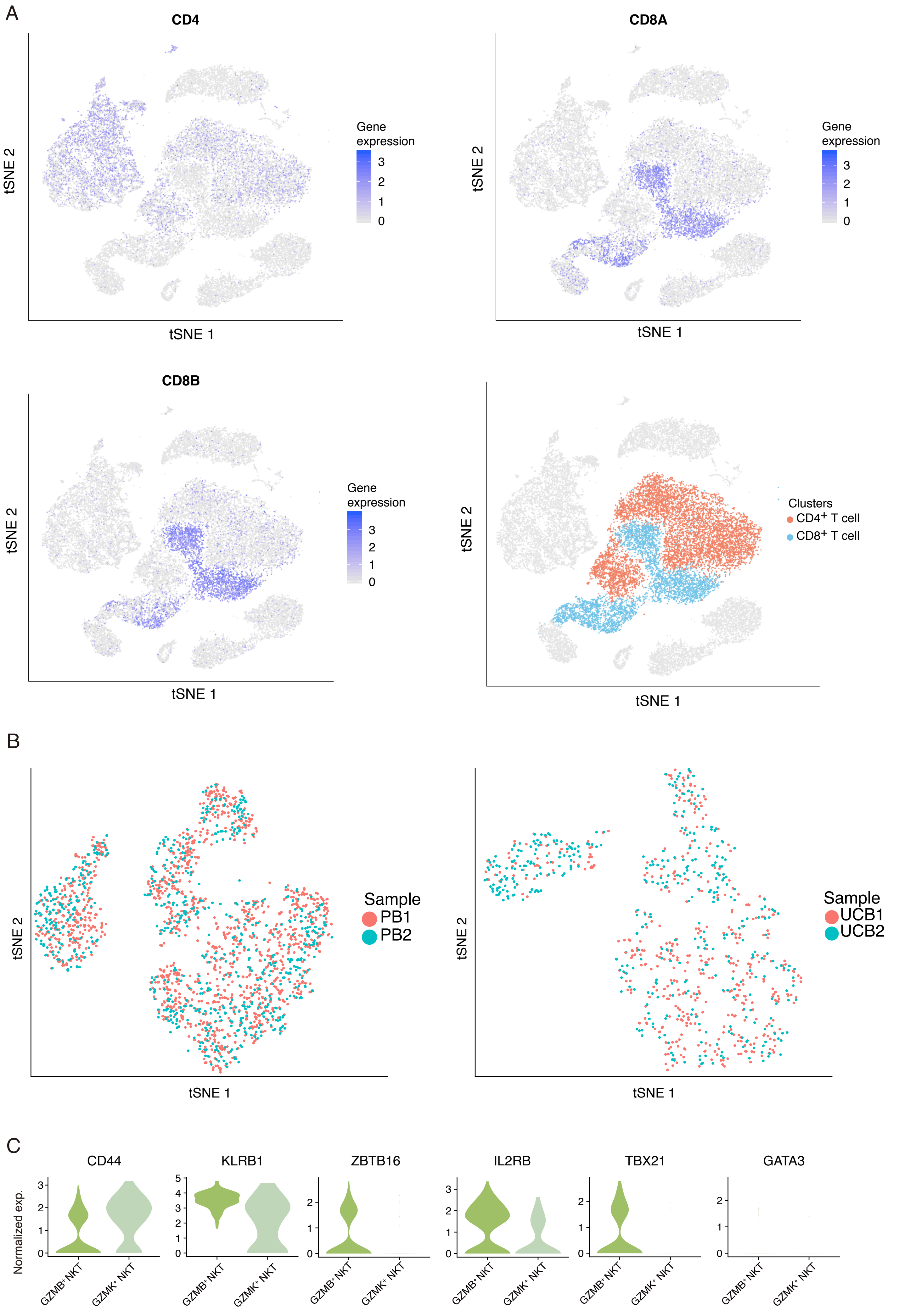

Supplement: Supplemental File [file giz047_supplemental_file.pdf]
