# Supplementary material for: Single-cell transcriptomic landscape of nucleated cells in umbilical cord blood
Source: Gigascience. 2019 May 2;8(5):giz047. doi: 10.1093/gigascience/giz047 (PMC6497034; doi:10.1093/gigascience/giz047)
Supplement: Response_to_Reviewer_Comments_Original_Submission.pdf [file giz047_response_to_reviewer_comments_original_submission.pdf]

# Author's Response To Reviewer Comments

Close

Response to Reviewers' Comments:

Reviewer #1:

This revised manuscript is significantly improved over the inital submission, and I am happy to see that the authors have been able to address most of the concerns I previously raised.
While I still think that the paper would benefit from additional biological replicates,
the presented analyses are technically sound and the results are in line with the conclusions.
Therefore, in my opinion, the revised paper can be considered for publication.

We appreciate the Reviewer's comments on the revised manuscript. Single-cell profiling with more samples and higher throughput in cord blood is essential to further our understandings on this unique tissue type. We believe that the current report serves as valuable resource and scaffold for future large-scale studies.

Minor comments:
1. CD3+KLRB1- cells are referred to as Cytotoxic T lymphocytes (CTL), but some of these are probably CD4+ T helpers.

We believe that the CD3+KLRB1- cells are indeed cytotoxic T lymphocytes (CTL) because all of the selected T cells that we profiled in Fig.4 belong to CD8 positive T cell clusters. We have now added new Supplementary Figure S7A to demonstrate the expression patterns of CD4 and CD8, as well as the T cell subsets that they represented.

2. Granzymes seem to be very useful markers in scRNA-seq, and have been mentioned in several previous papers, including:
Zheng C et al. Cell 2017 PMID: 28622514
Azizi E et al. Cell 2018 PMID: 29961579
Zhang L et al. Nature 2018 "Lineage tracking reveals dynamic relationships of T cells in colorectal cancer"
It would be very useful to try to reconcile the findings with such works, if possible.

We have taken the Reviewer's advice and discussed the consistency of the related findings in the revised Discussion section (Page 18-19).

3. In my opinion, and in light of the references above, the division to GZMK+ and GZMB+ T cells is likely to reflect different consecutive activation states that might be to some extent reversible. I would therefore suggest reconsidering the use of term cell "subtype" in this context, as to me it implies a more fixed state.

Our findings regarding expression of granzymes not only related to different T cells, but also NK cells and NKT cells, which certainly belonged to distinct lineages. To avoid confusion, we used "cell subtype" consistently through these parts of the manuscript. However, we agree with the Reviewer on the potential reversibility between these states within each lineage, which was now mentioned in the revised Discussion section (Page 18-19).

4. I would recommend one final round of proofreading before publication

Done.

Reviewer #2:

The authors show the transcriptomic landscape of 17637 nucleated cells from umbilical cord blood of two donors. They use these transcriptomic signatures to infer cell types and subtypes, discover rare cell types and project developmental trajectories. Thus, they have created a comprehensive transcriptomic map of the UCB in single-cell resolution.
We found significant problems with the presented data in the original draft of the paper but during the revision a lot of information has been added and all of our concerns have been addressed.

We thank the Reviewer for the insightful suggestions which helped improving this manuscript.

Reviewer #3:

In this revised version of the paper, Zhao et al. have presented an extensively revised manuscript in which they addressed my main concerns, specially those regarding batch effects across samples. Still, there are some open questions that need to be addressed.

We appreciate the enthusiasm of the reviewer. Below, we address the specific issues raised.

In page 6, the authors state that "The abundance of the common cell types also varied in PB versus that of UCB, suggesting a specific immunological capacity of UCB (Fig. 1C, Supplementary Fig. 4B)." Taking into account the large differences in cellular proportions observed between UCB samples, I do not think the authors can make a claim about the meaning. If they want to make the claim in the paper, they need to provide additional UCB samples showing that the cell proportions across UCB datasets are robust and perform additional statistical test showing it. Furthermore, the author should write the percentage of cells in each cluster next to the total amount of cells in Figure S4B.

We agree that this claim lacked substantial support and thus has removed it in the text. The percentages of cell types have been added in new Supplementary Figure 4B.

In page 8, the authors say that "we modeled gene expression along the Monocle2-inferred trajectory to identify genes characterized by a wave-like pattern". How this genes were identified, and how the genes shown in Fig 2B were selected, is not described anywhere in the paper.

As we stated in the Methods section, NRBCs were ordered according to the pseudotime deduced by 1,859 ordering genes excluding ribosomal protein transcripts that are differentially expressed (FDR < 0.05) by "clusterCells" function in Monocle2 package. Specifically, Monocle uses the VGAM package to model a gene's expression level as a smooth, nonlinear function of pseudotime. The genes that change as a function of pseudotime were further clustered to allow visualization of modules of genes that co-vary across pseudotime. In each of the three identified modules, we hand-picked the representative RBC effector and known development-relevant genes for the heatmap plots. This information has been added to the new Methods section (Page 27).

In page 9, the authors say that "a gradual decrease in the numbers of RNA molecules (represented by UMI) (Fig. 2D) and expressed genes (Fig. 2E) across the pseudotime axis was observed, reflecting the decrease of global gene expression activity due to the NRBC enucleation". Given the intrinsic heterogeneity in the number of genes and UMI captured, this claim is not well supported. The authors should perform a statistical analysis showing if the correlation between Genes and UMI counts and Pseudotime is significant and compared it to the correlation obtained by permuting the pseudotemporal ordering of cells. Additionally, they should show the distribution of UMIs and Genes for the different cell clusters and assess if the variability in UMI and Gene counts in this cluster is actually bigger than in other clusters.

We apologize for the missing statistical analyses in this part. We observed significant correlation between the Monocle-deduced pseudotemporal ordering with the gene count and with the UMI count, scoring Spearman's Correlation Coefficient (SCC) -0.84 (p value=6.81x10-178) and -0.44 (p value=6.74x10-33), respectively. We have added this information in the plots (new Figure 2D,E). Furthermore, we also performed permutation testing with 1,000-time simulated pseudotemporal ordering against the gene count and UMI count, and found that the observed SCCs significantly deviated from the simulated distribution (both p value<10-23 for gene and UMI) (new Supplementary Figure 5C), demonstrating that the decrease of UMI and gene detected is in fact in correlation with the inferred pseudotemporal trajectories.
The Reviewer also argues that for the changes of UMI and gene counts to be causal and specific to NRBC enucleation, such changes in other cell types should be in a much smaller scale. We believe this is not

necessarily the case because of the following reasons. (1) It is entirely possible for other cell types to have equally or more dramatic changes in transcription activity and expressed repertoire of genes. For examples, T cells undergo systemic transcription activation and deactivation when transitioning from naïve to effector and exhausted states; B cells highly focus on expressing immunoglobin genes after maturation to plasma cells. (2) The observed range of changes in NRBC may not fully represent the whole course of enucleation but rather a limited window during the development progress. Thus, we think the variability cannot be used to support our conclusion in this case.

Minor comments:
- the sequencing data is not publicly available in any repository

The data reported in this study are deposited in the CNGB Nucleotide Sequence Archive (CNSA) (CNSA: https://db.cngb.org/cnsa/) with accession number: CNP0000090. The aligned sequences in CRAM format and the gene expression matrix for every single cell have also been uploaded to GigaDB. This statement has been put in the manuscript.

- page 2 line 17, missing of preposition "Progenitor cells in UCB also consist OF two subpopulations"
- page 4, the number of cells reported for the UCB samples differs from that in the methods section.

Corrections have been made.

- page 7, the sentence "By ordering NRBCs with differential genes identified within the clusters, we employed Monocle2 software to deduce a pseudotime axis that suggested a gradual change of cellular state [28] (Methods)." is confusing.

We now have rewritten this sentence as: "We employed Monocle software to identify differential genes amongst NRBCs and deduced a pseudotemporal ordering of the cells that suggested a gradual change of cellular state." (Page 7)

- page 9, line 34, hematopoietic stem cells (HSCs) are not marked in Fig 1A o Fig S4A. Please clarify which are these cells.

HSCs belonged to the general "progenitor cell" category in Fig 1A and Fig S4A.

- page 9, line 34, Cite also Fig S4A

Done.

- page 9, line 53, Fig 3A should be Fig 3B

Corrected.

- page 11, the sentence "While a gradual identity shifting from HSC to uIBC was observed on the first diffusion component, the trajectory did not show a conclusive bifurcation of uIBC towards the differentiated polarity, likely due to the limited cell abundance (Supplementary Fig. 5C)." is confusing and is not clear what the authors' mean.

Due to the bi-potent characteristics of uIBC, these cells are expected to display bifurcated downstream developmental trajectories towards mast cells and basophil, which was surprisingly not observed in the diffusion map. We speculate that this is due to the limited cell number. We have rephrased this sentence as: "While a gradual shifting of identities from HSC to uIBC was observed on the first diffusion component, the uIBC side of the trajectory did not show a conclusive bifurcation towards mast cell and basophil lineages, likely due to the limited cell number." (Page 11)

- page 22, the authors need to justify why they use differnt cutoffs in number of genes and mitocondrial genes and number of UMIs to filter out cells across datasets. They also need to justify why they select 10 PCs to perform the clustering and tSNE.

The cutoffs were set based on the distributions of gene number, UMI count and mitochondria percentages specific to each sample, which is expected to be different across samples. As the cutoffs all aim to filter obvious outliers (i.e. ~2-5%), such arbitrary thresholding has become a common practice adopted by single cell analysis pipelines such as Seurat.

The included number of PCs was set based the significant decrease of variances across the ordered PCs. It is important to note that this step is only meant to isolate the NRBCs from the rest of the cells, thus selecting more or less PCs does not significantly change the outcome.

- page 23, change "observed best performance while chose 15 canonical vectors" to "observed best performance when we used 15 canonical vectors"
- page 26, line 23, change "red blood cells used" to "red blood cells were used"
- change bargraph to barplot in the legend of Figure S2
- Figure 2D&E. Clarify y-axis labelling
- Figure 3C. Describe the units and what is measured in the expression scale

All above errors have been corrected in the revised manuscript.

- Figure 3B, change the yaxis of the plots for MME abd CEBPA plots so that the distributions are seen. Include labelling for the y-axis.

All gene expression is normalized and directly comparable, thus we plot gene expression distribution in a similar scale. The expression of MME1 and CEBPA was almost undetectable, which was part of the basis that determined the identify of uIBC.

- Figure 4, use the same colors to label the same populations in UCB and PB datasets so that is easier to compare the populations

We did use the same colors. Please note the different cell identities in PB and UCB.

- Figure 4E. Include labelling for the y-axis.
- Figure S5. Typo "mmonocle" in the y-axis label

Corrections have been made.

Close
