# Supplementary material for: Single-cell transcriptomic landscape of nucleated cells in umbilical cord blood
Source: Gigascience. 2019 May 2;8(5):giz047. doi: 10.1093/gigascience/giz047 (PMC6497034; doi:10.1093/gigascience/giz047)
Supplement: Response_to_Reviewer_Comments_Revision_1.pdf [file giz047_response_to_reviewer_comments_revision_1.pdf]

Author's Response To Reviewer Comments

Close

We have edited and responded to all the comments from the copy editors, and provide a revised edition. Additionally, we have found some errors in the original supplementary figure 3, and provide a updated figure.

Close
